# High-entropy high-hardness metal carbides discovered by entropy descriptors

**DOI:** 10.1038/s41467-018-07160-7

**Published:** 2018-11-26

**Authors:** Pranab Sarker, Tyler Harrington, Cormac Toher, Corey Oses, Mojtaba Samiee, Jon-Paul Maria, Donald W. Brenner, Kenneth S. Vecchio, Stefano Curtarolo

**Affiliations:** 1 Department of Mechanical Engineering and Materials Science, Duke University, Durham, NC 27708 USA; 2 Materials Science and Engineering Program, University of California, San Diego, La Jolla, CA 92093 USA; 3 Department of Nanoengineering, University of California, San Diego, La Jolla, CA 92093 USA; 4 Department of Materials Science and Engineering, North Carolina State University, Raleigh, NC 27695 USA; 5 Materials Science, Electrical Engineering, Physics and Chemistry, Duke University, Durham, NC 27708 USA; 6 Fritz-Haber-Institut der Max-Planck-Gesellschaft, 14195 Berlin-Dahlem, Germany

## Abstract

High-entropy materials have attracted considerable interest due to the combination of useful properties and promising applications. Predicting their formation remains the major hindrance to the discovery of new systems. Here we propose a descriptor—entropy forming ability—for addressing synthesizability from first principles. The formalism, based on the energy distribution spectrum of randomized calculations, captures the accessibility of equally-sampled states near the ground state and quantifies configurational disorder capable of stabilizing high-entropy homogeneous phases. The methodology is applied to disordered refractory 5-metal carbides—promising candidates for high-hardness applications. The descriptor correctly predicts the ease with which compositions can be experimentally synthesized as rock-salt high-entropy homogeneous phases, validating the ansatz, and in some cases, going beyond intuition. Several of these materials exhibit hardness up to 50% higher than rule of mixtures estimations. The entropy descriptor method has the potential to accelerate the search for high-entropy systems by rationally combining first principles with experimental synthesis and characterization.

## Abstract (web summary)

The overwhelming number of possible high-entropy materials represents a big challenge for predicting their existence. Here, the authors introduce an entropy-forming-ability descriptor capturing the synthesizability of these systems, and apply the model to the discovery of new refractory metal carbides.

## Introduction

High-entropy materials having a highly disordered homogeneous crystalline single phase (potentially stabilized entirely by entropic contributions) continue to attract a great deal of research interest^1–3^. Remarkable properties have been reported: high strength (yield stress >1 GPa) combined with ductility^4–11^, hardness^5,12,13^, superconductivity^14^, colossal dielectric constant^15^, and superionic conductivity^16^. Entropy is thought to play a key-stabilizing role in high-entropy alloys^1^, entropy-stabilized oxides^17,18^, high-entropy borides^19^, and high-entropy carbides^20–23^. The latter three classes consist of disordered metal cation sublattices with several species at equi-concentration combined with oxide^15,17,18,24^, boride^19^, or carbide^20–23^ anion sublattices. These systems offer the potential to combine excellent thermo-mechanical properties and resilient thermodynamic stability given by entropy stabilization with the higher oxidation resistance of ceramics^25^. The resistance of disordered carbides to extreme heat^26–28^, oxidation^23^, and wear makes them promising ultra-high-temperature ceramics for thermal protection coatings in aerospace applications^29^, and as high-hardness, relatively low-density high-performance drill bits and cutting tools in mining and industry.

Super-hard transition metal carbides have been known since the 1930s to exhibit significant levels of solid solution^26,30,31^, and to display high melting temperatures^26,27^. Ta_*x*_Hf_1−*x*_C forms a homogeneous solid solution across all composition ranges^28,32,33^, with Ta_4_HfC_5_ exhibiting one of the highest experimentally reported melting points ($T_{\mathrm{m}}\sim 4263\,{\mathrm{K}}$^26,27^). In this case, the two refractory metals randomly populate one of the two rock-salt sublattices^28^. More recent measurements indicate that the maximum melting point of 4232 K occurs without Ta at the composition HfC_0.98_^34^. Investigation of new carbide compositions will help elucidate the high temperature behavior of these materials, and will provide an avenue to settle the discrepancies in the experimental literature. To discover materials with even more advantageous properties, including increased thermal stability, enhanced strength and hardness, and improved oxidation resistance^23^, more species and configurations have to be considered. Unfortunately, the lack of a rational, effective, and rapid method to find and characterize the disordered crystalline phase makes it impossible to pinpoint the right combination of species/compositions and the discovery continues by slow and relatively expensive trial and error.

Computationally, the hindrance in in-silico disordered materials development can be attributed to entropy—a very difficult quantity to parameterize when searching through the immense space of candidates (even with efficient computational methods, e.g., Monte Carlo and ab-initio lattice energies in the Wang-Landau^35^ or nested sampling^36^ formalisms). CALPHAD has also been applied successfully^2,13,37–39^, although it is dependent on the availability of sufficient experimental data. This is the perfect challenge for ab-initio high-throughput computing^40^ as long as reasonable entropy descriptors—the set of parameters capturing the underlying mechanism of a materials property—can be found.

In this article, we undertake the challenge by formulating an entropy-forming-ability (EFA) descriptor. It captures the relative propensity of a material to form a high-entropy single-phase crystal by measuring the energy distribution (spectrum) of configurationally randomized calculations up to a given unit-cell size. A narrow spectrum implies a low energy cost for accessing metastable configurations, hence promoting randomness (i.e., entropy) into the system (high-EFA) at finite temperature. In contrast, a wide spectrum suggests a composition with a high energy barrier for introducing different configurations (low-EFA), and thus with a strong preference for ordered phases. The method is benchmarked by the matrix of possible carbides. Given a set of eight refractory metals (Hf, Nb, Mo, Ta, Ti, V, W, and Zr) plus carbon, the formalism predicts the matrix of synthesizable five-metal high-entropy carbides. Candidates are then experimentally prepared, leading to a novel class of systems. In particular, it is demonstrated that the descriptor is capable of reliably distinguishing between the compositions that easily form homogeneous single phases and the ones that do not, including identifying compositions that form single phases despite incorporating multiple binary carbide precursors with different structures and stoichiometric ratios. Note that because of the differing stoichiometries of the non-rock-salt phase binary carbide precursors Mo_2_C and W_2_C, the compositions listed in this work are nominal. There are extensive carbon vacancies in the anion sublattice in the synthesized materials, further contributing to the configurational entropy. Several of these materials display enhanced mechanical properties, e.g., Vickers hardness up to 50% higher than predicted by a rule of mixtures (ROM). Thus, this class of materials has strong potential for industrial uses where dense and wear-resistant impactors are needed, particularly for extreme temperature applications. The successful outcome demonstrates the strength of the synergy between thermodynamics, high-throughput computation, and experimental synthesis.

## Results

### EFA formalism

To accelerate the search in the chemical space, the entropy content of a compound is estimated from the energy distribution spectrum of metastable configurations above the zero-temperature ground state. At finite *T*, any disordered state can be present with a probability given by the Boltzmann distribution and the state’s degeneracy. Note that configurations are randomly sampled up to a given unit-cell size: the larger the size, the more accurately the spectrum represents the real thermodynamic density of states.

The energy distribution (*H*_*i*_) spectrum can be quantitatively characterized by its standard deviation *σ*, so that the *σ* becomes the descriptor for *S*: the smaller *σ*, the larger *S*. The descriptor for an *N*-species system, called the EFA, is defined as the inverse of the *σ* of the energy spectrum above the ground state of the *N*-system at zero temperature:

1 $${\mathrm{EFA}}(N) \equiv \left\{ {\sigma \left[ {{\mathrm{spectrum}}(H_i(N))} \right]_{T = 0}} \right\}^{ - 1}$$

where

2 $$\sigma \left\{ {H_i(N)} \right\} = \sqrt {\frac{{\mathop {\sum}\limits_{i = 1}^n g_i(H_i - H_{{\mathrm{mix}}})^2}}{{\left( {\mathop {\sum}\limits_{i = 1}^n g_i} \right) - 1}}} ,$$

where *n* is the total number of sampled geometrical configurations and *g*_*i*_ are their degeneracies. *H*_mix_ is the mixed-phase enthalpy approximated by averaging the enthalpies *H*_*i*_ of the sampled configurations:

3 $$H_{{\mathrm{mix}}} = \frac{{\mathop {\sum}\limits_{i = 1}^n g_iH_i}}{{\mathop {\sum}\limits_{i = 1}^n g_i}}.$$

The broader the spectrum, the more energetically expensive it will be to introduce configurational disorder into the system, and thus the lower the EFA. EFA is measured in (eV/atom)^−1^.

### EFA calculation

**Fig. 1:**
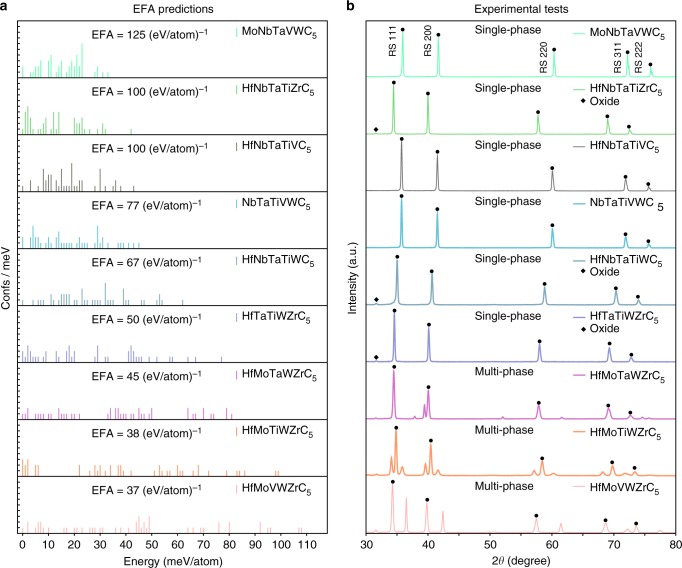
Schematics of high-entropy carbides predictions. **a** The energy distribution of different configurations of the 9 five-metal carbides: MoNbTaVWC_5_, HfNbTaTiZrC_5_, HfNbTaTiVC_5_, NbTaTiVWC_5_, HfNbTaTiWC_5_, HfTaTiWZrC_5_, HfMoTaWZrC_5_, HfMoTiWZrC_5_, and HfMoVWZrC_5_; spectrum is shifted so that the lowest energy configuration for each composition is at zero. The energy spectrum for each composition indicates its propensity to form the high-entropy single phase: the narrower the distribution, the more likely it is to form a high-entropy single phase at finite *T*. **b** The X-ray diffraction patterns for the same 9 five-metal carbides, where the first six compositions exhibit only the desired fcc structure peaks, whereas the additional peaks for remaining three compositions indicate the presence of secondary phases. The small peaks at 2*θ* = 31.7°, marked by the diamond symbol in the spectra of HfNbTaTiZrC_5_, HfNbTaTiWC_5_ and HfTaTiWZrC_5_, are from the (111) plane of a monoclinic (Hf,Zr)O_2_ phase that remains due to processing

A total of 56 five-metal compositions can be generated using the eight refractory metals (8!/5!3! = 56) of interest (Hf, Nb, Mo, Ta, Ti, V, W, and Zr). For each composition, the Hermite normal form superlattices of the Automatic FLOW (AFLOW) partial occupation (AFLOW-POCC) method^41^ generate 49 distinct 10-atom-cell configurations, resulting in a total of 2744 configurations needed to determine the EFA of this composition space (Methods section). The ab-initio calculated EFA values for the full set of 56 five-metal compositions are provided in Table 1. Nine candidates are chosen from this list for experimental synthesis and investigation: (i) the three candidates with the highest value of EFA (MoNbTaVWC_5_ (EFA = 125 (eV/atom)^−1^), HfNbTaTiZrC_5_ (EFA = 100 (eV/atom)^−1^), HfNbTaTiVC_5_ (EFA = 100 (eV/atom)^−1^), high probability of forming high-entropy single phases), (ii) the two candidates with the lowest value of EFA (HfMoVWZrC_5_ (37 (eV/atom)^−1^), HfMoTiWZrC_5_ (38 (eV/atom)^−1^), low probability of forming high-entropy single phases), and (iii) four chosen at random with intermediate EFA (NbTaTiVWC_5_ (77 (eV/atom)^−1^), HfNbTaTiWC_5_ (67 (eV/atom)^−1^), HfTaTiWZrC_5_ (50 (eV/atom)^−1^), and HfMoTaWZrC_5_ (45 (eV/atom)^−1^)). Figure 1a shows the energy distribution and EFA values obtained ab initio from configurations generated with AFLOW for the nine chosen systems. For MoNbTaVWC_5_, HfNbTaTiZrC_5_, and HfNbTaTiVC_5_, most of the configurations are within 20 meV/atom of the lowest energy state, and the distributions have an EFA of at least 100 (eV/atom)^−1^. Therefore, at finite temperature, most of the configurations should have a high probability of being formed, so that a high level of configurational randomness is expected to be accessible in the three systems, making them promising candidates to form a high-entropy homogeneous single phase. Achieving a similar level of configurational randomness would be progressively more difficult in NbTaTiVWC_5_, HfNbTaTiWC_5_, and HfTaTiWZrC_5_ as the different configurations display a broader energy distribution, with EFAs ranging from 50 to 77 (eV/atom)^−1^. A higher energy cost is needed to incorporate configurational entropy into these three compositions, so forming a homogeneous single phase will be more difficult. For HfMoTaWZrC_5_, HfMoTiWZrC_5_, and HfMoVWZrC_5_, the spread of energies for the configurations is very wide, with EFA values from 45 down to 37 (eV/atom)^−1^. These materials would be expected to be very difficult to synthesize as a homogeneous single phase.

**Table 1:** Results for the calculated entropy-forming-ability (EFA) descriptor, energetic distance from six-dimensional convex hull (Δ*H*_f_) and vibrational free energy at 2000 K (Δ*F*_vib_) for the five-metal carbide systems, arranged in descending order of EFA

System	EFA	Δ*H*_f_	Δ*F*_vib_	Exp.	*ε*	System	EFA	Δ*H*_f_	Δ*F*_vib_	Exp.	*ε*	System	EFA	Δ*H*_f_	Δ*F*_vib_	Exp.	*ε*
MoNbTaVWC_5_	125	156	−14	S	0.063	HfNbTaVWC_5_	67	110				TaTiVWZrC_5_	50	96			
HfNbTaTiZrC_5_	100	19	−12	S	0.094	HfMoTaTiVC_5_	67	82				NbTiVWZrC_5_	50	93			
HfNbTaTiVC_5_	100	56	−31	S	0.107	HfMoNbTiZrC_5_	67	53				HfMoTiVZrC_5_	50	96			
MoNbTaTiVC_5_	100	82				MoNbTaWZrC_5_	63	133				HfMoTaVZrC_5_	50	92			
NbTaTiVZrC_5_	83	64				HfMoTaTiZrC_5_	63	55				HfMoNbVZrC_5_	50	89			
HfMoNbTaTiC_5_	83	48				NbTaTiWZrC_5_	59	61				MoTaVWZrC_5_	48	148			
NbTaTiVWC_5_	77	92	−19	S	0.124	MoTaTiVZrC_5_	59	92				MoTaTiWZrC_5_	48	94			
MoNbTaTiWC_5_	77	111				MoNbTiVZrC_5_	59	87				MoNbVWZrC_5_	48	146			
MoNbTiVWC_5_	71	122				MoNbTaVZrC_5_	59	108				MoNbTiWZrC_5_	48	89			
MoNbTaTiZrC_5_	71	57				HfNbTiVWC_5_	59	81				HfMoNbWZrC_5_	48	101			
HfTaTiVZrC_5_	71	73				HfNbTaWZrC_5_	59	53				HfTiVWZrC_5_	45	99			
HfNbTiVZrC_5_	71	73				NbTaVWZrC_5_	56	119				HfNbVWZrC_5_	45	94			
HfMoNbTiVC_5_	71	77				HfTaTiVWC_5_	56	84				HfMoTiVWC_5_	45	97			
HfMoNbTaZrC_5_	71	48				HfMoTaVWC_5_	56	139				HfMoTaWZrC_5_	45	105		M	0.271
HfMoNbTaWC_5_	71	126				HfMoNbVWC_5_	56	137				HfTaVWZrC_5_	43	97			
HfMoNbTaVC_5_	71	99				HfNbTiWZrC_5_	53	56				MoTiVWZrC_5_	40	107			
HfNbTaTiWC_5_	67	53	~0	S	0.171	HfMoTaTiWC_5_	53	84				HfMoTiWZrC_5_	38	83		M	0.315
MoTaTiVWC_5_	67	128				HfMoNbTiWC_5_	53	81				HfMoVWZrC_5_	37	141		M	0.325
HfNbTaVZrC_5_	67	60				HfTaTiWZrC_5_	50	59	~0	S	0.169						

Nine compositions are selected for experimental investigation. The lattice distortion, *ε*, is obtained from the peak broadening in XRD. *S*: single-phase formed; *M*: multi-phase formed in experiment. Note that the compositions listed here are nominal, and the actual synthesized compositions can vary due to the presence of carbon vacancies in the anion sublattice. Units: EFA in (eV/atom)^−1^; Δ*H*_f_ and Δ*F*_vib_ in (meV/atom); and *ε* in %

### Competing ordered phases

The phase diagrams for the five-metal carbide systems were generated to investigate the existence of binary and ternary ordered structures that could compete with the formation of the high-entropy single phase. First, prototypes for experimentally reported binary and ternary carbide structures^42,43^ are used to calculate the formation enthalpies of additional ordered phases for the AFLOW database. The results are used to generate the convex hull phase diagrams for all 56 compositions using the AFLOW-CHULL module^44^. The relevant binary and ternary convex hulls are illustrated in Supplementary Figures 1–36. The distance along the enthalpy axis of the lowest energy AFLOW-POCC configuration from the convex hull, Δ*H*_f_, is listed in Table 1 (the decomposition reaction products are summarized in Supplementary Table 2). A rough estimation of the synthesis temperature *T*_s_ (see the analogous Entropy-Stabilized Oxides case, Fig. 2 of ref.^17^)—can be calculated by dividing Δ*H*_f_ by the ideal configuration entropy (Supplementary Table 1, with the ideal entropy per atom evaluated as 0.5*k*_B_ × log0.2, since *k*_B_ × log0.2 is the entropy per metal carbide atomic pair). A precise characterization of the disorder requires more expensive approaches, such as the LTVC method^45^, and is beyond the scope of this article. The highest temperature is 2254 K, which is less than the synthesis temperature of 2200 °C (2473 K), indicating that during sintering the disordered phases are thermodynamically accessible with respect to decomposition into ordered compounds. In analogy to the formation of metallic glasses where energetic confusion obstructs crystalline growth^46,47^ once the temperature is reduced, systems with high EFA remain locked into ensembles of highly degenerate configurations, retaining the disorder achieved at high temperature. Hence, EFA provides a measure of the relative synthesizability of the disordered composition.

**Fig. 2:**
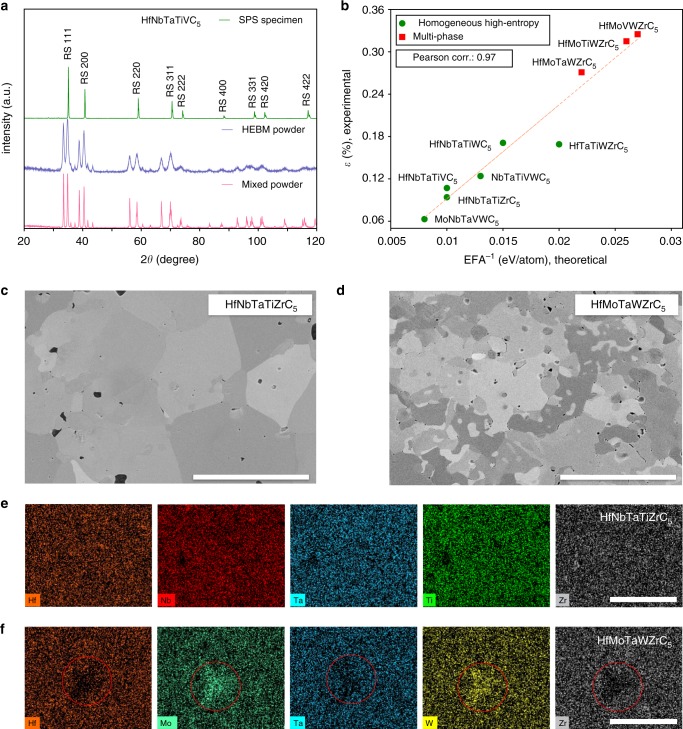
Experimental results for high-entropy carbides synthesis and characterization. **a** Progression of a sample of HfNbTaTiVC_5_ through each processing step: hand mixing (magenta spectrum, bottom), ball milling (blue spectrum, center), and spark plasma sintering (green spectrum, top), depicting the evolution towards the desired rock-salt crystal structure (*a*_exp_ = 4.42 Å). **b** Linear relationship between EFA^−1^ and the distortion of experimental lattice parameters *ε*. Green circles and red squares indicate homogeneous high-entropy single- and multi-phase compounds, respectively. **c**, **d** Electron micrographs of single-phase HfNbTaTiZrC_5_ and multi-phase HfMoTaWZrC_5_ specimens. **e**, **f** Selected EDS compositional maps of the HfNbTaTiZrC_5_ and HfMoTaWZrC_5_ specimens. The micrographs show the presence of the secondary phase in HfMoTaWZrC_5_ (circles) that is also present in XRD results, which is revealed to be a W- and Mo-rich phase. Scale bars, 10 μm (**c**–**f**)

### From enthalpy to entropy

It is important to consider that the metal carbide precursors have very strong covalent/ionic bonds, and are therefore enthalpy stabilized^48^. However, the same might not be the case for their mixture. In fact, a statistical analysis of the AFLOW.org enthalpies^44^ indicates that the gain in formation enthalpy by adding mixing species, Δ*H*_f_(*N* + 1) − Δ*H*_f_(*N*), decreases with *N*, and can easily be overcome by the monotonic increase in entropy-gain for the disordered systems (to go from order to complete or partial disorder). In the AFLOW analysis, the threshold between low- and high-entropy systems is around four mixing species. To have completely entropy-stabilized materials, five mixing species are required (similar to the Entropy-Stabilized Oxides^17^). For carbides in which only the metal-sublattice is randomly populated, five metals should be enough to achieve carbide entropy stabilization, especially at equi-composition. Notably, if other sublattices were also allowed to contain disorder, (e.g., reciprocal systems like Ta_*x*_Hf_1−*x*_C_1−*y*_^26,27^), then the overall number of species might reduce. In this example, entropy was increased by introducing point defects (vacancies)—a promising strategy to improve high temperature performance. In HfC_1−*x*_, the reduction of C to sub-stoichiometry enhances the stabilizing effect of the configurational entropy on the solid phase, offsetting its Gibbs free energy (vacancies can only exist in the solid phase) leading to an overall increase in melting point (see ref.^28^).

### Experimental results

To validate the predictions, the nine chosen carbides are experimentally synthesized and characterized (see Methods section). The chemical homogeneity of each sample is measured using energy-dispersive X-ray spectroscopy (EDS), whereas the crystalline structure is determined via X-ray diffraction (XRD).

An example of the evolution of a sample of composition HfNbTaTiVC_5_ through each processing step is given in Fig. 2a, demonstrating the densification and homogenization into a single rock-salt structure. At least three distinct precursor phases are distinguishable in the mixed powder pattern. Following ball milling, the individual phases are still present, however, the peaks are considerably broadened, which is due to particle size reduction and mechanical alloying. Following the final spark plasma sintering (SPS) step at 2200 °C, the sample consolidates into a bulk solid pellet of the desired single rock-salt phase indicating the successful synthesis of a high-entropy homogeneous carbide.

Results of XRD analysis for each sample following SPS at 2200 °C, presented in Fig. 1b, demonstrate that compositions MoNbTaVWC_5_, HfNbTaTiZrC_5_, HfNbTaTiVC_5_, HfNbTaTiWC_5_, NbTaTiVWC_5_, and HfTaTiWZrC_5_ (the top 6) only exhibit single fcc peaks of the desired high-entropy phase (rock-salt), whereas HfMoTaWZrC_5_, HfMoTiWZrC_5_, and HfMoVWZrC_5_ (the bottom 3) show multiple structures. The small peaks at 2*θ* = 31.7°, marked by the diamond symbol in the spectra of HfNbTaTiZrC_5_, HfNbTaTiWC_5_, and HfTaTiWZrC_5_ in Fig. 1b, are from the (111) plane of a monoclinic (Hf, Zr)O_2_ phase that remains due to processing. The volume fraction of this phase is <5% and does not significantly alter the composition of the carbide phase. The distinguishable second phase in HfMoTaWZrC_5_ is identified as a hexagonal phase. One and two secondary fcc phases are observed for HfMoVWZrC_5_ and HfMoTiWZrC_5_, respectively. Microstructure analysis and selected elemental mapping (Figs. 2c–d) confirm that the systems displaying single phases are chemically homogeneous, whereas the multi-phase samples undergo chemical segregation. For example, only grain orientation contrast is present in the HfNbTaTiZrC_5_ microstructure, and no indication of notable clustering or segregation is visible in its compositional maps. On the contrary, a clear chemical phase contrast is observable in the microstructure of the multi-phase HfMoTaWZrC_5_ sample, and the compositional maps demonstrate that the secondary phase, apparent in XRD, is W- and Mo-rich.

### Homogeneity analysis

Peak broadening in XRD patterns (Fig. 1b) is used to quantify the level of structural homogenization achieved in the samples. According to the Williamson–Hall formulation^49^, broadening in XRD is principally due to crystallite size (∝ 1/cos*θ*, *θ* = Bragg angle) and lattice strain (∝ 1/tan*θ*). For multi-component systems, significant broadening is expected to occur due to local lattice strains and variations in the interplanar spacings throughout the sample^50^. The latter can be attributed to the inhomogeneous distribution of the elements, the extent of which can be evaluated by applying the analysis to a multi-component system, which has only a single lattice structure and is assumed strain free^50^.

The lattice distortion of the rock-salt phase in the single-phase materials (or the most prevalent in the multi-phase ones) is determined by using the relationship between broadening *β*_S_ and Bragg angle *θ* (Methods section):

4 $$\beta _{\mathrm{S}}{\mathrm{cos}}\theta = 4\varepsilon {\mathrm{sin}}\theta + \frac{{K\lambda }}{D},$$

where *ε* is the lattice strain or variation in interplanar spacing due to chemical inhomogeneity, *K* is a constant (dependent on the grain shape), *λ* is the incident X-ray wavelength, and *D* is the crystallite size. Since materials are assumed strain free, *ε*—obtained by inverting Eq. 4—represents the relative variation of the lattice parameter due to inhomogeneity. Thus, *ε* is both a measure of homogeneity and of the effective mixing with respect to the ideal scenario. The results for the EFA descriptor, the experimental characterization, as well as the values for *ε* for all nine carbides compositions are given in Table 1. The values for *ε* range from 0.063% for MoNbTaVWC_5_ and 0.094% for HfNbTaTiZrC_5_ (the most homogeneous materials) to 0.325% for HfMoVWZrC_5_ (the least homogeneous material). Overall, the experimental findings agree well with the predictions of EFA descriptor, validate its ansatz and indicate a potential threshold for our model of five-metal carbides: EFA $\sim 50 {\mathrm{(eV/atom)}}^{ - 1} \Rightarrow$ homogeneous disordered single phase (high entropy).

### High-entropy synthesizability

The comparison between the EFA predictions and the homogeneity of the samples is analyzed in Fig. 2b. Although the Williamson–Hall formalism does not provide particularly accurate absolute values of *ε*, it is effective for comparing similarly processed samples, determining the relative homogeneity. The lattice distortion *ε* (capturing homogeneity) decreases linearly with the increase of EFA. The Pearson (linear) correlation of EFA^−1^ with *ε* is 0.97, whereas the Spearman (rank order) correlation is 0.98. As such, the EFA takes the role of an effective high-entropy synthesizability descriptor.

Three facts are relevant. (i) Intuitively, several of the carbide compositions that easily form a highly homogeneous phase, particularly HfNbTaTiVC_5_ and HfNbTaTiZrC_5_, come from binary precursors having the same structure and ratio of anions to cations as the final high-entropy material. (ii) Counterintuitively, the highest-EFA and most homogeneous phase MoNbTaVWC_5_ is made with two precursors having different structures and stoichiometric ratios from the high-entropy material, specifically orthorhombic *α*-Mo_2_C and hexagonal *α*-W_2_C, leading to a final sub-stoichiometric MoNbTaVWC_5−*x*_. The additional disorder provided by the presence of vacancies in the C-sublattice is advantageous: it allows further entropy stabilization, potentially increasing the melting point vis-à-vis the stoichiometric composition^28^. An investigation of the effect of carbon stoichiometry is clearly warranted, although it is outside of the scope of this study. (iii) For tungsten and molybdenum, metal-rich carbides are used because of the difficulties in obtaining molybdenum monocarbide (MoC) powder, or tungsten monocarbide (WC) powder in the particle sizes compatible with the other precursors, hindering consistent mixing, sintering and homogenization. It should be noted that additional samples of MoNbTaVWC_5_ were also synthesized using hexagonal WC with a smaller particle size, and the homogeneous rock-salt phase was again successfully obtained (see Supplementary Figure 37). The existence of phase-pure MoNbTaVWC_5_ indicates that, similar to the rock-salt binary carbides, the multi-component carbide is stable over a range of stoichiometry. From experimental/phenomenological grounds, the formation of such a phase is surprising. The equi-composition binary carbides MoC and WC have hexagonal ground states, and their rock-salt configurations have significantly higher formation enthalpy^51^. Considering these facts, there are no experimental indications that adding Mo and W would contribute to stabilizing the most homogeneous rock-salt five-metal carbide that was predicted by the EFA and later validated experimentally. The arguments demonstrate the advantage of a descriptor that quantifies the relative EFA over simple empirical/phenomenological rules, in that it correctly identifies this composition as having a high propensity to form a single phase, while simultaneously correctly predicting that several other systems having both Mo and W subcomponents undergo phase separation.

### Mechanical properties

The Vickers hardness, *H*_V_, and elastic modulus, *E*, of both the binary carbide precursors and the synthesized five-metal single-phase compositions are measured using nanoindentation, where the properties are extracted from load-displacement curves (see Supplementary Figure 38(a)). The binary precursor samples for these measurements are prepared and analyzed using the same protocol to ensure the validity of the comparisons (see Methods section for more details). The results for the five-metal compositions are in Table 2, whereas those for the binary carbides are listed in Table 3. It is found that, for the five-metal compositions, the measured *H*_V_ and *E* values exceed those predicted from a ROM based on the binary precursor measurements. The enhancement of the mechanical properties is particularly strong in the case of HfNbTaTiZrC_5_, where the measured *E* and *H*_V_ exceed the ROM predictions by 10 and 50%, respectively (see Supplementary Figure 38(b) for a comparison between the *H*_V_ results obtained from calculation, experiment, and ROM). Mass disorder is one possible source of the enhanced hardness: deformation is caused by dislocation movements and activation energy is absorbed and released at each lattice step. An ideal ordered system can be seen as a dislocation-wave-guide with matched (uniform) impedance along the path: propagation occurs without any relevant energy reflection and/or dispersion. This is not the case for disordered systems: mass inhomogeneity causes impedance mismatch, generating reflections and disturbing the transmission by dispersing (scattering) its group energy. Macroscopically the effect is seen as increased resistance to plastic deformations—more mechanical work is required—i.e., increase of hardness. Other possible causes of increased hardness include solid solution hardening^5,13,20^, where the atomic size mismatch results in lattice distortions, limiting the motion of dislocations necessary for plastic deformation; and changes in the slip systems and the ease with which slip can occur^20,52^.

**Table 2:** Results for mechanical properties (bulk: *B*, shear: *G*, and elastic moduli: *E*, and Vickers hardness: *H*_V_) for six single-phase high-entropy carbides

	*B*		*G*		*E*		*H*_v,Chen_	*H*_v,Teter_	*H*_v,Tian_	*H*_v,exp_
System	AFLOW (ROM)	Exp. (ROM)	AFLOW (ROM)	Exp. (ROM)	AFLOW (ROM)	Exp. (ROM)	AFLOW (ROM)	AFLOW (ROM)	AFLOW (ROM)	Exp. (ROM)
MoNbTaVWC_5_	312 (321)	278 (−)	183 (183)	226 (−)	460 (459)	533 ± 32 (−)	20 (20)	28 (28)	20 (20)	27 ± 3 (−)
HfNbTaTiZrC_5_	262 (267)	235 (232)	192 (165)	188 (184)	464 (455)	443 ± 40 (436 ± 30)	27 (25)	29 (28)	27 (25)	32 ± 2 (23 ± 2)
HfNbTaTiVC_5_	276 (279)	267 (239)	196 (196)	212 (189)	475 (476)	503 ± 40 (449 ± 30)	26 (26)	30 (30)	26 (26)	29 ± 3 (24 ± 2)
HfNbTaTiWC_5_	291 (296)	252 (−)	203 (186)	205 (−)	493 (459)	483 ± 24 (−)	26 (22)	31 (28)	26 (22)	31 ± 2 (−)
NbTaTiVWC_5_	305 (304)	253 (−)	199 (189)	206 (−)	490 (460)	485 ± 36 (−)	24 (22)	30 (29)	24 (23)	28 ± 2 (−)
HfTaTiWZrC_5_	274 (280)	246 (−)	191 (178)	200 (−)	466 (438)	473 ± 26 (−)	25 (21)	29 (27)	25 (21)	33 ± 2 (−)

Three different models: Chen et al. ^54^, Teter ^55^, and Tian et al. ^56^ are used to calculate theoretical hardness values (only for the two non-W-containing compositions) from *B* and *G*. The ROM values are obtained from the results in this work for rock-salt structure binary carbides, as listed in Table 3. Since MoC and WC do not form a stable rock-salt phase at room temperature, the experimental ROMs for Mo- and W-containing compositions are not available, as indicated by (–). Units: *B*, *G*, *E*, and *H*_V_ in (GPa)

Elastic properties are calculated using AFLOW^41,53^ for the five-metal compositions MoNbTaVWC_5_, HfNbTaTiZrC_5_, HfNbTaTiVC_5_, HfNbTaTiWC_5_, NbTaTiVWC_5_, and HfTaTiWZrC_5_ (Table 2) and their precursors. In general, results are within the experimentally reported ranges for the binary carbides (Table 3 in the Methods section). *H*_V_ values are estimated from the bulk and shear moduli using the models introduced by Chen et al.^54^, Teter^55^, and Tian et al.^56^. Computational models do not consider plastic deformation mechanisms in inhomogeneous systems and thus *H*_V_ predictions underestimate experiments, leading to results consistent with the ROM of binary carbides (Table 2). The outcome further corroborates that the experimentally observed enhancement of the mechanical properties is due to disorder.

**Table 3:** Results for mechanical properties (bulk: *B*, shear: *G*, and elastic: *E*, moduli, and Vickers hardness: *H*_V_) for eight rock-salt structure binary carbides

	*B*			*G*			*E*			*H*_V_				
System	AFLOW	Exp.	Exp.^a^	AFLOW	Exp.	Exp.^a^	AFLOW	Exp.	Exp.^a^	Chen	Teter	Tian	Exp.	Exp.^a^
HfC	239	241	223	186	179–193	181	443	316–461	428 ± 32	29	28	28	19–25	25 ± 2
MoC	335	—	—	152	—	—	396	—	—	12	23	13	27–83^b^	—
NbC	297	296–378	246	199	197–245	177	488	330–537	429 ± 46	25	30	25	19–25	17 ± 3
TaC	326	248–343	219	213	215–227	184	525	241–722	431 ± 44	25	32	25	16–23	14 ± 2
TiC	251	241	255	181	186	207	438	447–451	489 ± 13	26	27	25	32	31 ± 2
VC	283	389	250	199	157	196	484	268–420	465 ± 13	26	30	26	20–29	29 ± 1
WC	365	—	—	153	—	—	403	—	—	11	23	12	> 28^c^	—
ZrC	221	220	216	157	172	169	381	385–406	402 ± 13	23	28	22	23–25	24 ± 1

The AFLOW values are calculated using the Voigt-Reuss-Hill average and the Automatic Elasticity Library (AEL) module^53^, whereas *H*_V_ is estimated using three different models described in the literature. These results are compared with two sets of available measured data, obtained from the literature^72,73^ and the current experiments. Units: *B*, *G*, *E*, and *H*_V_ in (GPa)

^a^This work

^b^*α*-MoC_1−*x*_  + *η*-MoC + *γ*-MoC composite^74^

^c^WC_1−*x*_; *x* = 0.36–0.41^75^

### Vibrational contribution to formation free energy

The vibrational contributions to the formation Gibbs free energy, Δ*F*_vib_, at 2000 K are listed in Table 1 for the six compositions synthesized as a single phase. The vibrational free energies, *F*_vib_, are calculated using the Debye–Grüneisen model implemented in the AFLOW–Automatic GIBBS Library (AGL) module^57^, using the Poisson ratio calculated with AFLOW–Automatic Elasticity Library (AEL)^53^. The average *F*_vib_ for the five-metal compositions are calculated, weighted according to the Boltzmann distribution at 2000 K. The vibrational contribution to the formation Gibbs free energy, Δ*F*_vib_, for each composition is obtained from the difference between its average *F*_vib_ and the average *F*_vib_ of its component binary carbides. Δ*F*_vib_ at 2000 K ranges from ~0 meV/atom for HfNbTaTiWC_5_ to −31 meV/atom for HfNbTaTiVC_5_, which are significantly less than the total entropy contribution (mostly configurational plus vibrational) required to overcome the values of 50 meV/atom to 150 meV/atom for the formation enthalpy Δ*H*_f_. These results are in agreement with previous observations that the vibrational formation entropy is generally an order of magnitude smaller than the configuration entropy^39,58^.

## Discussion

In this article, an EFA descriptor has been developed for the purpose of capturing synthesizability of high-entropy materials. The framework has been applied to refractory metal carbides, leading to the prediction and subsequent experimental discovery of homogeneous high-entropy single phases. The method is able to quantitatively predict the relative propensity of each composition to form a homogeneous single phase, thus identifying the most promising candidates for experimental synthesis. In particular, the experiments validate the prediction that the composition MoNbTaVWC_5_ should have a very high propensity to form a homogeneous single phase, despite incorporating both Mo_2_C and W_2_C, which have different structures (hexagonal and/or orthorhombic instead of rock-salt) and stoichiometric ratios from the five-metal high-entropy material.

Furthermore, it is demonstrated that disorder enhances the mechanical properties of these materials: HfNbTaTiZrC_5_ and HfTaTiWZrC_5_ are measured to have hardness of 32 GPa (almost 50% higher than the ROM prediction) and 33 GPa, respectively, suggesting a new avenue for designing super-hard materials. The formalism could become the long-sought enabler of accelerated design for high-entropy functional materials with enhanced properties for a wide range of different technological applications.

## Methods

### Spectrum generation

The different possible configurations required to calculate the energy spectrum are generated using the AFLOW-POCC algorithm^41^ implemented within the AFLOW computational materials design framework^51,59,60^. The algorithm initially generates a superlattice of the minimum size necessary to obtain the required partial occupancies within some user-specified accuracy. For each unique superlattice, the AFLOW-POCC algorithm then generates the complete set of possible supercells using Hermite normal form matrices^41^. Non-unique supercell combinations are eliminated from the ensemble by first estimating the total energies of all configurations using a Universal Force Field^41,61^ based method, and then identifying duplicates from their identical energies.

### Structure generation

In the case of the high-entropy carbide^17^ systems investigated here, the AFLOW-POCC algorithm starts with the rock-salt crystal structure (spacegroup: $Fm\bar 3m,\# 225$; Pearson symbol: cF8; AFLOW Prototype: AB_cF8_225_a_b^62^) as the input parent lattice. Each anion site is occupied with a C atom (occupancy probability of 1.0), whereas the cation site is occupied by five different refractory metal elements, with a 0.2 occupancy probability for each. The AFLOW-POCC algorithm then generates a set of configurations (49 in total in the case of the rock-salt based five-metal carbide systems, once structural duplicates are excluded), each containing 10 atoms: one atom of each of the metals, along with five carbon atoms. This is the minimum cell size necessary to accurately reproduce the required stoichiometry. All configurations have *g*_*i*_ = 10, except for one where *g*_*i*_ = 120, so that $\mathop {\sum}\nolimits_{i = 1}^n g_i = 600$ for the rock-salt-based five-metal carbide systems. Note that computational demands increase significantly with the number of elements: AFLOW-POCC generates 522, 1793, and 7483 for six-, seven-, and eight-metal carbide compositions, respectively.

### Energies calculation

The energy of each configuration is calculated using density functional theory (Vienna Ab-initio Simulation Package^63^) within the AFLOW framework^59^ and the standard settings^60^. Each configuration is fully relaxed using the Perdew, Burke, Ernzerhof (PBE) parameterization of the generalized gradient approximation exchange-correlation functional^64^, projector augmented wave potentials, at least 8000 **k**-points per reciprocal atom (KPPRA), and a plane-wave cut-off of at least 1.4 times the cut-off values of constituent species’ pseudopotentials^60^. The formation enthalpy (*H*_f_) of each configuration along with the link to AFLOW.org entry page is provided in Supplementary Tables 4–10.

### Mechanical properties

Elastic properties are calculated using the AEL module^53^ of the AFLOW framework, which applies a set of independent directional normal and shear strains to the structure, and fits the resulting stress tensors to obtain the elastic constants. From this, the bulk: *B*, and shear: *G*, moduli are calculated in the Voigt, Reuss and Voigt-Reuss-Hill (VRH) approximations, with the average being used for the purposes of this work. The elastic or Young’s modulus: *E*, is calculated using the approximation *E* = 9*BG*/(3*B* + *G*), which can be derived starting from the expression for Hooke’s Law in terms of *E* and the Poisson ratio, *ν*: *ε*_11_ = 1/*E*[*σ*_11_ − *ν*(*σ*_22_ + *σ*_33_)]^65^, and similarly for *ε*_22_ and *ε*_33_. For a cubic system, *ε*_11_ = *S*_11_*σ*_11_ + *S*_12_*σ*_22_ + *S*_12_*σ*_33_ (similarly for *ε*_22_ and *ε*_33_), where *S*_*ij*_ are the elements of the elastic compliance tensor, so that 1/*E* = *S*_11_ and −*ν*/*E* = *S*_12_. For a cubic system, the bulk modulus is *B* = 1/[3(*S*_11_ + 2*S*_12_)] = *E*/[3(1 − 2*ν*)]. The Poisson ratio can be written as *ν* = (3*B* − 2*G*)/(6*B* + 2*G*), and combining with the expression for *B* and rearranging gives the required *E* = 9*BG*/(3*B* + *G*).

The elastic properties for the five-metal compositions are first calculated for each of the 49 configurations generated by AFLOW-POCC. The VRH approximated values of *B* and *G* for these configurations are listed in Supplementary Table 3, along with the AFLOW-POCC ensemble averaged electronic density of states (see Supplementary Figure 39). The average elastic moduli are then obtained, weighted according to the Boltzmann distribution at a temperature of 2200 °C (the experimental sintering temperature). These calculated values are compared with those obtained using a ROM (average of the binary components, weighted according to fractional composition in the sample).

Three different models are used for predicting the Vickers hardness based on the elastic moduli: Chen et al. (*H*_V_ = 2(*k*^2^*G*)^0.585^ − 3; *k* = *G*/*B*)^54^, Teter (*H*_V_ = 0.151*G*)^55^, and Tian et al. (*H*_V_ = 0.92*k*^1.137^*G*^0.708^; *k* = *G*/*B*)^56^. Note, however, that these models are based on the elastic response of the materials, and do not take into account phenomena such as plastic deformation, slip planes, and lattice defects.

### Sample preparation

Initial powders of each of the eight binary precursor carbides (HfC, NbC, TaC, TiC, Mo_2_C, VC, W_2_C, ZrC) are obtained in >99% purity and −325 mesh (<44 μm) particle size (Alfa Aesar). Samples are weighed out in 15 g batches and mixed to achieve the desired five-metal carbide compositions. To ensure adequate mixing, each sample is ball milled in a shaker pot mill for a total of 2 h in individual 30-min intervals intersected by 10-min rest times to avoid heating and consequent oxide formation. All milling is done in tungsten carbide-lined stainless steel milling jars with tungsten carbide grinding media.

Bulk sample pellets are synthesized via solid-state processing routes. The field-assisted sintering technique (FAST), also called SPS, is employed to simultaneously densify and react the compositions into single-phase materials. For all samples, sintering is done at 2200 °C with a heating rate of 100 °C/min, 30 MPa uniaxial pressure, and a 5-min dwell at temperature. Samples are heated in vacuum atmosphere to 1300 °C followed by flowing argon to 2200 °C. All sintering is done in 20 mm graphite die and plunger sets with graphite foil surrounding the samples to prevent reaction with the die.

### Sample analysis

Elemental analysis is performed using an FEI Quanta 600 SEM equipped with a Bruker e-Flash EDS detector at an accelerating voltage of 20 kV. Microstructural scanning electron microscope (SEM) imaging is carried out using an FEI Apreo FE-SEM at an accelerating voltage of 5 kV, with a combination of secondary and back-scattered electron detectors to show phase contrast. Crystal phase analysis is performed using a Rigaku Miniflex X-ray Diffractometer with a stepsize of 0.02° and 5-s dwells, using Cu K*α* radiation (wavelength *λ* = 1.54059 Å) for all measurements and calculation of the lattice parameter. All sample patterns are fitted in Materials Data Incorporated’s (MDI) Jade 9 software^66^ with a residual of fit *R* < 8%. Lattice parameter, *a*_exp_, values of 4.353 Å, 4.500 Å, 4.415 Å, 4.434 Å, 4.355 Å, 4.502 Å, 4.506 Å, 4.534 Å, and 4.476 Å were measured for MoNbTaVWC_5_, HfNbTaTiZrC_5_, HfNbTaTiVC_5_, HfNbTaTiWC_5_, NbTaTiVWC_5_, HfTaTiWZrC_5_, HfMoTaWZrC_5_, HfMoVWZrC_5_, and HfMoTiWZrC_5_, respectively (for multi-phase samples, *a*_exp_ refers to the primary cubic phase).

For analysis of sample peak broadening *β*_S_, instrumental broadening *β*_I_ must first be determined. For this, a NIST 660b LaB_6_ standard is run under the same conditions as each carbide sample. The instrumental profile is then fitted, and *β*_I_ is determined to vary with Bragg angle *θ* as:

5 $$\beta _{\mathrm{I}} = 0.1750985 - 0.001560626\theta + 0.00001125342\theta ^2.$$

*β*_S_ is determined by subtracting *β*_I_ from the measured broadening *β*_M_: $\beta _{\mathrm{S}}^x = \beta _{\mathrm{M}}^x - \beta _I^x$. *β*_M_ is measured as a function of *θ*, and *x* is a constant between 1.0 and 2.0. In the current analysis, *x* is set to 2.0 due to the Gaussian-like shape of the instrument peaks, as this value leads to the lowest standard deviation of linear fits to the peak broadening data.

Both crystallite size and lattice strain contribute to *β*_S_^49,67,68:^

6 $$\beta _{\mathrm{S}} = 4\varepsilon {\mathrm{tan}}\theta + \frac{{K\lambda }}{{D{\mathrm{cos}}\theta }},$$

where *ε* is the lattice strain or variation in interplanar spacing due to chemical inhomogeneity, *K* is a constant (dependent on the grain shape), *λ* is the incident X-ray wavelength and *D* is the crystallite size. Rearranging Eq. 6 gives:

7 $$\beta _{\mathrm{S}}{\mathrm{cos}}\theta = 4\varepsilon {\mathrm{sin}}\theta + \frac{{K\lambda }}{D}.$$

The slope of a linear fit to the plot of *β*_S_cos*θ* against sin*θ* is equal to the strain, or lattice distortion, whereas the *y*-intercept of a linear fit with zero slope determines the crystallite size.

### Mechanical testing

Mechanical properties of each of the single-phase compositions are tested using a Keysight NanoIndenter G200 with a Berkovich indenter tip. To rule out indentation size effects, testing is carried out at loads of both 50 mN and 300 mN, and no significant deviation in hardness or modulus is observed. To allow valid cross-comparison, each of the high entropy carbides is compared with the binary carbides, which were hot-pressed and indentation tested under identical conditions. For the reported values, tests are carried out according to the standard method outlined in ISO 14577 using a maximum load of 50 mN. Values are calculated as an average of 40 indents, and are reported with errors of plus or minus one standard deviation. A fused crystal silica standard is run prior to each test to ensure proper equipment calibration is maintained. Samples are polycrystalline with grain sizes between 10 μm and 30 μm. Prior to indentation testing each sample is vibratory polished using 0.05 μm colloidal silica for 12 h to ensure minimal surface roughness. All tests are carried out at a temperature of 27 °C ± 0.5 °C. Indentation data are analyzed according to the methods of Oliver and Pharr^69,70^. The elastic (i.e., Young’s) modulus is determined using $1/E_{{\mathrm{eff}}} = (1 - \nu ^2)/E + (1 - \nu _{\mathrm{I}}^2)/E_{\mathrm{I}}$, where *E*_eff_ is the effective modulus (sometimes called the reduced modulus) obtained from nanoindentation, *E* and *ν* are the Young’s modulus and Poisson’s ratio, respectively, for the specimen, whereas *E*_I_ and *ν*_I_ are the same parameters for the indenter. A Poisson’s ratio for each of the binary carbides is obtained from literature^71^ where available. For five-metal carbide samples where data for each of the constituents is available, the value used for Poisson’s ratio is taken as the average of the constituent binaries. If this average is unavailable (i.e., when Mo and/or W are present), Poisson’s ratio is assumed to be equal to 0.18.

## Electronic supplementary material

Supplementary Information

## Data Availability

All the ab-initio data are freely available to the public as part of the AFLOW online repository and can be accessed through AFLOW.org following the REST-API interface^59^ and AFLUX search language^76^.
