# Supplementary material for: High-entropy high-hardness metal carbides discovered by entropy descriptors
Source: Nat Commun. 2018 Nov 26;9:4980. doi: 10.1038/s41467-018-07160-7 (PMC6255778; doi:10.1038/s41467-018-07160-7)
Supplement: Supplementary file 1 — Supplementary Information [file 41467_2018_7160_MOESM1_ESM.pdf]

SUPPLEMENTARY INFORMATION
High-entropy high-hardness metal carbides discovered by entropy descriptors

Pranab Sarker,[1,*] Tyler Harrington,[2,*] Cormac Toher,[1] Corey Oses,[1] Mojtaba Samiee,[2]
Jon-Paul Maria,[3] Donald W. Brenner,[3] Kenneth S. Vecchio,[2,4,†] and Stefano Curtarolo[5,6,‡]

[1] *Department of Mechanical Engineering and Materials Science, Duke University, Durham, NC 27708, USA*
[2] *Materials Science and Engineering Program, University of California, San Diego, La Jolla, CA 92093, USA*
[3] *Department of Materials Science and Engineering, North Carolina State University, Raleigh, NC 27695, USA*
[4] *Department of NanoEngineering, University of California, San Diego, La Jolla, CA 92093, USA*
[5] *Materials Science, Electrical Engineering, Physics and Chemistry, Duke University, Durham NC, 27708, USA*
[6] *Fritz-Haber-Institut der Max-Planck-Gesellschaft, 14195 Berlin-Dahlem, Germany*

(Dated: October 16, 2018)

SUPPLEMENTARY NOTE 1: CONTENT

The supplementary information includes: **i.** the binary and ternary convex hull phase diagrams for all component carbide systems; **ii.** the energetic distance from the convex hull, ΔH_{f}, for the lowest energy configuration and the corresponding decomposition reaction products for all 56 5-metal carbide compositions; **iii.** a comparison of XRD spectra for $MoNbTaVWC_{5-x}$ synthesized using both WC and W_2C precursors; **iv.** the elastic response during the mechanical testing for $HfNbTaTiZrC_5$; **v.** a comparison between the Vickers hardness results obtained from calculation, experiment, and rule of mixtures for the 6 single-phase 5-metal carbides; **vi.** the bulk and shear moduli for each configuration of the 6 single-phase 5-metal carbides; **vii.** the electronic density of states for the 9 synthesized 5-metal carbides; **viii.** the formation enthalpies for each configuration of all 56 5-metal carbides; and **ix.** the atomic geometry for all configurations used to calculate the EFA for $HfNbTaTiZrC_5$.

SUPPLEMENTARY NOTE 2: COMPETING ORDERED PHASES

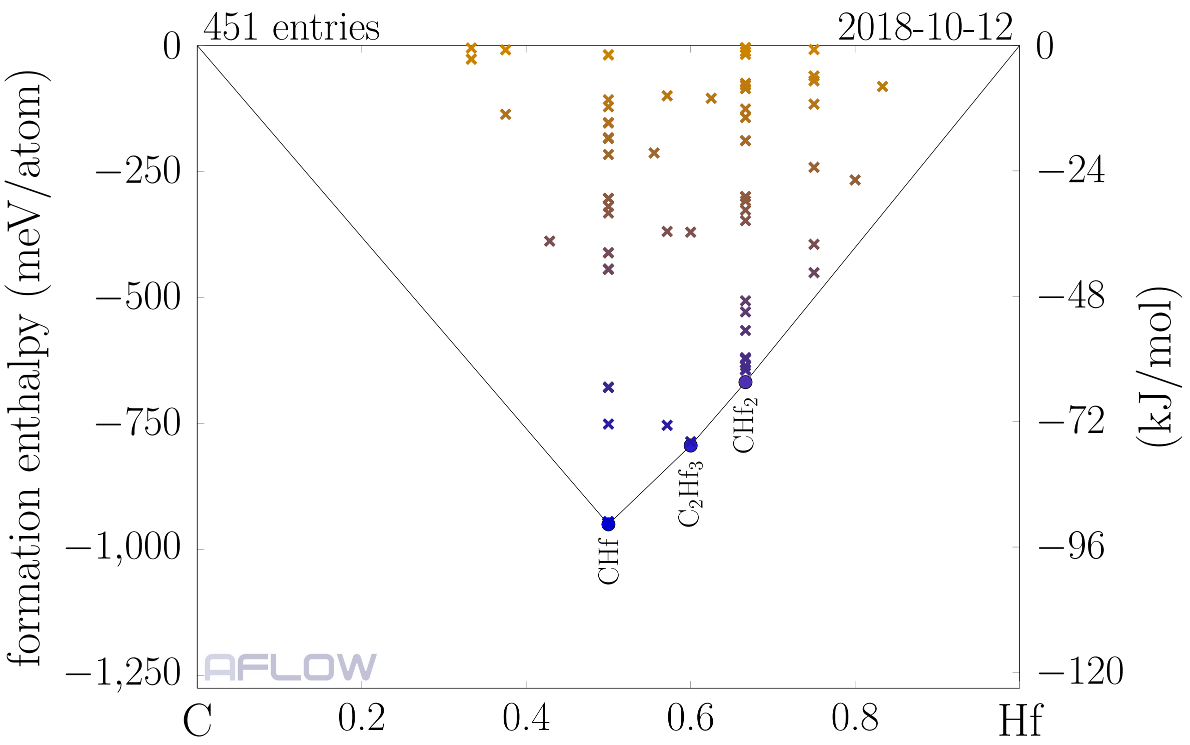

Supplementary Figure 1. CHf binary hull as calculated with AFLOW-CHULL.

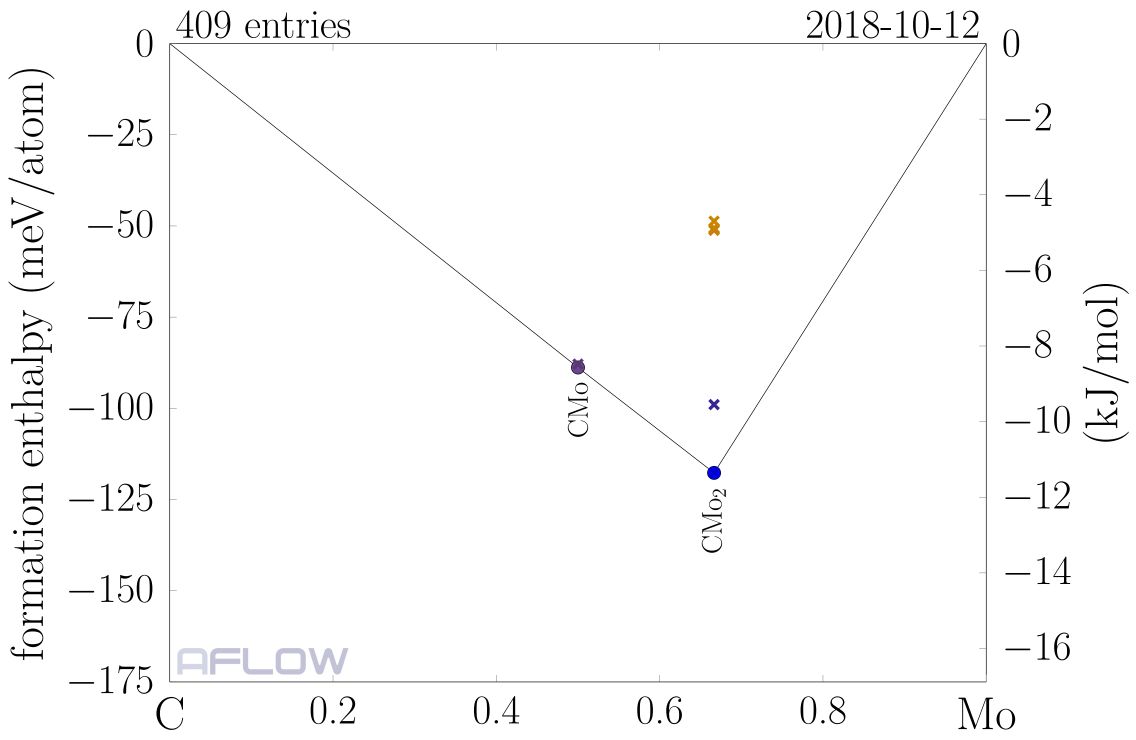

Supplementary Figure 2. CMo binary hull as calculated with AFLOW-CHULL.

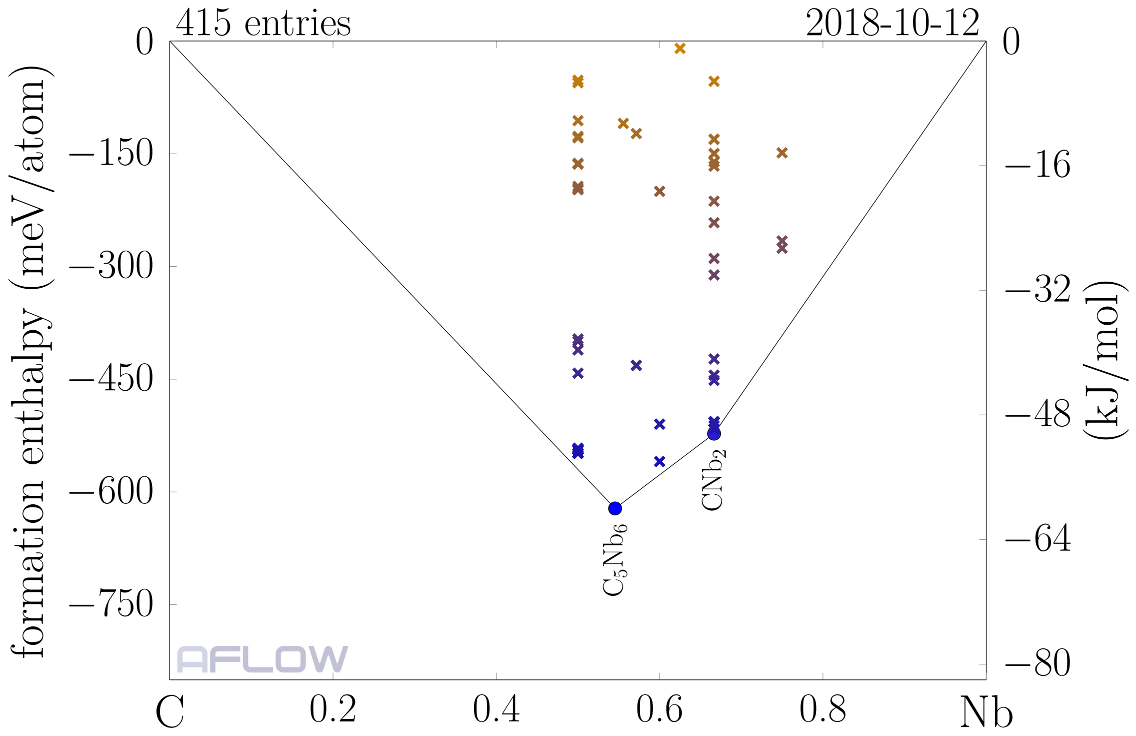

Supplementary Figure 3. CNb binary hull as calculated with AFLOW-CHULL.

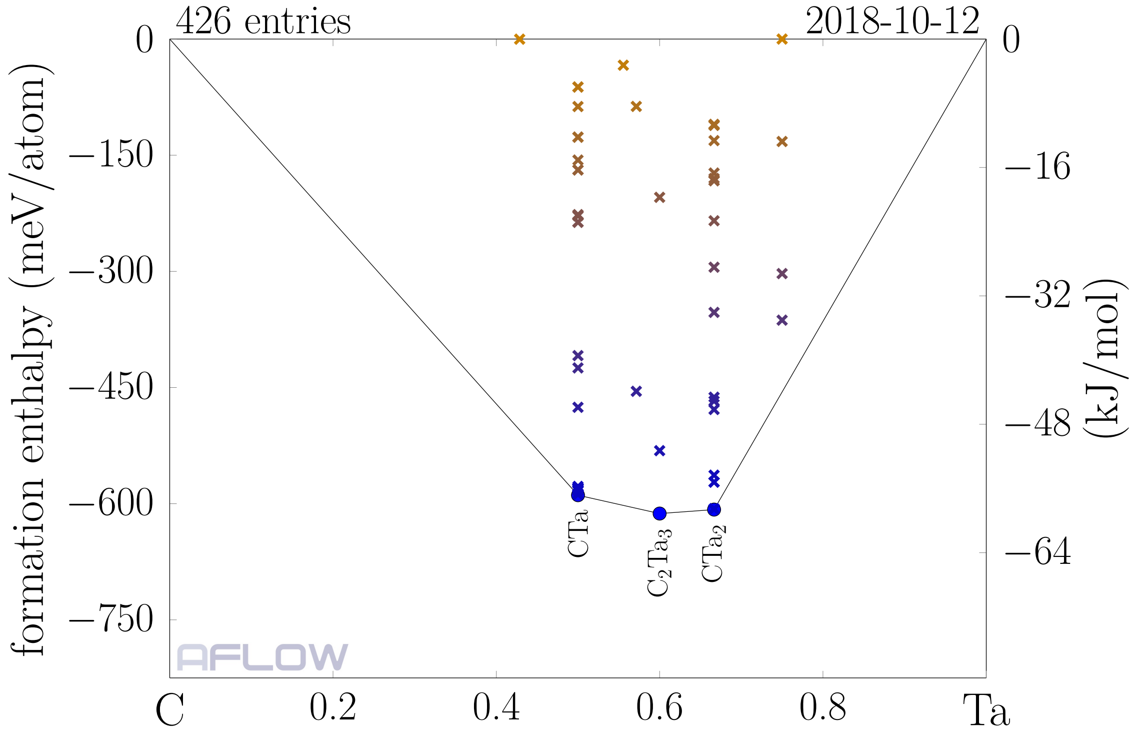

Supplementary Figure 4. CTa binary hull as calculated with AFLOW-CHULL.

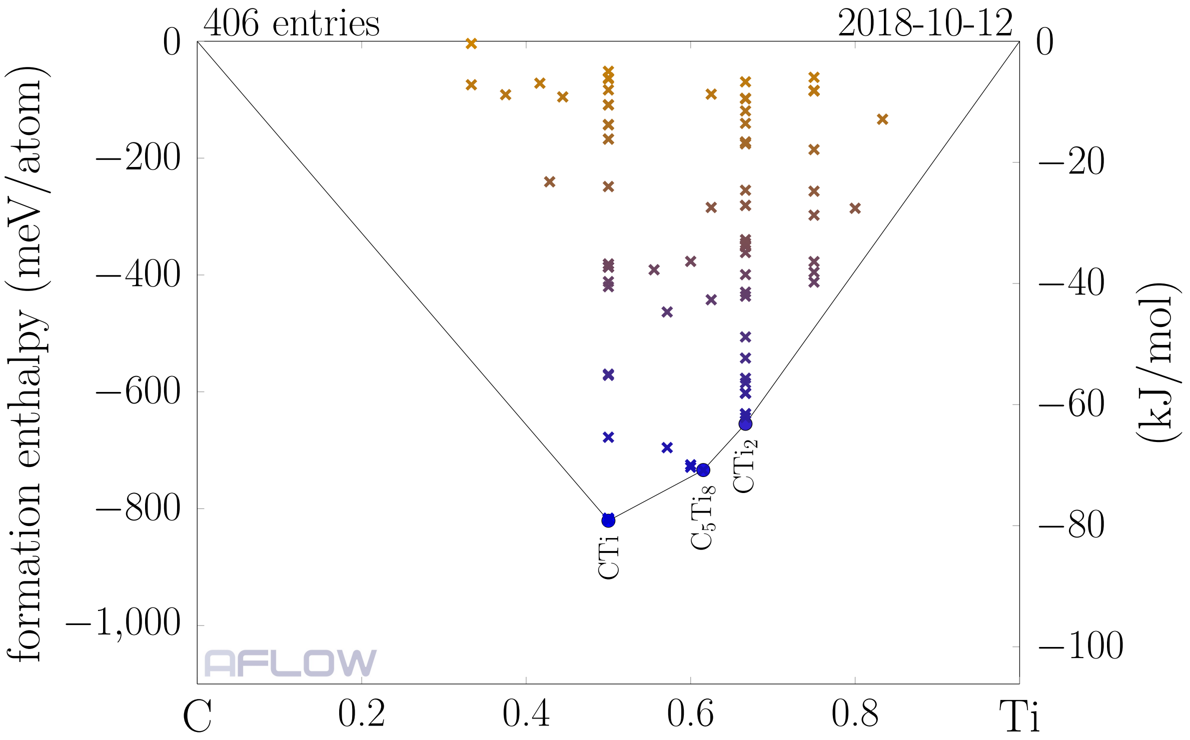

Supplementary Figure 5. CTi binary hull as calculated with AFLOW-CHULL.

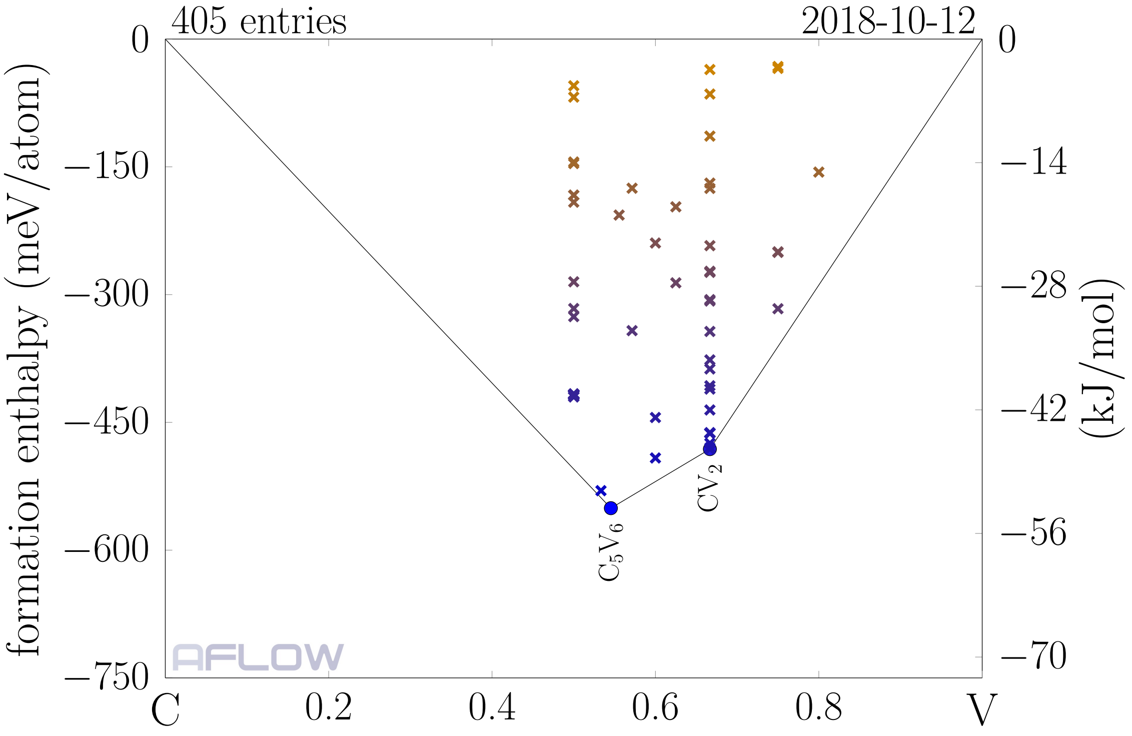

Supplementary Figure 6. CV binary hull as calculated with AFLOW-CHULL.

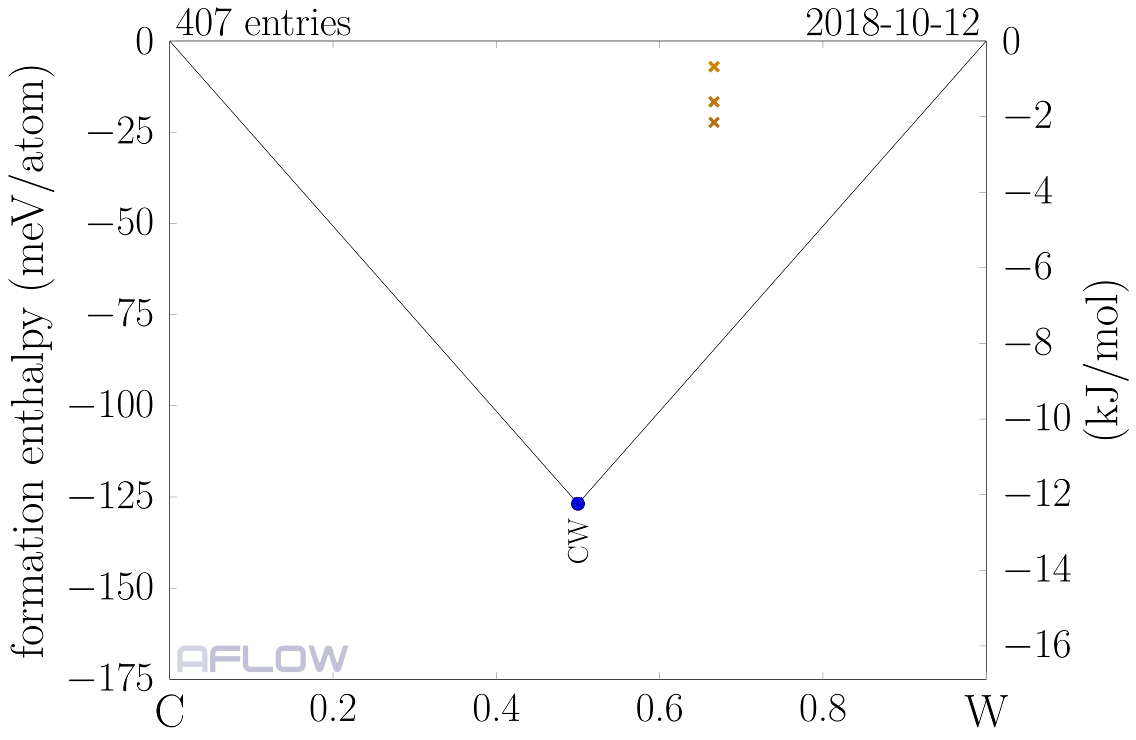

Supplementary Figure 7. CW binary hull as calculated with AFLOW-CHULL.

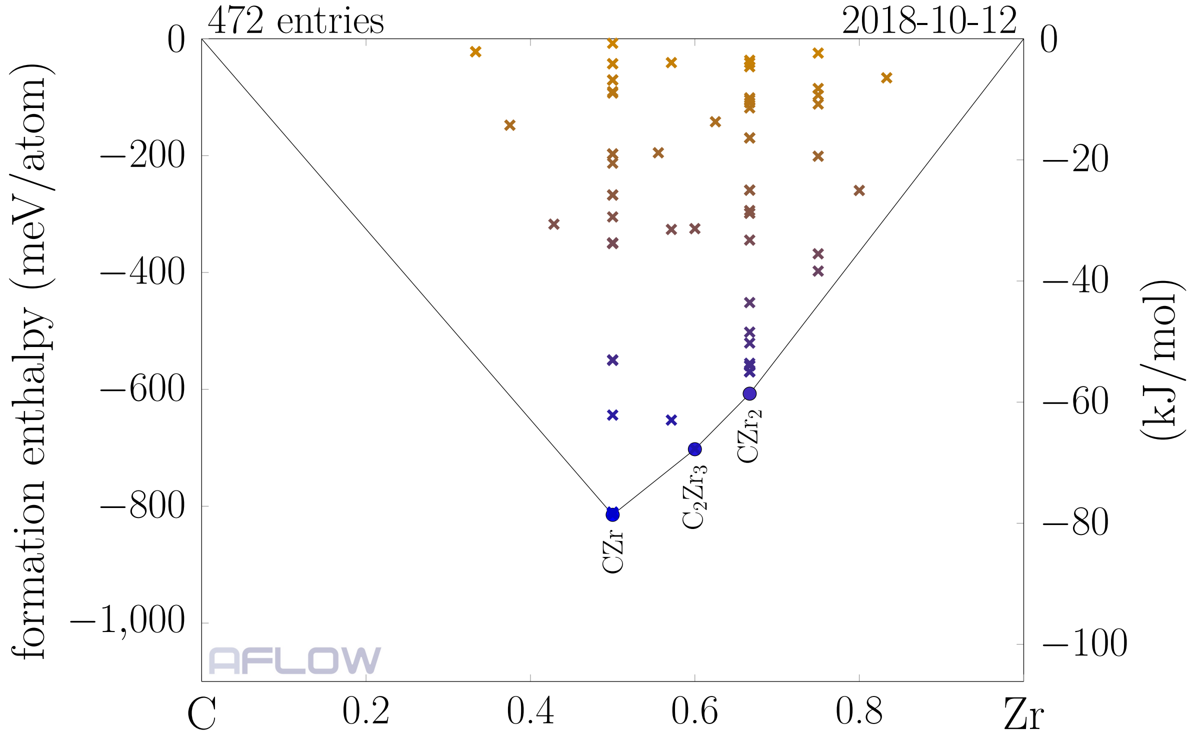

Supplementary Figure 8. CZr binary hull as calculated with AFLOW-CHULL.

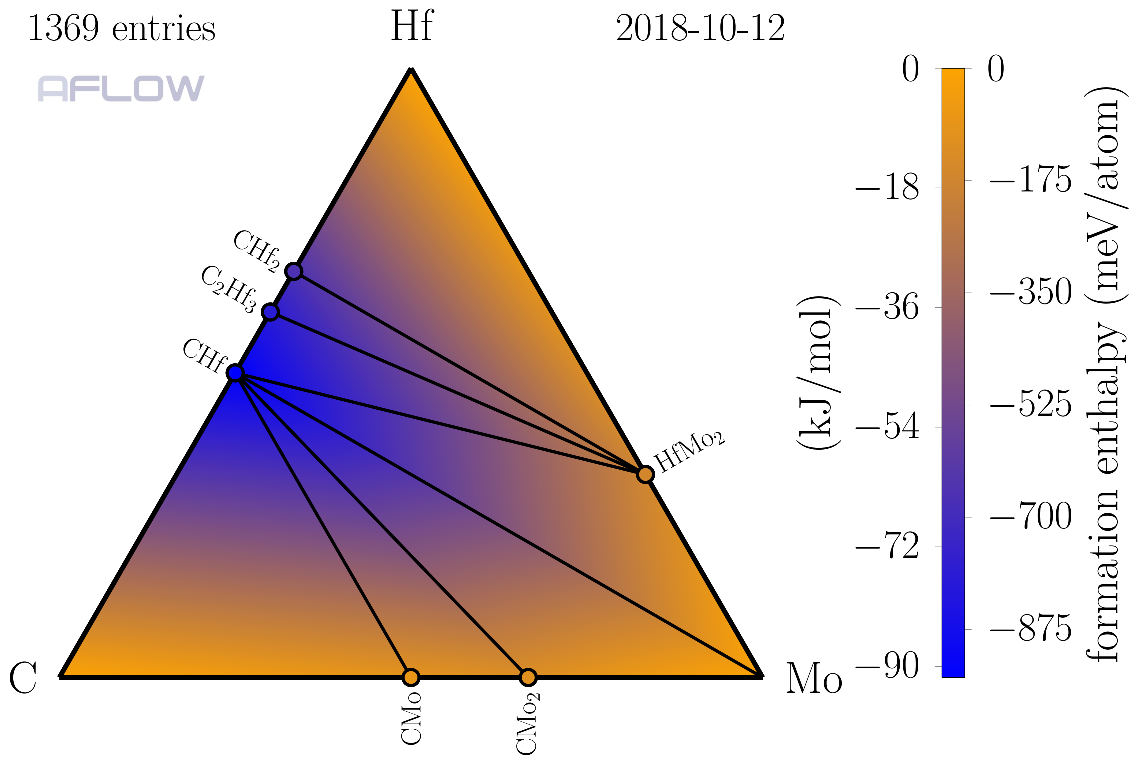

Supplementary Figure 9. CHfMo ternary hull as calculated with AFLOW-CHULL.

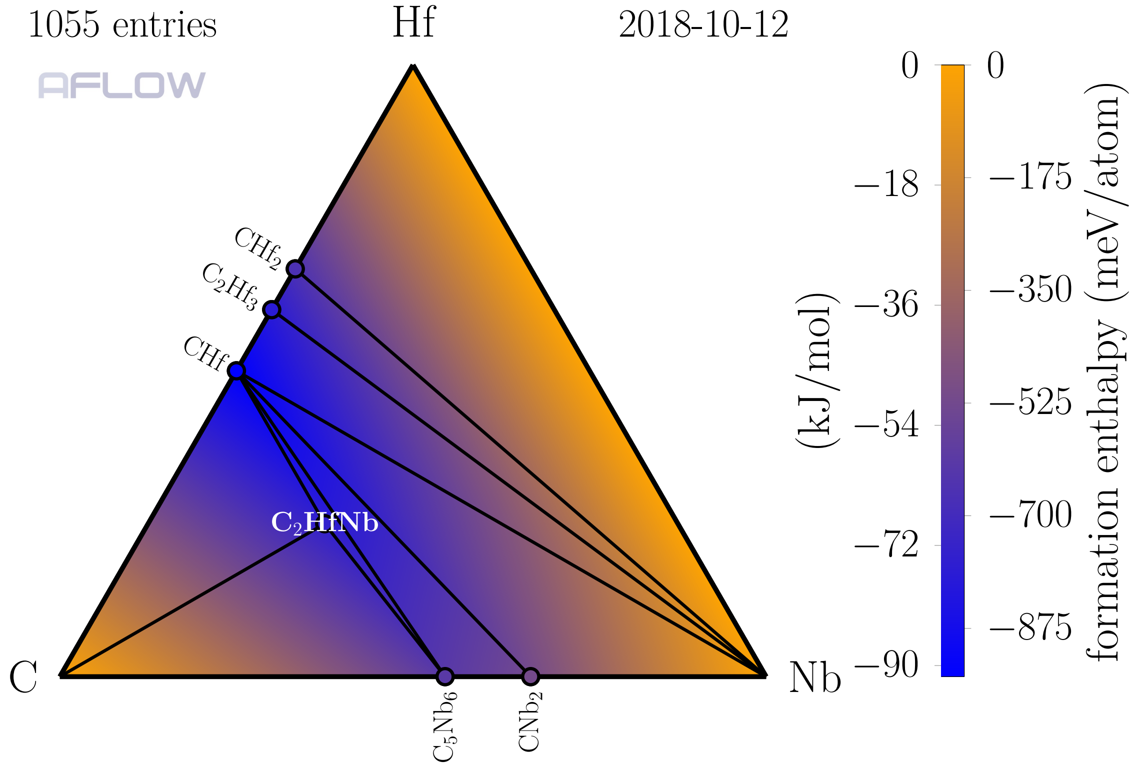

Supplementary Figure 10. CHfNb ternary hull as calculated with AFLOW-CHULL.

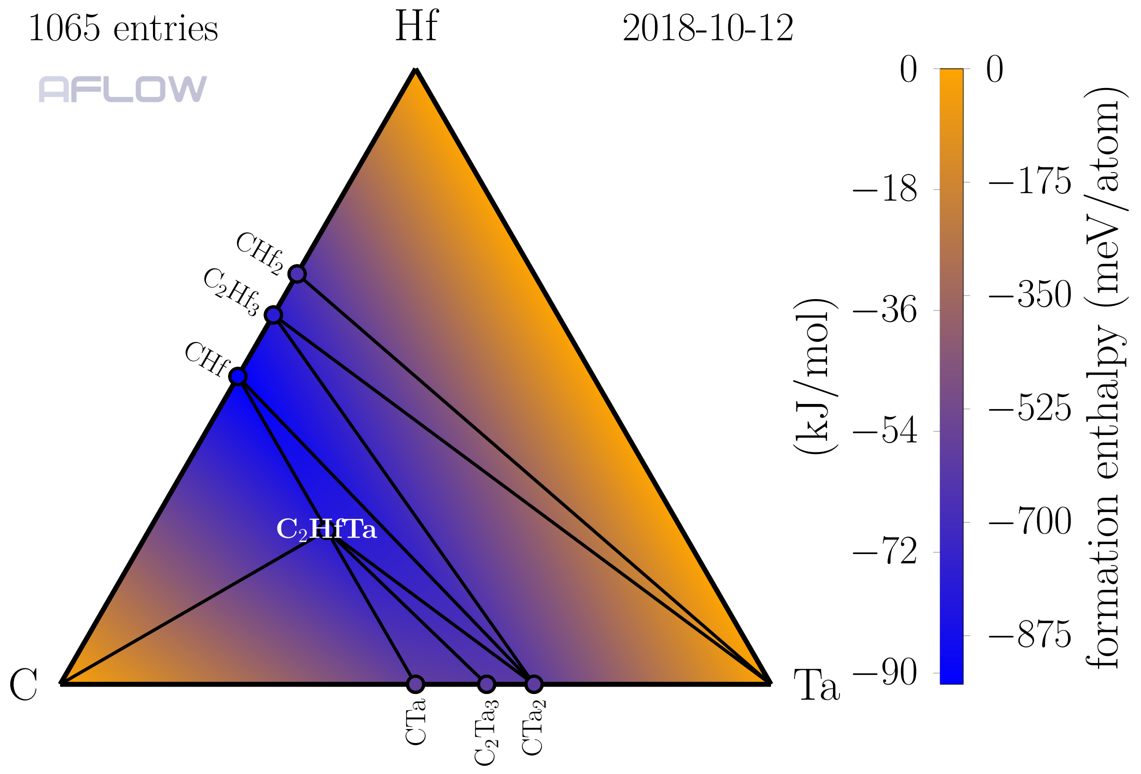

Supplementary Figure 11. CHfTa ternary hull as calculated with AFLOW-CHULL.

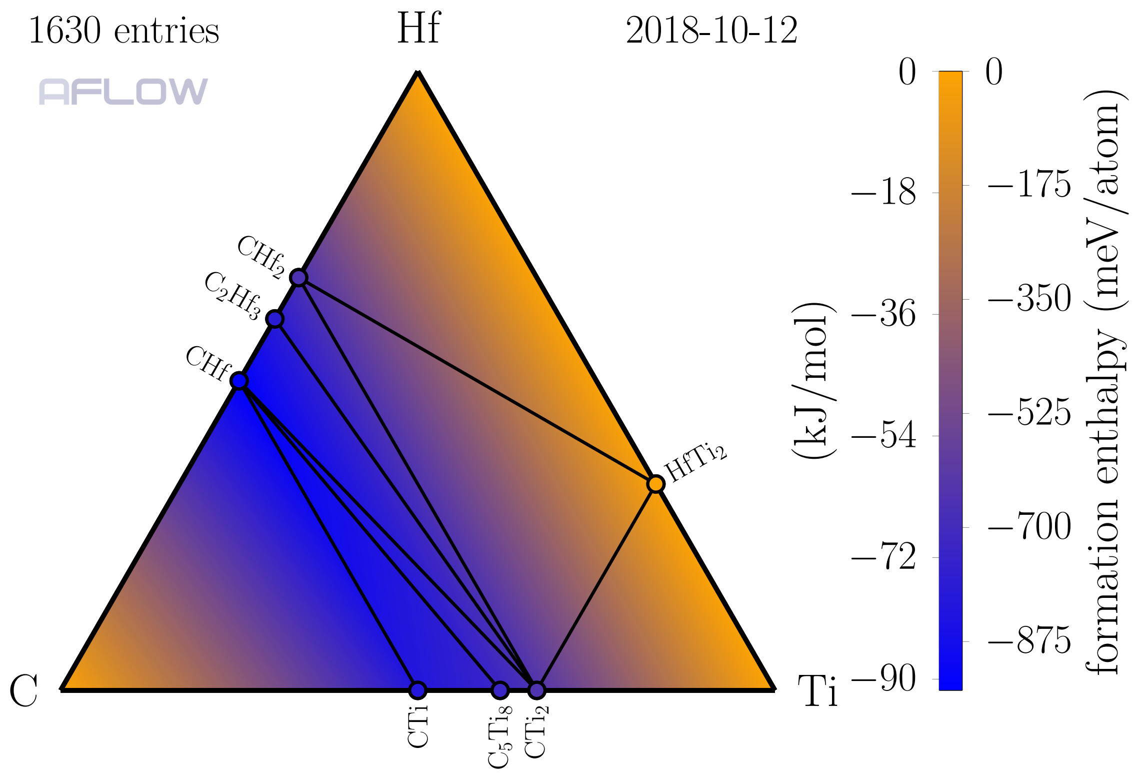

Supplementary Figure 12. CHfTi ternary hull as calculated with AFLOW-CHULL.

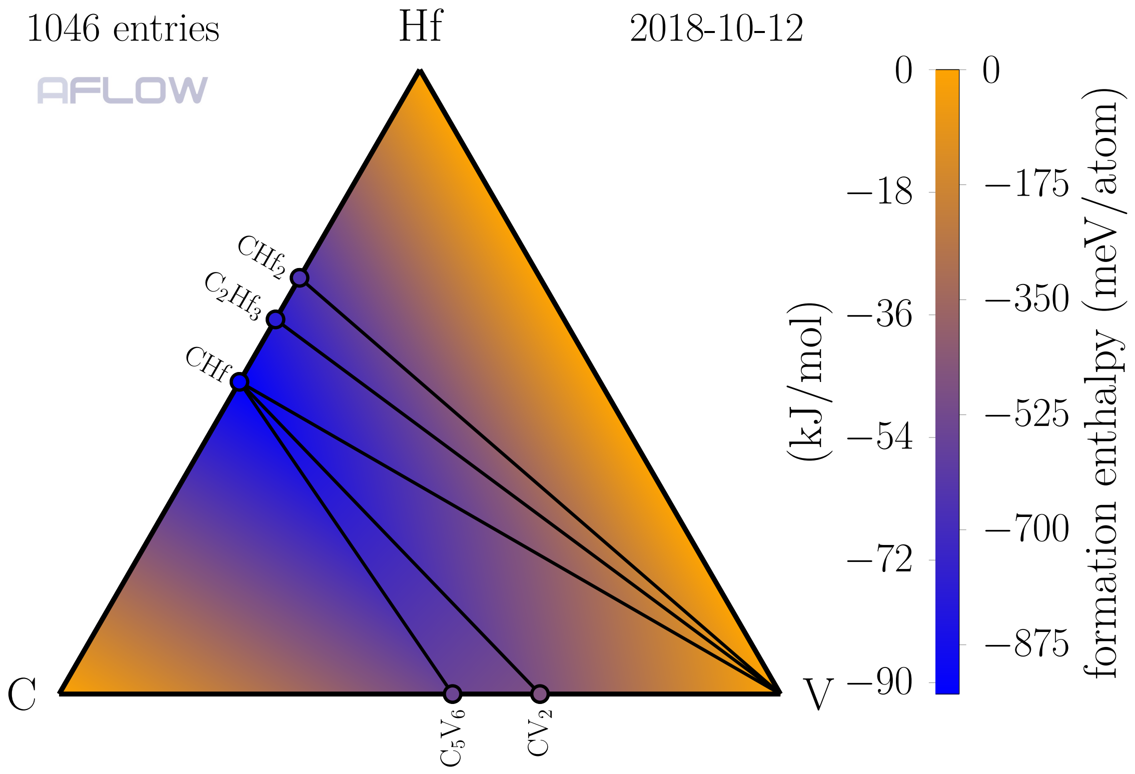

Supplementary Figure 13. CHfV ternary hull as calculated with AFLOW-CHULL.

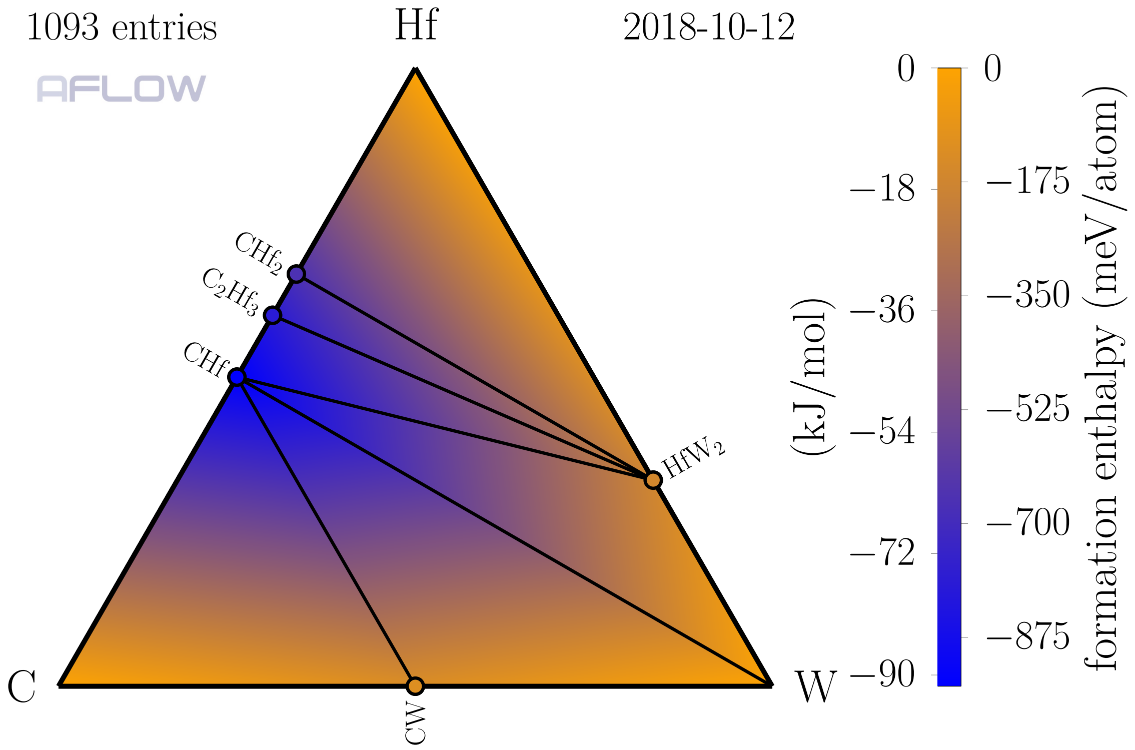

Supplementary Figure 14. CHfW ternary hull as calculated with AFLOW-CHULL.

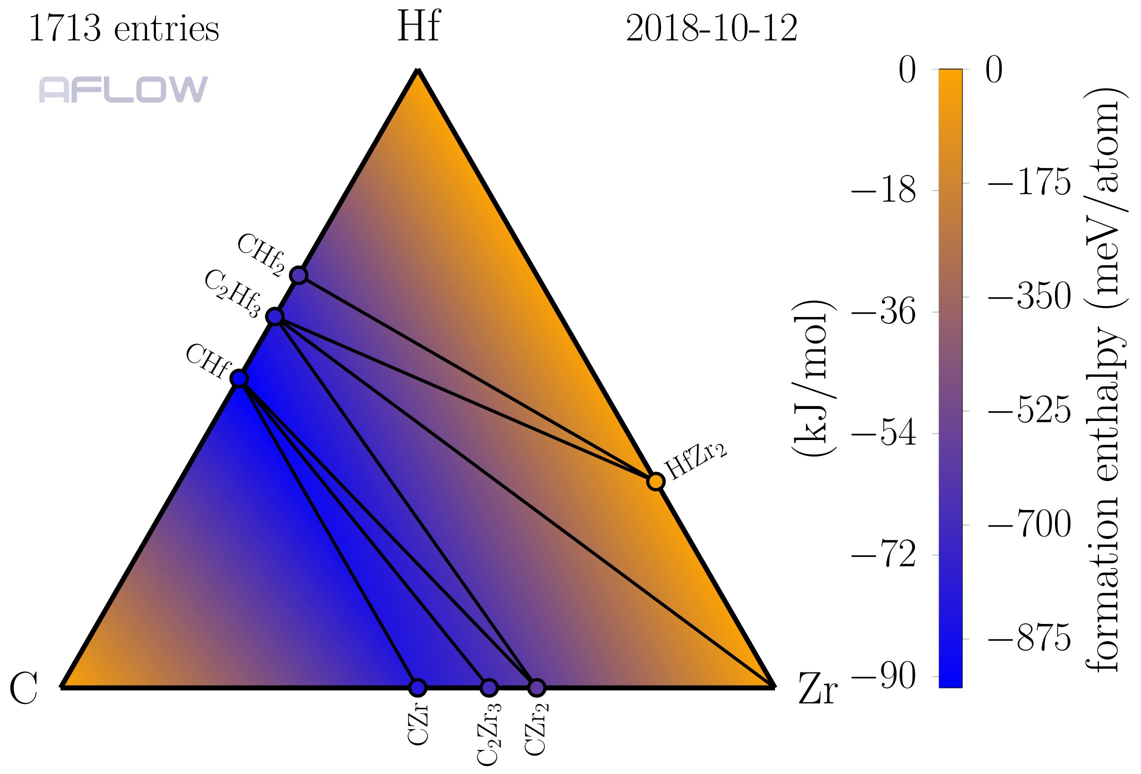

Supplementary Figure 15. CHfZr ternary hull as calculated with AFLOW-CHULL.

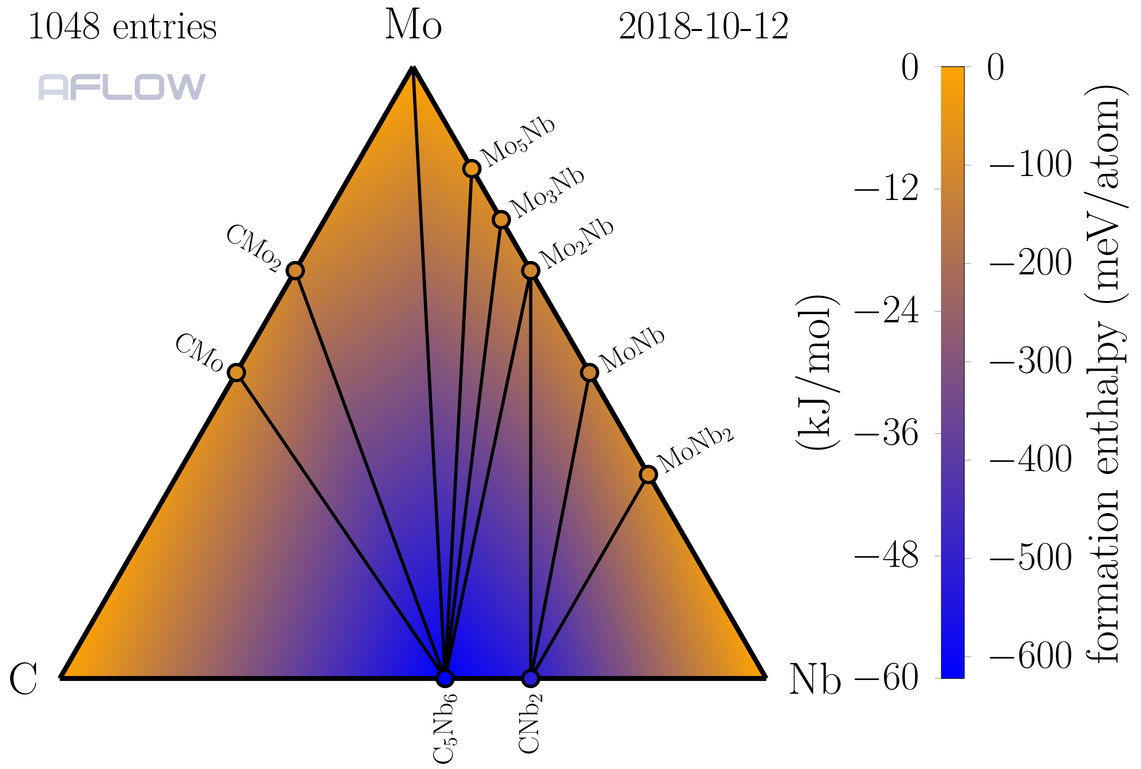

Supplementary Figure 16. CMoNb ternary hull as calculated with AFLOW-CHULL.

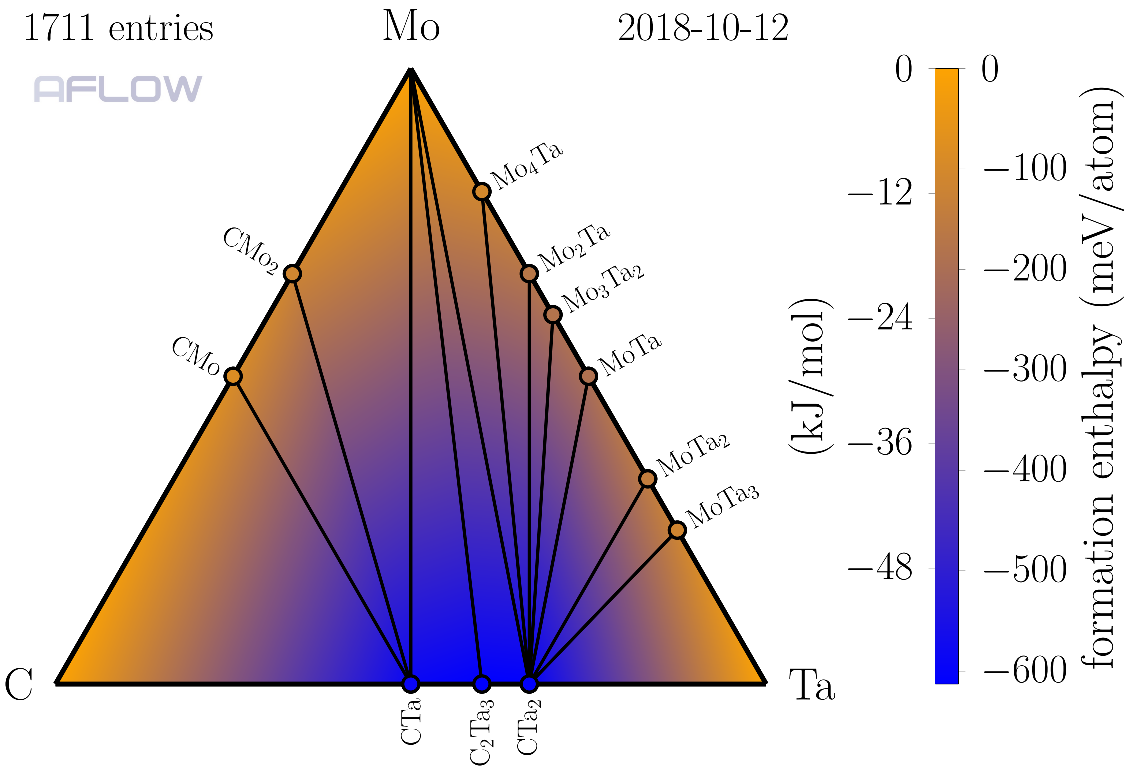

Supplementary Figure 17. CMoTa ternary hull as calculated with AFLOW-CHULL.

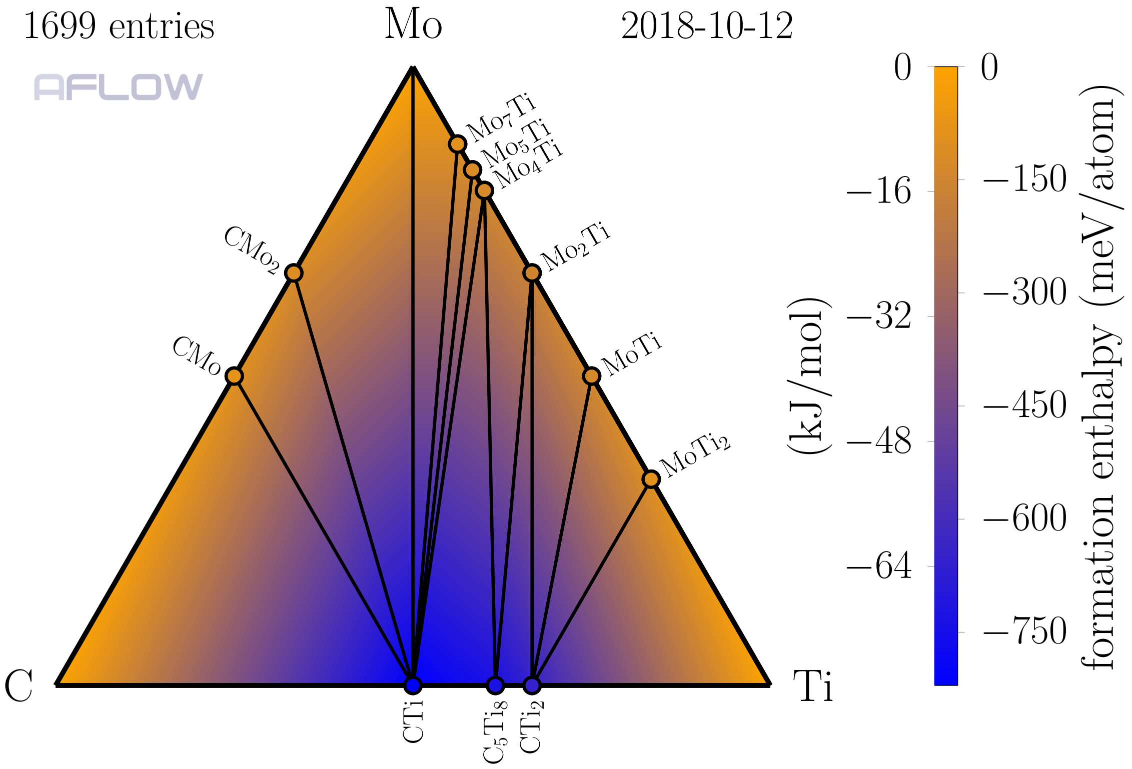

Supplementary Figure 18. CMoTi ternary hull as calculated with AFLOW-CHULL.

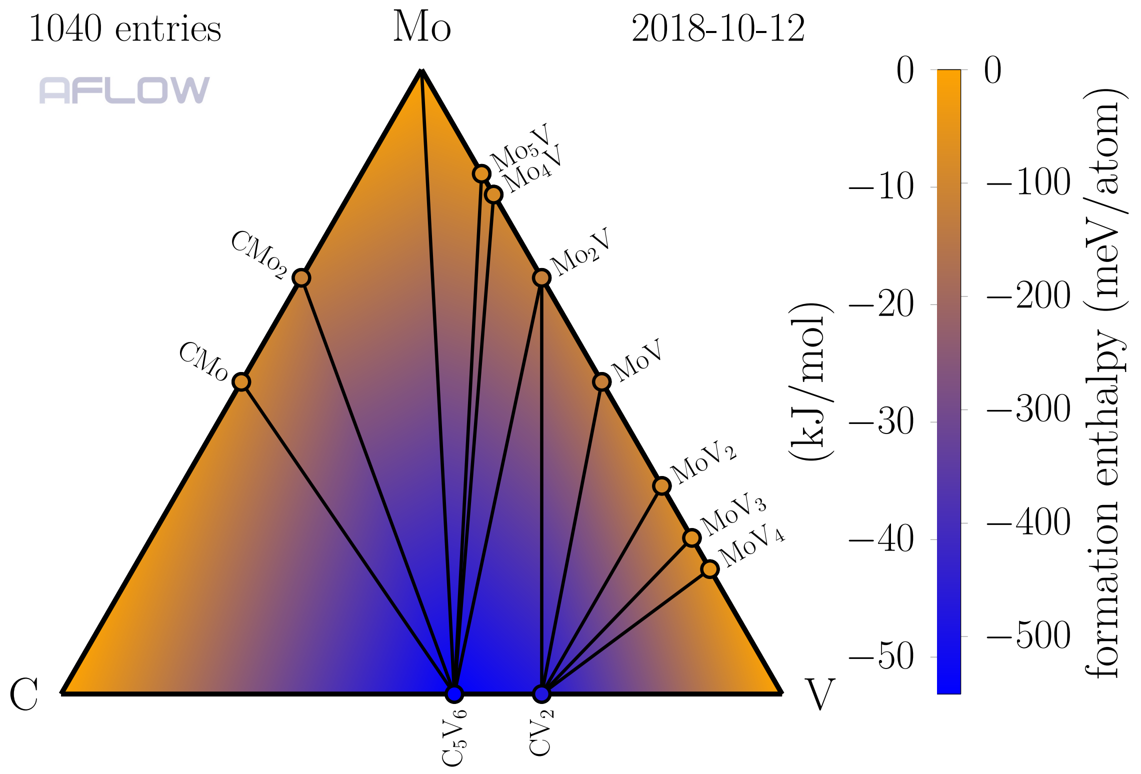

Supplementary Figure 19. CMoV ternary hull as calculated with AFLOW-CHULL.

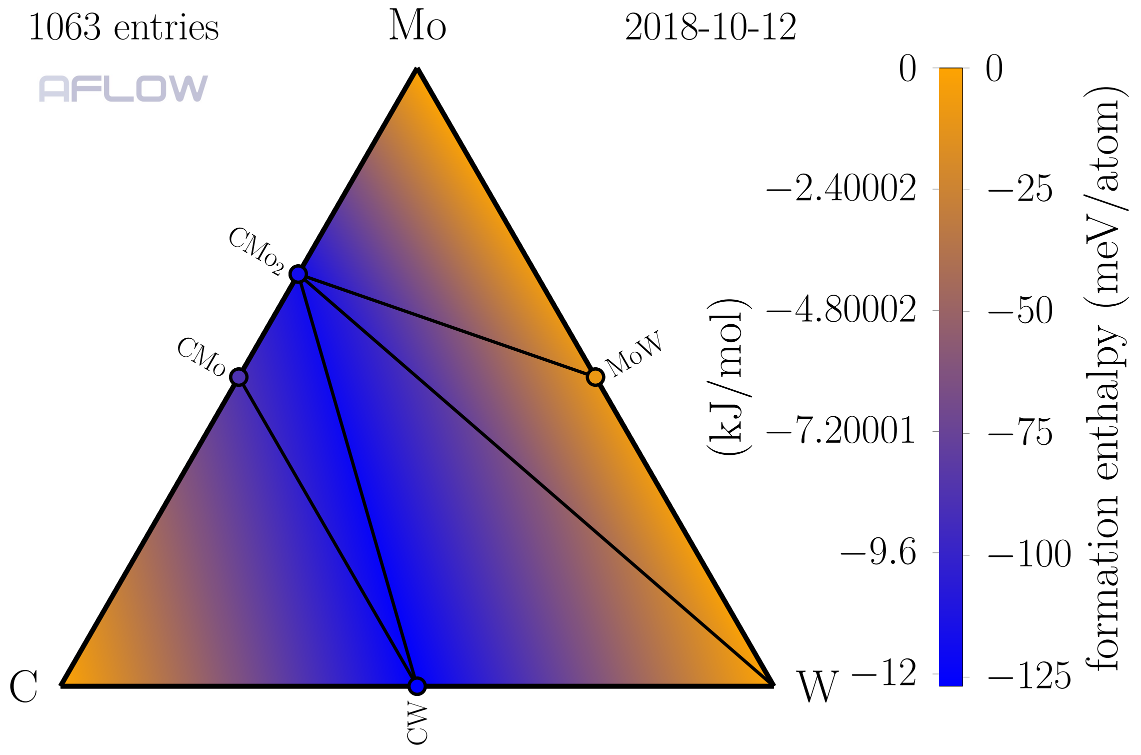

Supplementary Figure 20. CMoW ternary hull as calculated with AFLOW-CHULL.

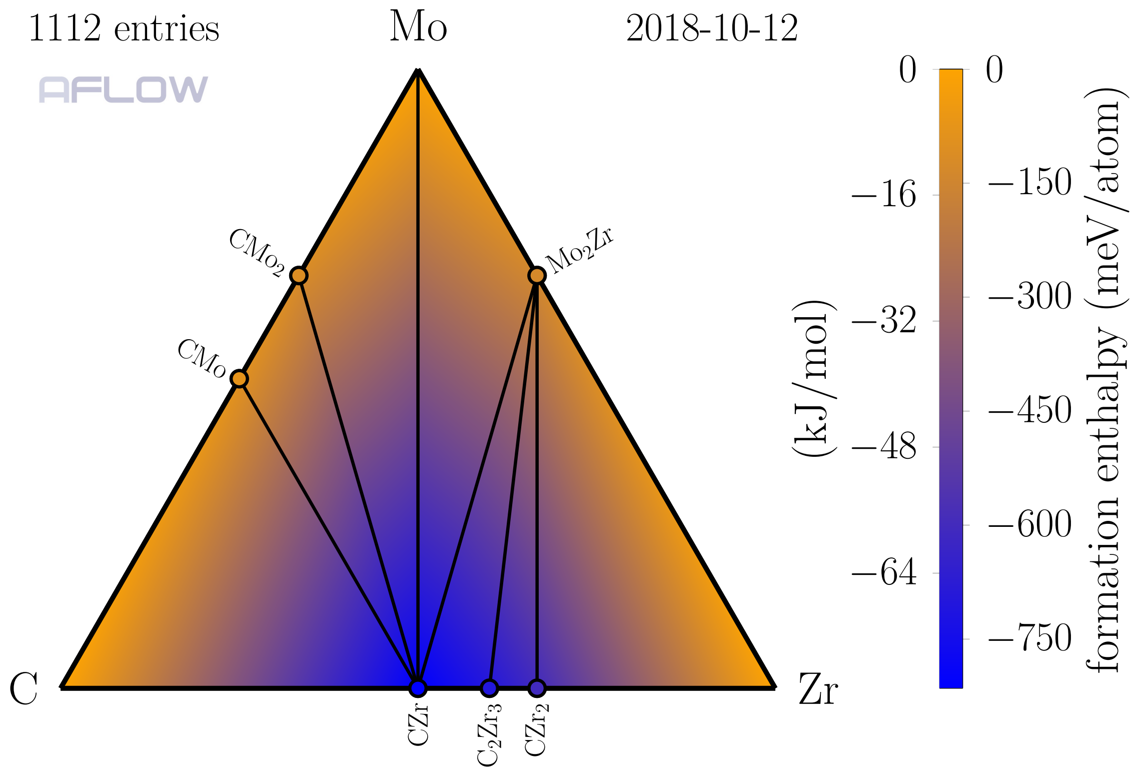

Supplementary Figure 21. CMoZr ternary hull as calculated with AFLOW-CHULL.

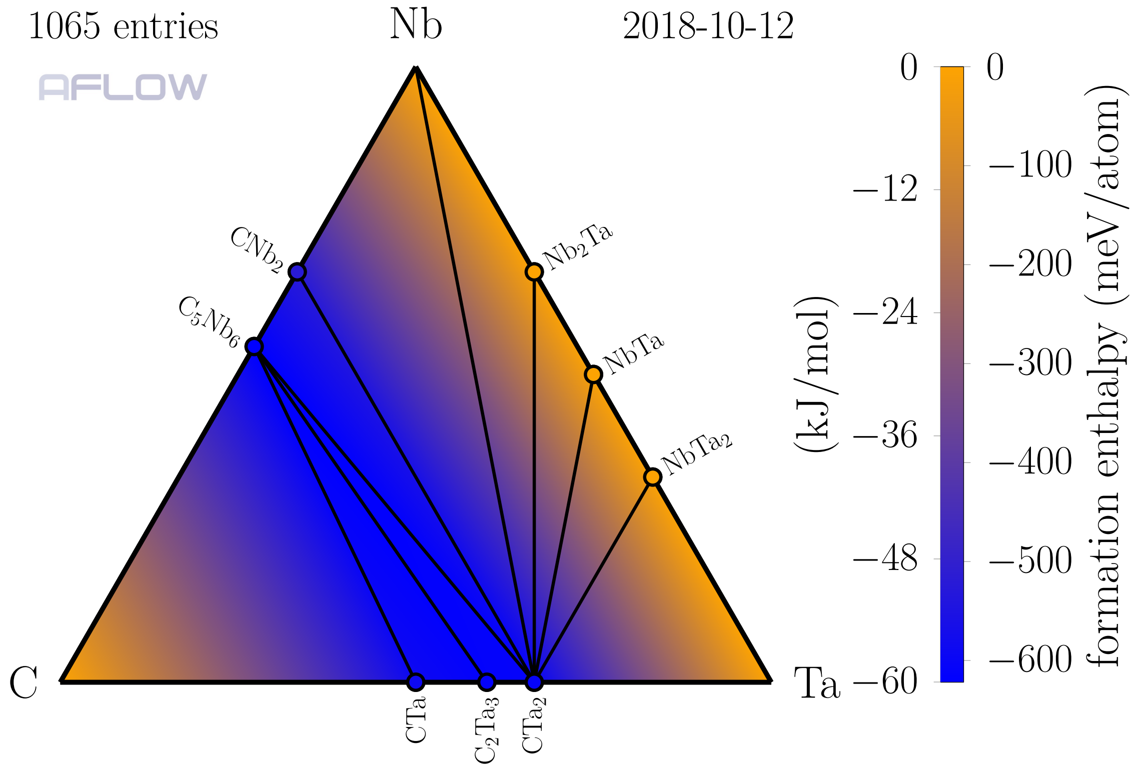

Supplementary Figure 22. CNbTa ternary hull as calculated with AFLOW-CHULL.

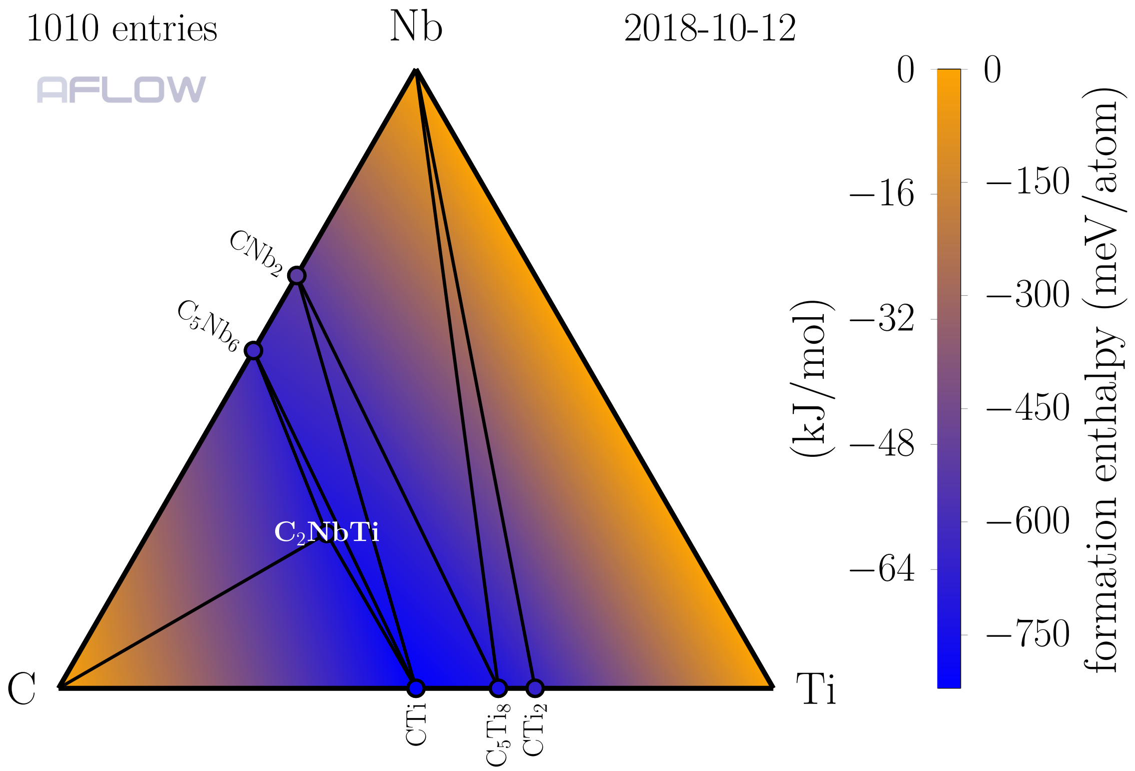

Supplementary Figure 23. CNbTi ternary hull as calculated with AFLOW-CHULL.

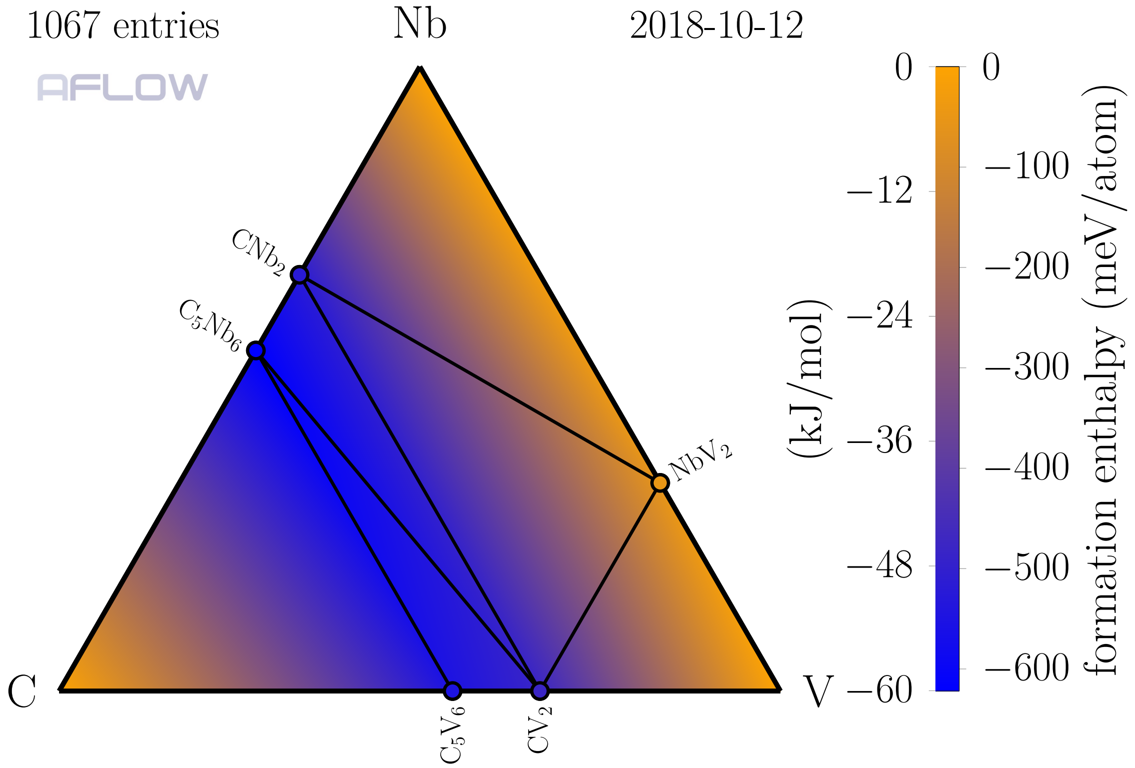

Supplementary Figure 24. CNbV ternary hull as calculated with AFLOW-CHULL.

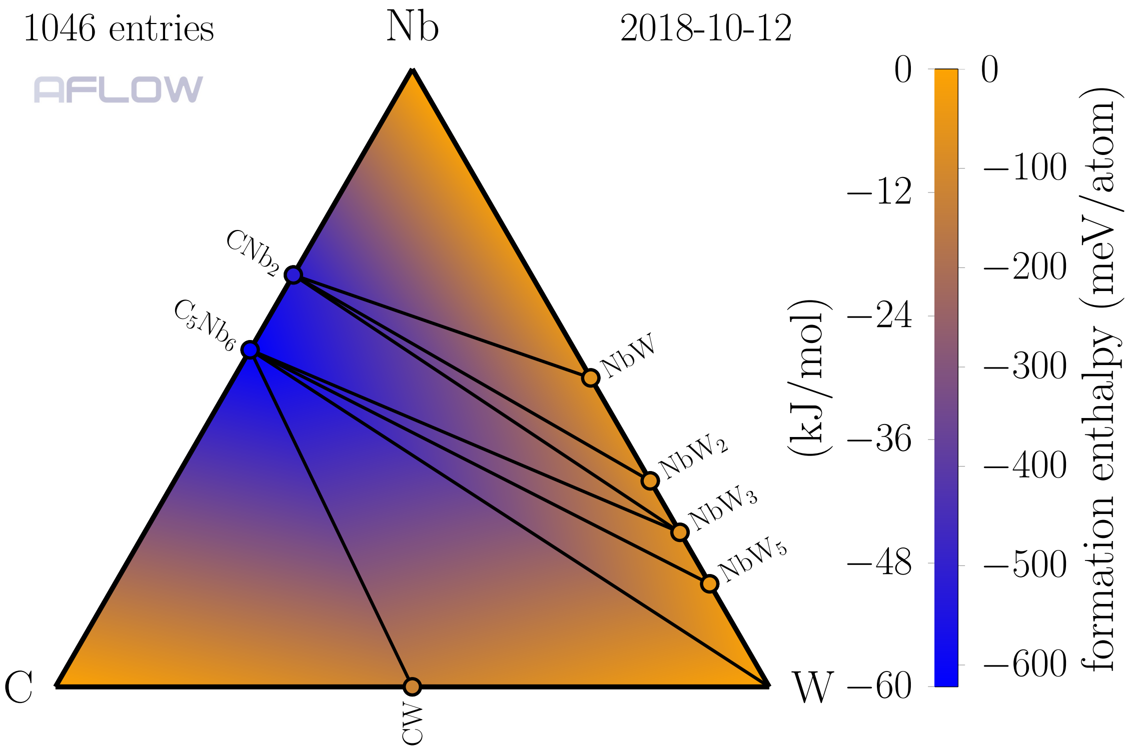

Supplementary Figure 25. CNbW ternary hull as calculated with AFLOW-CHULL.

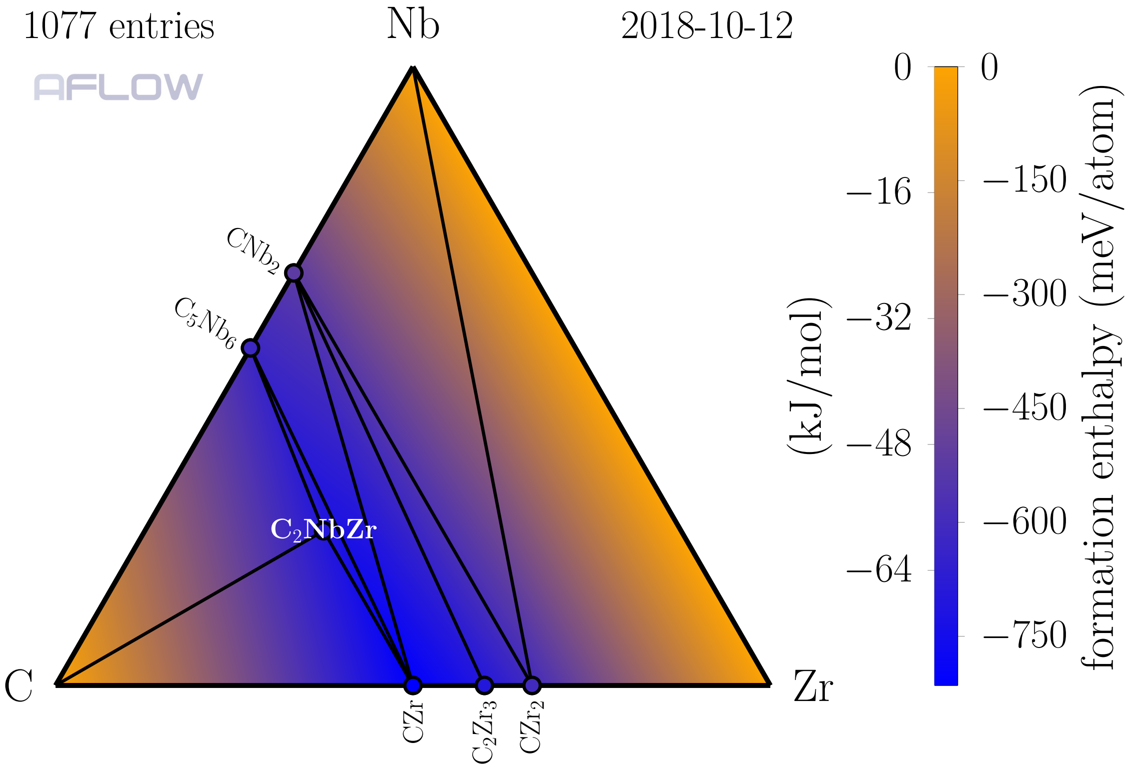

Supplementary Figure 26. CNbZr ternary hull as calculated with AFLOW-CHULL.

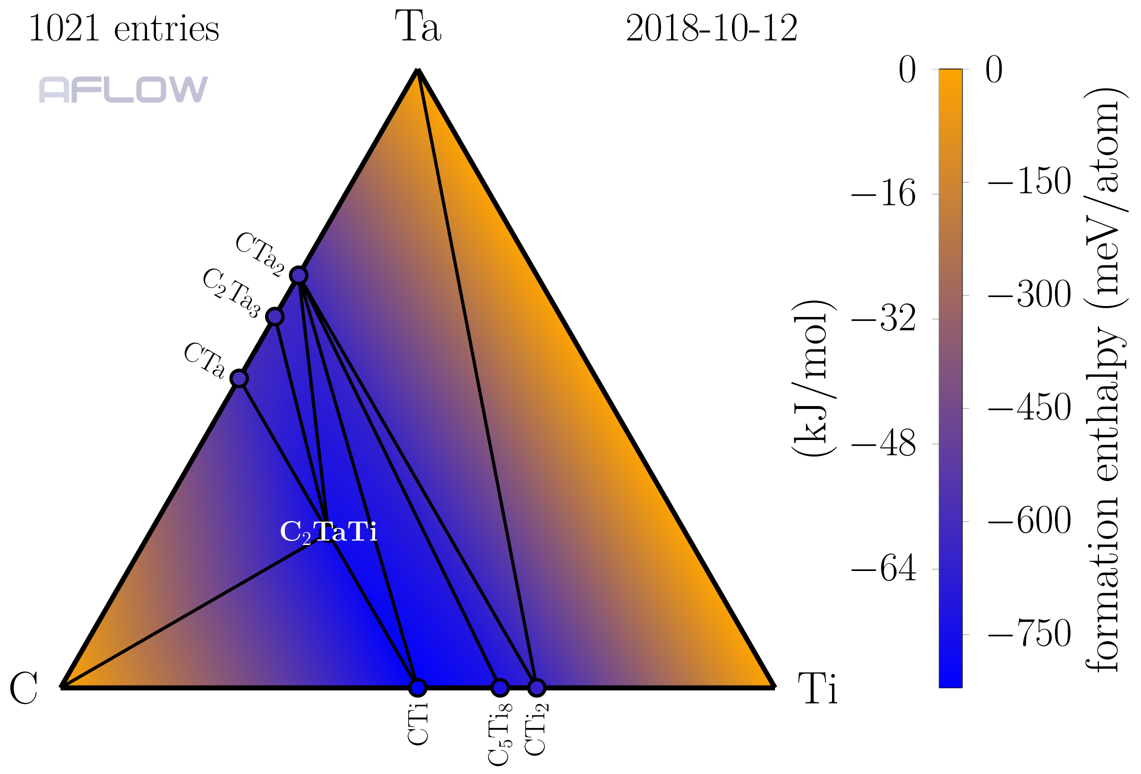

Supplementary Figure 27. CTaTi ternary hull as calculated with AFLOW-CHULL.

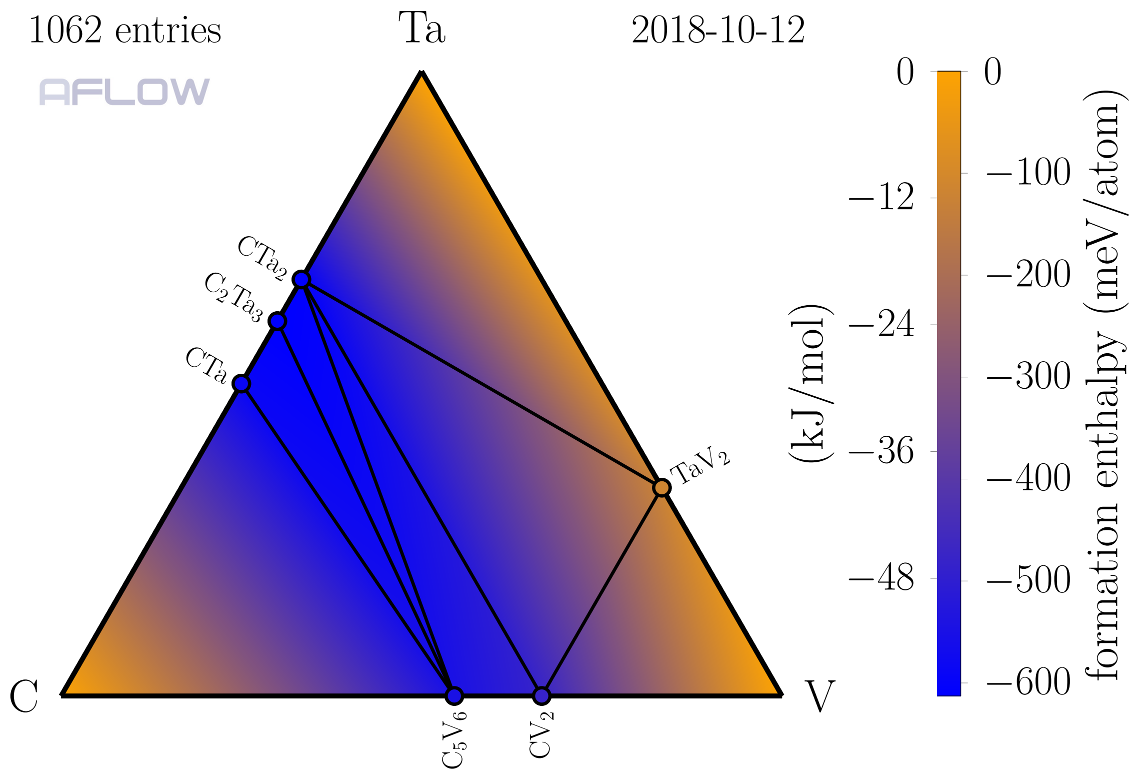

Supplementary Figure 28. CTaV ternary hull as calculated with AFLOW-CHULL.

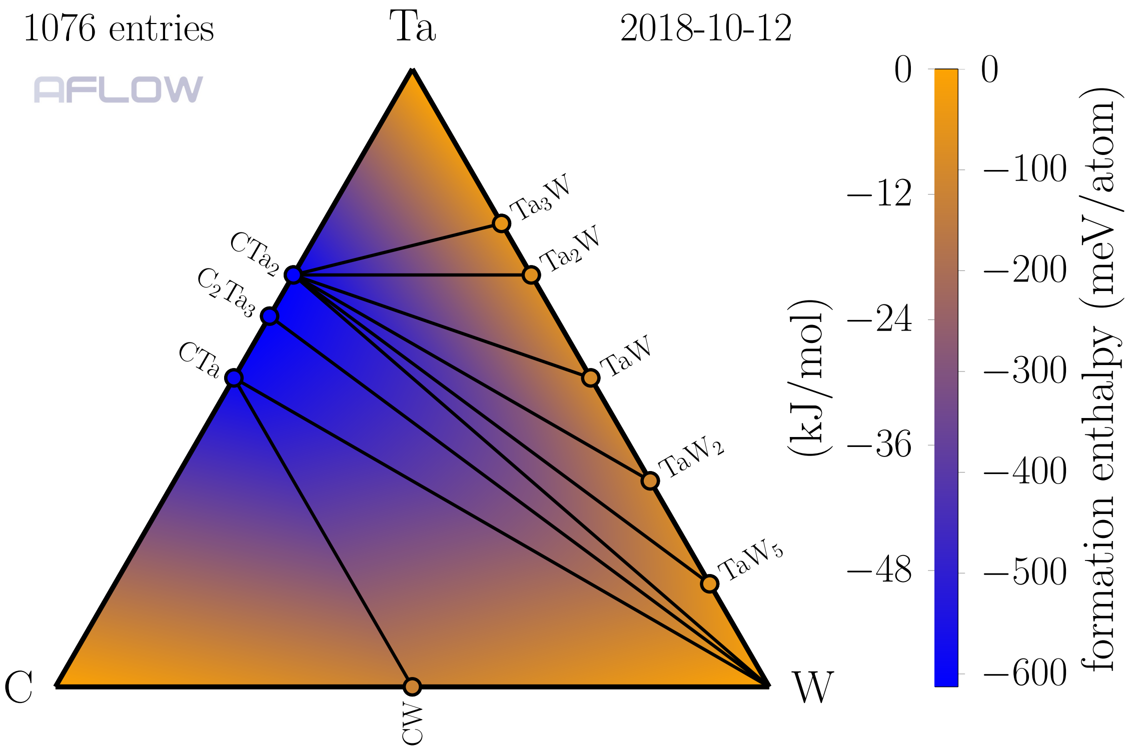

Supplementary Figure 29. CTaW ternary hull as calculated with AFLOW-CHULL.

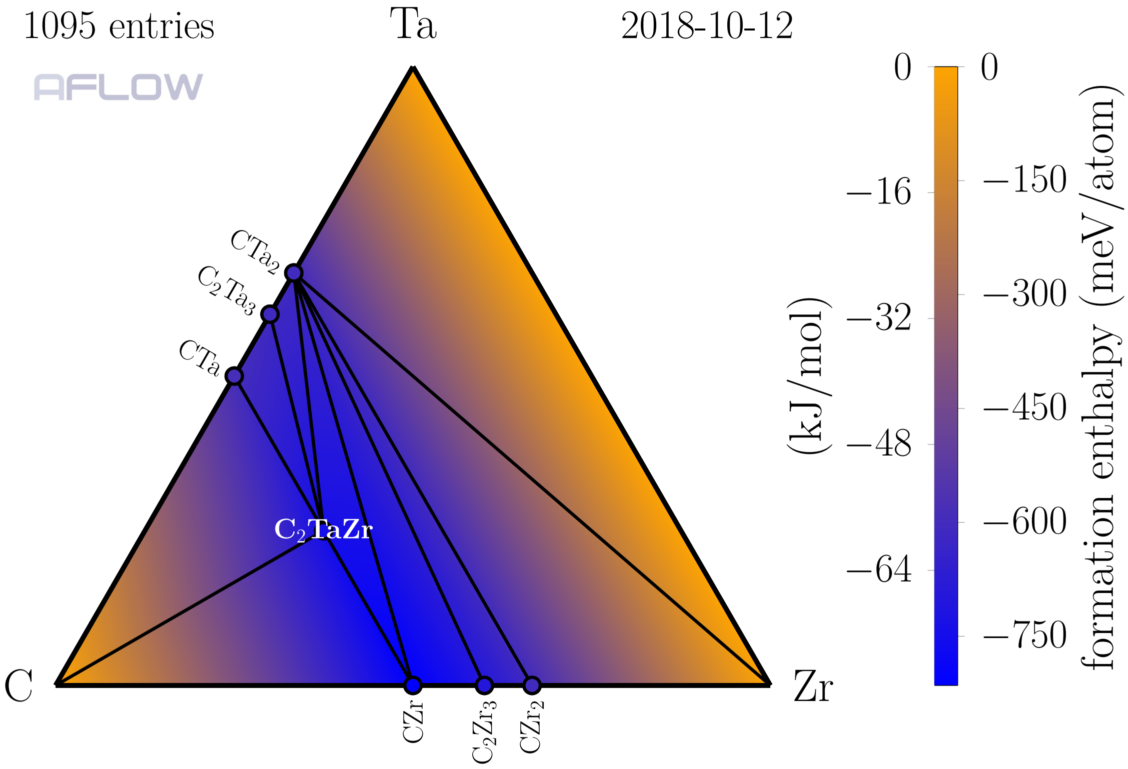

Supplementary Figure 30. CTaZr ternary hull as calculated with AFLOW-CHULL.

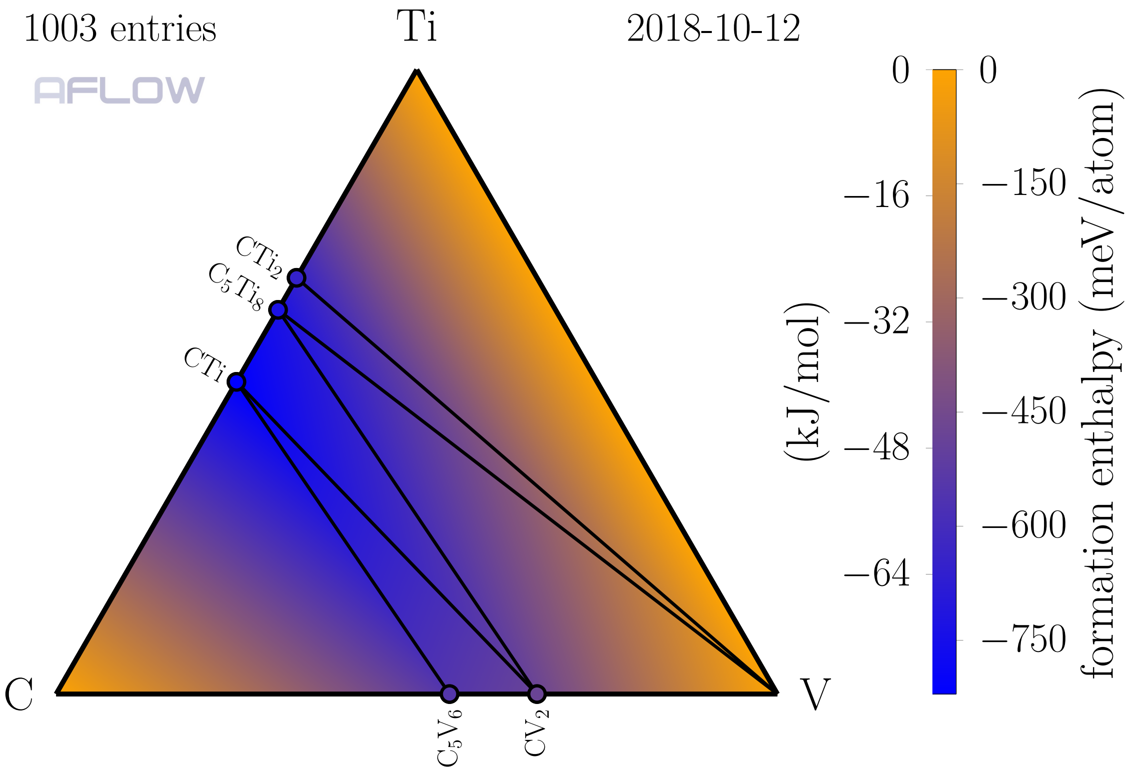

Supplementary Figure 31. CTiV ternary hull as calculated with AFLOW-CHULL.

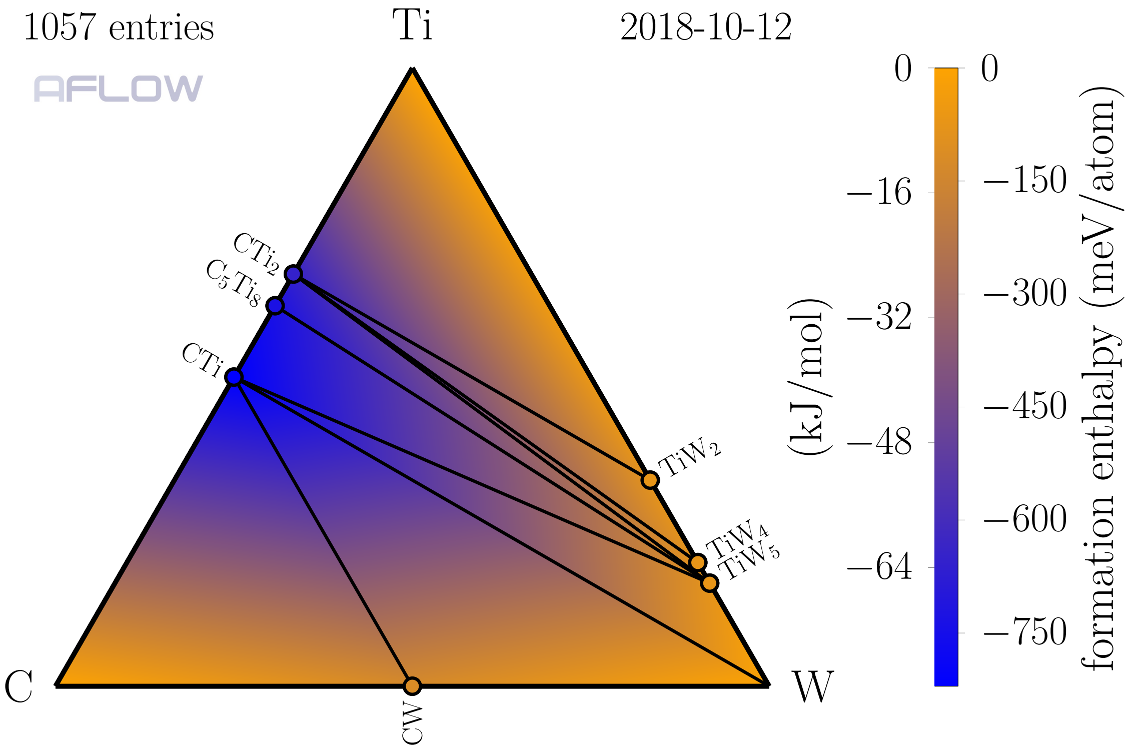

Supplementary Figure 32. CTiW ternary hull as calculated with AFLOW-CHULL.

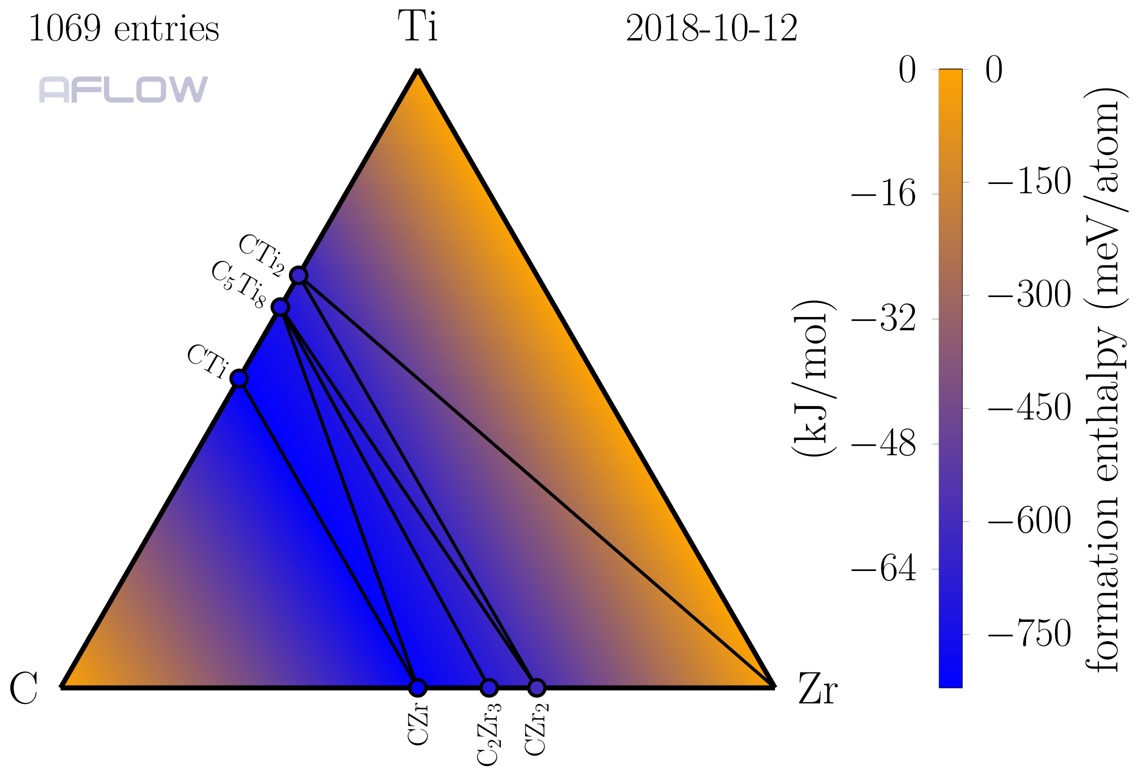

Supplementary Figure 33. CTiZr ternary hull as calculated with AFLOW-CHULL.

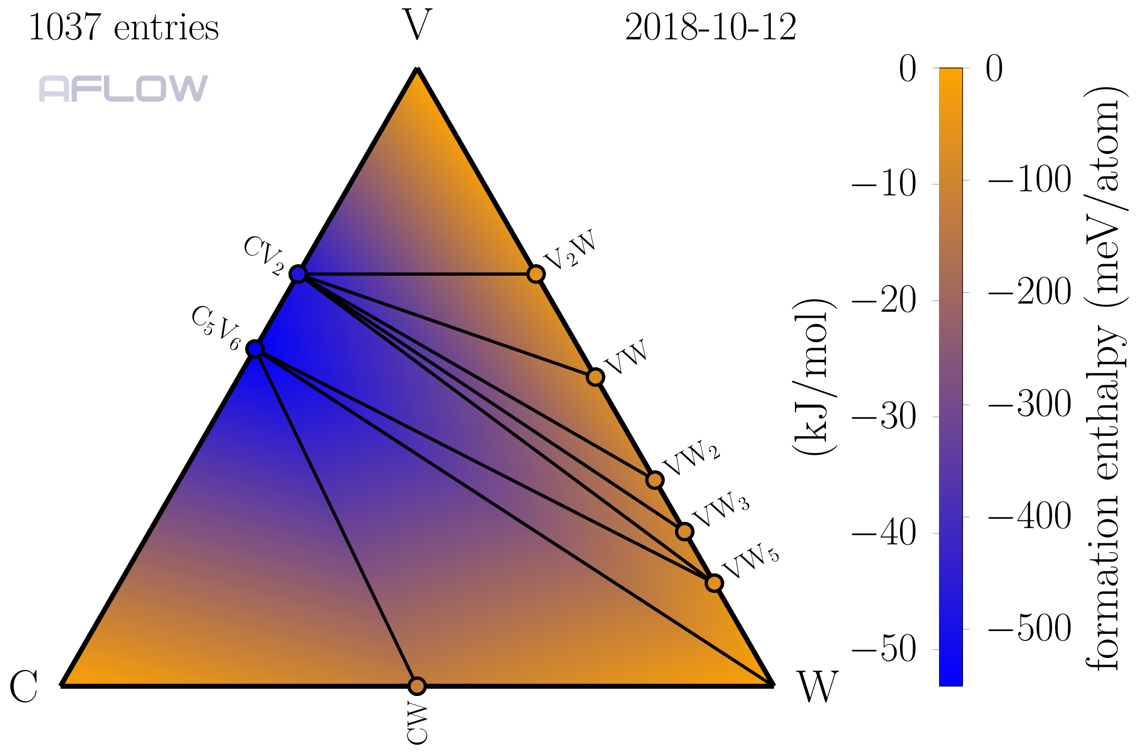

Supplementary Figure 34. CVW ternary hull as calculated with AFLOW-CHULL.

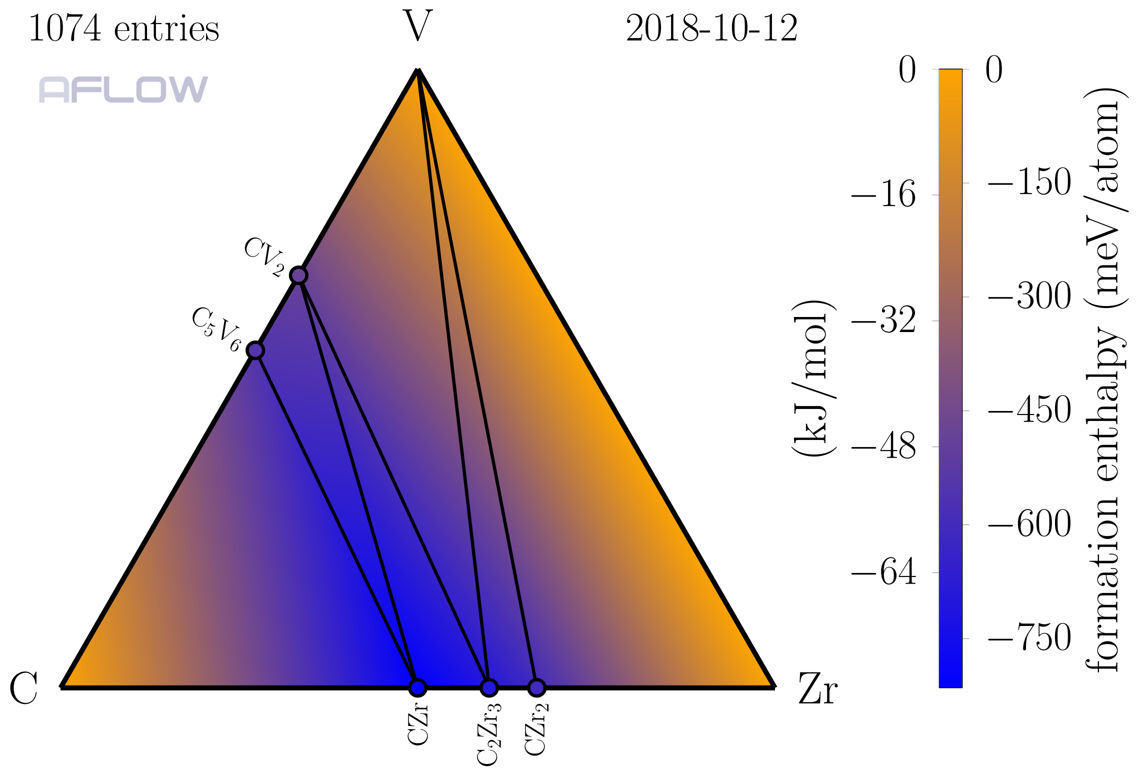

Supplementary Figure 35. CVZr ternary hull as calculated with AFLOW-CHULL.

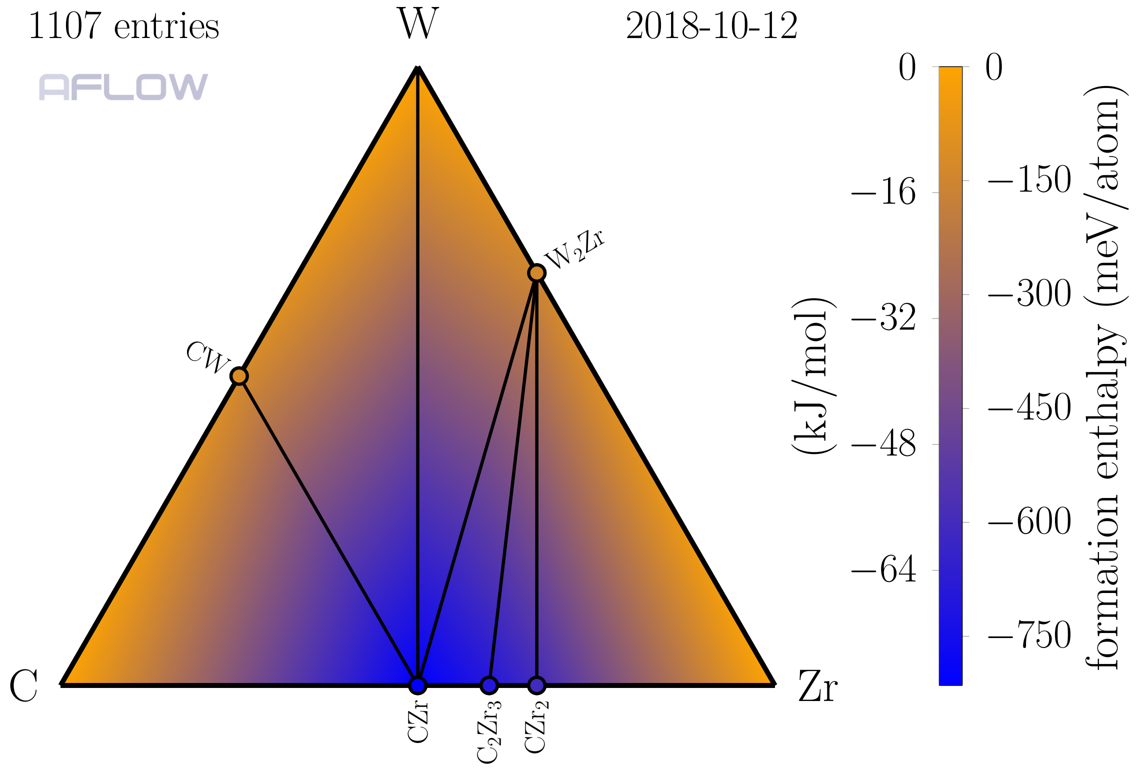

Supplementary Figure 36. CWZr ternary hull as calculated with AFLOW-CHULL.

Supplementary Table 1. Results for the calculated energetic distance from 6-dimensional convex hull, $\Delta H_{\rm f}$, and estimated synthesis temperature, $T_{\rm s}$, for the 5-metal carbide systems, arranged in descending order of EFA. Note that the compositions listed here are nominal, and the actual synthesized compositions can vary due to the presence of carbon vacancies in the anion sublattice. Units: $\Delta H_{\rm f}$ in (meV/atom) and $T_{\rm s}$ in K.

system	$\Delta H_{\rm f}$	$T_{\rm s}$	system	$\Delta H_{\rm f}$	$T_{\rm s}$	system	$\Delta H_{\rm f}$	$T_{\rm s}$	system	$\Delta H_{\rm f}$	$T_{\rm s}$
$MoNbTaVWC_5$	156	2254	$HfMoNbTaWC_5$	126	1811	$HfNbTiVWC_5$	81	1172	$HfMoNbVZrC_5$	89	1287
$HfNbTaTiZrC_5$	19	277	$HfMoNbTaVC_5$	99	1434	$HfNbTaWZrC_5$	53	764	$MoTaVWZrC_5$	148	2139
$HfNbTaTiVC_5$	56	807	$HfNbTaTiWC_5$	53	759	$NbTaVWZrC_5$	119	1718	$MoTaTiWZrC_5$	94	1359
$MoNbTaTiVC_5$	82	1185	$MoTaTiVWC_5$	128	1848	$HfTaTiVWC_5$	84	1210	$MoNbVWZrC_5$	146	2100
$NbTaTiVZrC_5$	64	922	$HfNbTaVZrC_5$	60	866	$HfMoTaVWC_5$	139	2010	$MoNbTiWZrC_5$	89	1282
$HfMoNbTaTiC_5$	48	689	$HfNbTaVWC_5$	110	1585	$HfMoNbVWC_5$	137	1977	$HfMoNbWZrC_5$	101	1452
$NbTaTiVWC_5$	92	1331	$HfMoTaTiVC_5$	82	1186	$HfNbTiWZrC_5$	56	811	$HfTiVWZrC_5$	99	1434
$MoNbTaTiWC_5$	111	1605	$HfMoNbTiZrC_5$	53	762	$HfMoTaTiWC_5$	84	1213	$HfNbVWZrC_5$	94	1359
$MoNbTiVWC_5$	122	1764	$MoNbTaWZrC_5$	133	1911	$HfMoNbTiWC_5$	81	1170	$HfMoTiVWC_5$	97	1394
$MoNbTaTiZrC_5$	57	815	$HfMoTaTiZrC_5$	55	799	$HfTaTiWZrC_5$	59	851	$HfMoTaWZrC_5$	105	1515
$HfTaTiVZrC_5$	73	1048	$NbTaTiWZrC_5$	61	879	$TaTiVWZrC_5$	96	1383	$HfTaVWZrC_5$	97	1395
$HfNbTiVZrC_5$	73	1058	$MoTaTiVZrC_5$	92	1330	$NbTiVWZrC_5$	93	1338	$MoTiVWZrC_5$	107	1541
$HfMoNbTiVC_5$	77	1112	$MoNbTiVZrC_5$	87	1259	$HfMoTiVZrC_5$	96	1380	$HfMoTiWZrC_5$	83	1192
$HfMoNbTaZrC_5$	48	690	$MoNbTaVZrC_5$	108	1562	$HfMoTaVZrC_5$	92	1326	$HfMoVWZrC_5$	141	2033

Supplementary Table 2. The predicted decomposition reaction products for the 56 5-metal carbides according to the AFLOW-CHULL module [1].

system	decomposition reaction products
$MoNbTaVWC_5$	0.0333333 C + 0.2 $C_{0.5}Mo_{0.5}$ + 0.2 $C_{0.5}Ta_{0.5}$ + 0.2 $C_{0.5}W_{0.5}$ + 0.183333 $C_{0.4545}Nb_{0.5455}$ + 0.183333 $C_{0.4545}V_{0.5455}$
$HfNbTaTiZrC_5$	0.4 $C_{0.5}Hf_{0.25}Nb_{0.25}$ + 0.4 $C_{0.5}Ta_{0.25}Ti_{0.25}$ + 0.2 $C_{0.5}Zr_{0.5}$
$HfNbTaTiVC_5$	0.0166667 C + 0.4 $C_{0.5}Hf_{0.25}Nb_{0.25}$ + 0.4 $C_{0.5}Ta_{0.25}Ti_{0.25}$ + 0.183333 $C_{0.4545}V_{0.5455}$
$MoNbTaTiVC_5$	0.0333333 C + 0.2 $C_{0.5}Mo_{0.5}$ + 0.4 $C_{0.5}Ta_{0.25}Ti_{0.25}$ + 0.183333 $C_{0.4545}Nb_{0.5455}$ + 0.183333 $C_{0.4545}V_{0.5455}$
$NbTaTiVZrC_5$	0.0166667 C + 0.4 $C_{0.5}Nb_{0.25}Zr_{0.25}$ + 0.4 $C_{0.5}Ta_{0.25}Ti_{0.25}$ + 0.183333 $C_{0.4545}V_{0.5455}$
$HfMoNbTaTiC_5$	0.4 $C_{0.5}Hf_{0.25}Nb_{0.25}$ + 0.2 $C_{0.5}Mo_{0.5}$ + 0.4 $C_{0.5}Ta_{0.25}Ti_{0.25}$
$NbTaTiVWC_5$	0.0333333 C + 0.4 $C_{0.5}Ta_{0.25}Ti_{0.25}$ + 0.2 $C_{0.5}W_{0.5}$ + 0.183333 $C_{0.4545}Nb_{0.5455}$ + 0.183333 $C_{0.4545}V_{0.5455}$
$MoNbTaTiWC_5$	0.0166667 C + 0.2 $C_{0.5}Mo_{0.5}$ + 0.4 $C_{0.5}Ta_{0.25}Ti_{0.25}$ + 0.2 $C_{0.5}W_{0.5}$ + 0.183333 $C_{0.4545}Nb_{0.5455}$
$MoNbTiVWC_5$	0.0166667 C + 0.2 $C_{0.5}Mo_{0.5}$ + 0.4 $C_{0.5}Nb_{0.25}Ti_{0.25}$ + 0.2 $C_{0.5}W_{0.5}$ + 0.183333 $C_{0.4545}V_{0.5455}$
$MoNbTaTiZrC_5$	0.2 $C_{0.5}Mo_{0.5}$ + 0.4 $C_{0.5}Nb_{0.25}Zr_{0.25}$ + 0.4 $C_{0.5}Ta_{0.25}Ti_{0.25}$
$HfTaTiVZrC_5$	0.0166667 C + 0.2 $C_{0.5}Hf_{0.5}$ + 0.4 $C_{0.5}Ta_{0.25}Ti_{0.25}$ + 0.2 $C_{0.5}Zr_{0.5}$ + 0.183333 $C_{0.4545}V_{0.5455}$
$HfNbTiVZrC_5$	0.0166667 C + 0.2 $C_{0.5}Hf_{0.5}$ + 0.4 $C_{0.5}Nb_{0.25}Ti_{0.25}$ + 0.2 $C_{0.5}Zr_{0.5}$ + 0.183333 $C_{0.4545}V_{0.5455}$
$HfMoNbTiVC_5$	0.0166667 C + 0.2 $C_{0.5}Hf_{0.5}$ + 0.2 $C_{0.5}Mo_{0.5}$ + 0.4 $C_{0.5}Nb_{0.25}Ti_{0.25}$ + 0.183333 $C_{0.4545}V_{0.5455}$
$HfMoNbTaZrC_5$	0.4 $C_{0.5}Hf_{0.25}Ta_{0.25}$ + 0.2 $C_{0.5}Mo_{0.5}$ + 0.4 $C_{0.5}Nb_{0.25}Zr_{0.25}$
$HfMoNbTaWC_5$	0.0166667 C + 0.4 $C_{0.5}Hf_{0.25}Ta_{0.25}$ + 0.2 $C_{0.5}Mo_{0.5}$ + 0.2 $C_{0.5}W_{0.5}$ + 0.183333 $C_{0.4545}Nb_{0.5455}$
$HfMoNbTaVC_5$	0.0333333 C + 0.4 $C_{0.5}Hf_{0.25}Ta_{0.25}$ + 0.2 $C_{0.5}Mo_{0.5}$ + 0.183333 $C_{0.4545}Nb_{0.5455}$ + 0.183333 $C_{0.4545}V_{0.5455}$
$HfNbTaTiWC_5$	0.4 $C_{0.5}Hf_{0.25}Nb_{0.25}$ + 0.4 $C_{0.5}Ta_{0.25}Ti_{0.25}$ + 0.2 $C_{0.5}W_{0.5}$
$MoTaTiVWC_5$	0.0166667 C + 0.2 $C_{0.5}Mo_{0.5}$ + 0.4 $C_{0.5}Ta_{0.25}Ti_{0.25}$ + 0.2 $C_{0.5}W_{0.5}$ + 0.183333 $C_{0.4545}V_{0.5455}$
$HfNbTaVZrC_5$	0.0166667 C + 0.4 $C_{0.5}Hf_{0.25}Ta_{0.25}$ + 0.4 $C_{0.5}Nb_{0.25}Zr_{0.25}$ + 0.183333 $C_{0.4545}V_{0.5455}$
$HfNbTaVWC_5$	0.0333333 C + 0.4 $C_{0.5}Hf_{0.25}Ta_{0.25}$ + 0.2 $C_{0.5}W_{0.5}$ + 0.183333 $C_{0.4545}Nb_{0.5455}$ + 0.183333 $C_{0.4545}V_{0.5455}$
$HfMoTaTiVC_5$	0.0166667 C + 0.2 $C_{0.5}Hf_{0.5}$ + 0.2 $C_{0.5}Mo_{0.5}$ + 0.4 $C_{0.5}Ta_{0.25}Ti_{0.25}$ + 0.183333 $C_{0.4545}V_{0.5455}$
$HfMoNbTiZrC_5$	0.2 $C_{0.5}Hf_{0.5}$ + 0.2 $C_{0.5}Mo_{0.5}$ + 0.4 $C_{0.5}Nb_{0.25}Ti_{0.25}$ + 0.2 $C_{0.5}Zr_{0.5}$
$MoNbTaWZrC_5$	0.0166667 C + 0.2 $C_{0.5}Mo_{0.5}$ + 0.4 $C_{0.5}Ta_{0.25}Zr_{0.25}$ + 0.2 $C_{0.5}W_{0.5}$ + 0.183333 $C_{0.4545}Nb_{0.5455}$
$HfMoTaTiZrC_5$	0.2 $C_{0.5}Hf_{0.5}$ + 0.2 $C_{0.5}Mo_{0.5}$ + 0.4 $C_{0.5}Ta_{0.25}Ti_{0.25}$ + 0.2 $C_{0.5}Zr_{0.5}$
$NbTaTiWZrC_5$	0.4 $C_{0.5}Nb_{0.25}Zr_{0.25}$ + 0.4 $C_{0.5}Ta_{0.25}Ti_{0.25}$ + 0.2 $C_{0.5}W_{0.5}$
$MoTaTiVZrC_5$	0.0166667 C + 0.2 $C_{0.5}Mo_{0.5}$ + 0.4 $C_{0.5}Ta_{0.25}Ti_{0.25}$ + 0.2 $C_{0.5}Zr_{0.5}$ + 0.183333 $C_{0.4545}V_{0.5455}$
$MoNbTiVZrC_5$	0.0166667 C + 0.2 $C_{0.5}Mo_{0.5}$ + 0.4 $C_{0.5}Nb_{0.25}Ti_{0.25}$ + 0.2 $C_{0.5}Zr_{0.5}$ + 0.183333 $C_{0.4545}V_{0.5455}$
$MoNbTaVZrC_5$	0.0333333 C + 0.2 $C_{0.5}Mo_{0.5}$ + 0.4 $C_{0.5}Ta_{0.25}Zr_{0.25}$ + 0.183333 $C_{0.4545}Nb_{0.5455}$ + 0.183333 $C_{0.4545}V_{0.5455}$
$HfNbTiVWC_5$	0.0166667 C + 0.2 $C_{0.5}Hf_{0.5}$ + 0.4 $C_{0.5}Nb_{0.25}Ti_{0.25}$ + 0.2 $C_{0.5}W_{0.5}$ + 0.183333 $C_{0.4545}V_{0.5455}$
$HfNbTaWZrC_5$	0.4 $C_{0.5}Hf_{0.25}Ta_{0.25}$ + 0.4 $C_{0.5}Nb_{0.25}Zr_{0.25}$ + 0.2 $C_{0.5}W_{0.5}$
$NbTaVWZrC_5$	0.0333333 C + 0.4 $C_{0.5}Ta_{0.25}Zr_{0.25}$ + 0.2 $C_{0.5}W_{0.5}$ + 0.183333 $C_{0.4545}Nb_{0.5455}$ + 0.183333 $C_{0.4545}V_{0.5455}$
$HfTaTiVWC_5$	0.0166667 C + 0.2 $C_{0.5}Hf_{0.5}$ + 0.4 $C_{0.5}Ta_{0.25}Ti_{0.25}$ + 0.2 $C_{0.5}W_{0.5}$ + 0.183333 $C_{0.4545}V_{0.5455}$
$HfMoTaVWC_5$	0.0166667 C + 0.4 $C_{0.5}Hf_{0.25}Ta_{0.25}$ + 0.2 $C_{0.5}Mo_{0.5}$ + 0.2 $C_{0.5}W_{0.5}$ + 0.183333 $C_{0.4545}V_{0.5455}$
$HfMoNbVWC_5$	0.0166667 C + 0.4 $C_{0.5}Hf_{0.25}Nb_{0.25}$ + 0.2 $C_{0.5}Mo_{0.5}$ + 0.2 $C_{0.5}W_{0.5}$ + 0.183333 $C_{0.4545}V_{0.5455}$
$HfNbTiWZrC_5$	0.2 $C_{0.5}Hf_{0.5}$ + 0.4 $C_{0.5}Nb_{0.25}Ti_{0.25}$ + 0.2 $C_{0.5}W_{0.5}$ + 0.2 $C_{0.5}Zr_{0.5}$
$HfMoTaTiWC_5$	0.2 $C_{0.5}Hf_{0.5}$ + 0.2 $C_{0.5}Mo_{0.5}$ + 0.4 $C_{0.5}Ta_{0.25}Ti_{0.25}$ + 0.2 $C_{0.5}W_{0.5}$
$HfMoNbTiWC_5$	0.2 $C_{0.5}Hf_{0.5}$ + 0.2 $C_{0.5}Mo_{0.5}$ + 0.4 $C_{0.5}Nb_{0.25}Ti_{0.25}$ + 0.2 $C_{0.5}W_{0.5}$
$HfTaTiWZrC_5$	0.2 $C_{0.5}Hf_{0.5}$ + 0.4 $C_{0.5}Ta_{0.25}Ti_{0.25}$ + 0.2 $C_{0.5}W_{0.5}$ + 0.2 $C_{0.5}Zr_{0.5}$
$TaTiVWZrC_5$	0.0166667 C + 0.4 $C_{0.5}Ta_{0.25}Ti_{0.25}$ + 0.2 $C_{0.5}W_{0.5}$ + 0.2 $C_{0.5}Zr_{0.5}$ + 0.183333 $C_{0.4545}V_{0.5455}$
$NbTiVWZrC_5$	0.0166667 C + 0.4 $C_{0.5}Nb_{0.25}Ti_{0.25}$ + 0.2 $C_{0.5}W_{0.5}$ + 0.2 $C_{0.5}Zr_{0.5}$ + 0.183333 $C_{0.4545}V_{0.5455}$
$HfMoTiVZrC_5$	0.0166667 C + 0.2 $C_{0.5}Hf_{0.5}$ + 0.2 $C_{0.5}Mo_{0.5}$ + 0.2 $C_{0.5}Ti_{0.5}$ + 0.2 $C_{0.5}Zr_{0.5}$ + 0.183333 $C_{0.4545}V_{0.5455}$
$HfMoTaVZrC_5$	0.0166667 C + 0.4 $C_{0.5}Hf_{0.25}Ta_{0.25}$ + 0.2 $C_{0.5}Mo_{0.5}$ + 0.2 $C_{0.5}Zr_{0.5}$ + 0.183333 $C_{0.4545}V_{0.5455}$
$HfMoNbVZrC_5$	0.0166667 C + 0.4 $C_{0.5}Hf_{0.25}Nb_{0.25}$ + 0.2 $C_{0.5}Mo_{0.5}$ + 0.2 $C_{0.5}Zr_{0.5}$ + 0.183333 $C_{0.4545}V_{0.5455}$
$MoTaVWZrC_5$	0.0166667 C + 0.2 $C_{0.5}Mo_{0.5}$ + 0.4 $C_{0.5}Ta_{0.25}Zr_{0.25}$ + 0.2 $C_{0.5}W_{0.5}$ + 0.183333 $C_{0.4545}V_{0.5455}$
$MoTaTiWZrC_5$	0.2 $C_{0.5}Mo_{0.5}$ + 0.4 $C_{0.5}Ta_{0.25}Ti_{0.25}$ + 0.2 $C_{0.5}W_{0.5}$ + 0.2 $C_{0.5}Zr_{0.5}$
$MoNbVWZrC_5$	0.0166667 C + 0.2 $C_{0.5}Mo_{0.5}$ + 0.4 $C_{0.5}Nb_{0.25}Zr_{0.25}$ + 0.2 $C_{0.5}W_{0.5}$ + 0.183333 $C_{0.4545}V_{0.5455}$
$MoNbTiWZrC_5$	0.2 $C_{0.5}Mo_{0.5}$ + 0.4 $C_{0.5}Nb_{0.25}Ti_{0.25}$ + 0.2 $C_{0.5}W_{0.5}$ + 0.2 $C_{0.5}Zr_{0.5}$
$HfMoNbWZrC_5$	0.4 $C_{0.5}Hf_{0.25}Nb_{0.25}$ + 0.2 $C_{0.5}Mo_{0.5}$ + 0.2 $C_{0.5}W_{0.5}$ + 0.2 $C_{0.5}Zr_{0.5}$
$HfTiVWZrC_5$	0.0166667 C + 0.2 $C_{0.5}Hf_{0.5}$ + 0.2 $C_{0.5}Ti_{0.5}$ + 0.2 $C_{0.5}W_{0.5}$ + 0.2 $C_{0.5}Zr_{0.5}$ + 0.183333 $C_{0.4545}V_{0.5455}$
$HfNbVWZrC_5$	0.0166667 C + 0.4 $C_{0.5}Hf_{0.25}Nb_{0.25}$ + 0.2 $C_{0.5}W_{0.5}$ + 0.2 $C_{0.5}Zr_{0.5}$ + 0.183333 $C_{0.4545}V_{0.5455}$
$HfMoTiVWC_5$	0.0166667 C + 0.2 $C_{0.5}Hf_{0.5}$ + 0.2 $C_{0.5}Mo_{0.5}$ + 0.2 $C_{0.5}Ti_{0.5}$ + 0.2 $C_{0.5}W_{0.5}$ + 0.183333 $C_{0.4545}V_{0.5455}$
$HfMoTaWZrC_5$	0.4 $C_{0.5}Hf_{0.25}Ta_{0.25}$ + 0.2 $C_{0.5}Mo_{0.5}$ + 0.2 $C_{0.5}W_{0.5}$ + 0.2 $C_{0.5}Zr_{0.5}$
$HfTaVWZrC_5$	0.0166667 C + 0.4 $C_{0.5}Hf_{0.25}Ta_{0.25}$ + 0.2 $C_{0.5}W_{0.5}$ + 0.2 $C_{0.5}Zr_{0.5}$ + 0.183333 $C_{0.4545}V_{0.5455}$
$MoTiVWZrC_5$	0.0166667 C + 0.2 $C_{0.5}Mo_{0.5}$ + 0.2 $C_{0.5}Ti_{0.5}$ + 0.2 $C_{0.5}W_{0.5}$ + 0.2 $C_{0.5}Zr_{0.5}$ + 0.183333 $C_{0.4545}V_{0.5455}$
$HfMoTiWZrC_5$	0.2 $C_{0.5}Hf_{0.5}$ + 0.2 $C_{0.5}Mo_{0.5}$ + 0.2 $C_{0.5}Ti_{0.5}$ + 0.2 $C_{0.5}W_{0.5}$ + 0.2 $C_{0.5}Zr_{0.5}$
$HfMoVWZrC_5$	0.0166667 C + 0.2 $C_{0.5}Hf_{0.5}$ + 0.2 $C_{0.5}Mo_{0.5}$ + 0.2 $C_{0.5}W_{0.5}$ + 0.2 $C_{0.5}Zr_{0.5}$ + 0.183333 $C_{0.4545}V_{0.5455}$

SUPPLEMENTARY NOTE 3: EFFECT OF PRECURSOR CHOICE ON SYNTHESIZABILITY

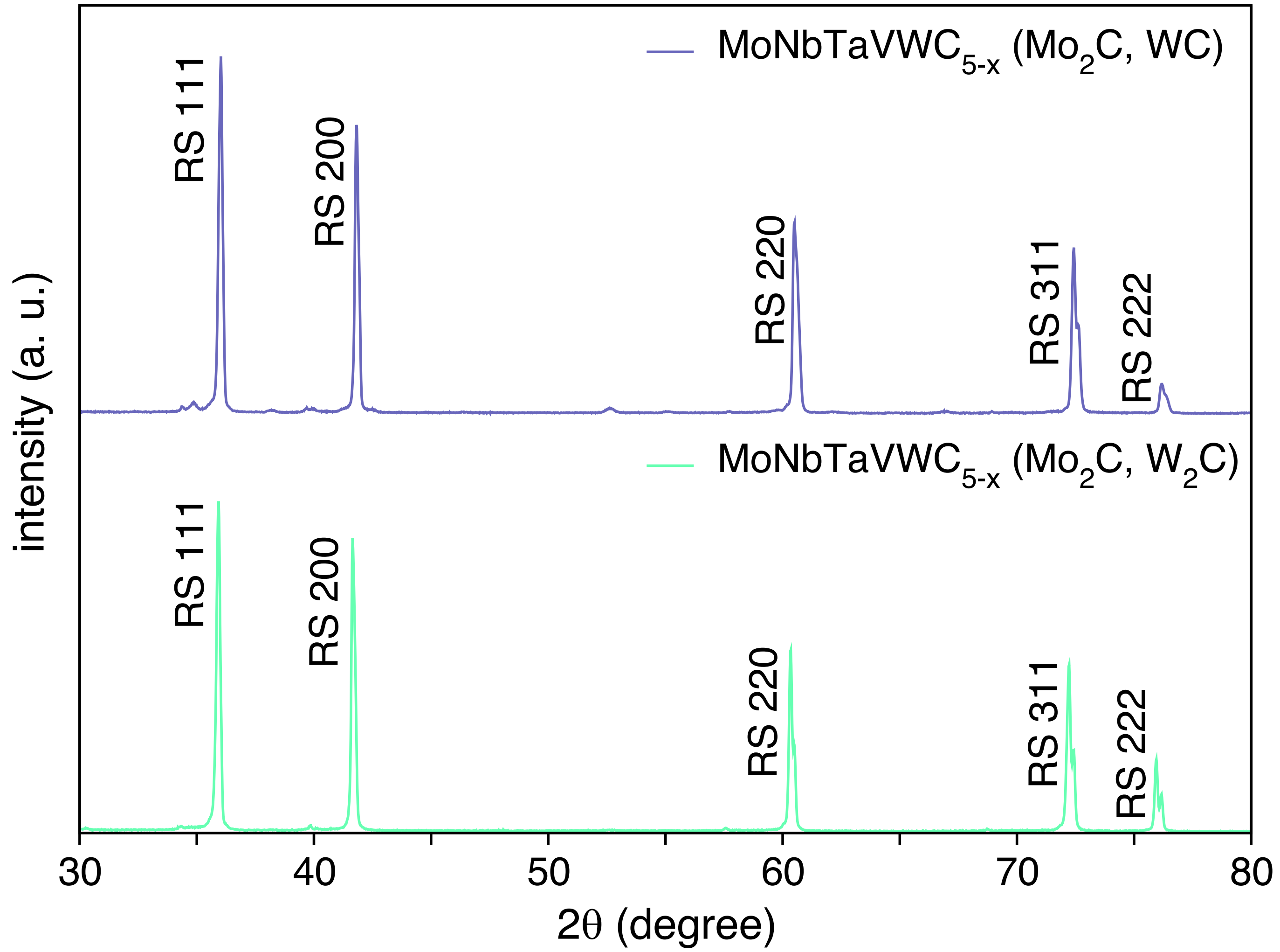

Supplementary Figure 37. **X-ray diffraction spectra for MoNbTaVWC$_{5-x}$. (Top)** Sample synthesized using Mo_2C and WC. **(Bottom)** Sample synthesized using Mo_2C and W_2C.

MoNbTaVWC$_{5-x}$ was synthesized using both hexagonal WC and W_2C precursors, to determine their impact on the homogeneity of the final sample. The x-ray diffraction spectrum for the sample synthesized using WC is displayed in the top panel of Figure 37, while the spectrum for the sample prepared with W_2C is shown in the bottom panel. Both spectra feature sharp peaks at similar values of 2θ, indicating that the choice of precursor has little effect on the structure of the high-entropy material.

SUPPLEMENTARY NOTE 4: MECHANICAL PROPERTIES

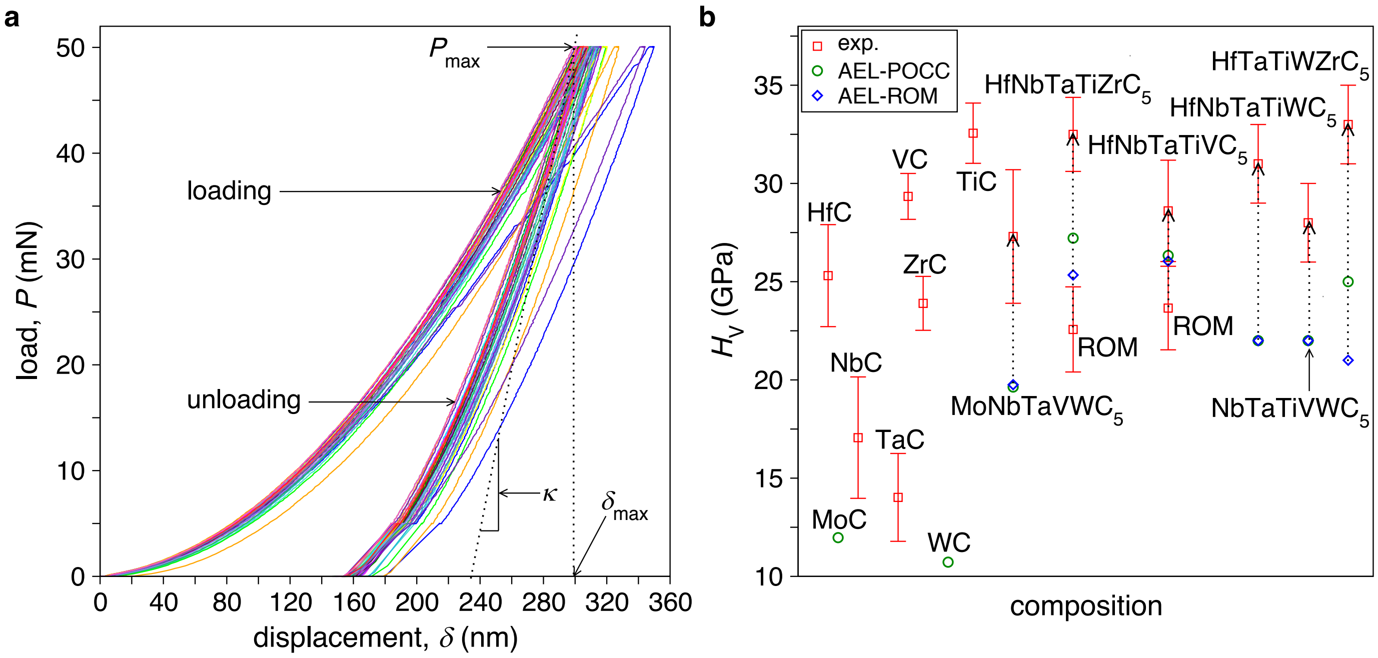

Supplementary Figure 38. **Mechanical properties.** **(a)** Load-displacement curves for 40 indents for $HfNbTaTiZrC_5$ at a maximum load of 50mN. This curve provides the three necessary parameters — the peak load: P_{max}, the depth at peak load: δ_{max}, and the initial unloading contact stiffness: κ, (indicated by arrows for an indent) — for obtaining Vickers hardness: H_V, and elastic modulus: E [2]. **(b)** Comparison of calculated and measured H_V for 6 single-phase 5-metal carbides. All 5-metal carbides have a higher hardness than expected from their respective ROM predictions (indicated by the dotted upward arrows), whereas the calculated H_V (green circles) are consistent with the ROM for the AFLOW-AEL results (blue diamonds). The measured H_V for the 6 rock-salt structure binary carbide samples along with the calculated H_V for rock-salt MoC and WC are also plotted. Error bars represent the standard deviations for a series of 40 indents.

The experimentally measured load-displacement indentation curves for the hardness measurements for the $HfNbTaTiZrC_5$ sample are shown in Figure 38(a). Each curve provides the three parameters necessary to obtain the Vickers hardness H_V and elastic modulus E [2, 3]: the peak load: P_{max}, the depth at peak load: δ_{max}, and the initial unloading contact stiffness: κ.

The measured and calculated H_V for 6 5-metal carbides along with 8 rock-salt binary carbides are plotted in Figure 38(b). The calculated H_V are obtained from the thermally averaged bulk (B) and shear (G) moduli, weighted according to the Boltzmann distribution at a temperature of 2200°C (the experimental sintering temperature), using the model of Chen *et al.* ($H_V = 2(k^2G)^{0.585} - 3$; $k = G/B$) [4]. The experimentally measured hardness for all 5-metal carbides exceeds the rule of mixture (ROM) predictions, whereas the calculated values are consistent with the ROM for the AFLOW-AEL (Automatic Elasticity Library) results. The atomic disorder is not accounted for in the AFLOW-AEL calculations of the AFLOW-POCC ordered-configurations, suggesting that the measured enhancement in H_V is disorder-driven. For $HfNbTaTiZrC_5$, H_V is about 50% higher than the ROM estimate. Note that the ROM predictions for Mo and/or W containing 5-metal carbides are obtained from the AFLOW-AEL calculations, since MoC and WC do not stabilize in a rock-salt phase at ambient temperature.

The values of B and G for each of the 49 configurations of 6 5-metal carbides are listed in Supplementary Table 3. These elastic moduli are calculated using the Voigt-Reuss-Hill (VRH) average within the AEL module [5] of the AFLOW framework.

Supplementary Table 3. B and G for each configuration of 6 5-metal carbides, as calculated using AFLOW-AEL. Units: B, G in GPa.

conf.	$MoNbTaVWC_5$		$HfNbTaTiZrC_5$		$HfNbTaTiVC_5$		$HfNbTaTiWC_5$		$NbTaTiVWC_5$		$HfTaTiWZrC_5$	
	B	G	B	G	B	G	B	G	B	G	B	G
1	322	166	262	189	284	193	280	188	305	189	274	189
2	317	198	259	186	270	194	290	201	340	168	269	183
3	312	191	260	190	278	191	282	201	303	189	275	193
4	308	195	262	187	281	194	283	205	307	187	270	186
5	315	199	260	192	274	193	283	197	304	200	272	187
6	313	175	260	190	271	192	286	203	314	183	275	182
7	319	189	262	192	273	199	290	201	304	201	277	190
8	310	184	261	190	278	197	293	199	330	189	276	191
9	322	194	261	194	273	196	283	196	309	201	270	187
10	313	191	261	189	273	195	287	198	307	201	271	178
11	318	192	260	195	275	194	283	200	303	202	272	189
12	307	177	262	191	276	193	291	199	304	200	280	197
13	316	188	258	189	280	200	290	203	306	200	269	181
14	305	188	260	190	277	197	291	198	303	199	273	185
15	318	186	258	192	278	197	293	203	305	170	276	196
16	316	187	260	189	274	192	290	198	304	198	273	183
17	305	188	263	195	279	198	285	200	309	189	273	183
18	316	190	262	193	280	198	283	195	346	172	272	183
19	314	196	264	194	276	194	295	204	299	201	273	189
20	318	187	261	192	277	198	296	201	303	199	272	187
21	325	188	262	195	275	193	291	203	304	200	274	189
22	321	187	262	190	279	196	294	201	305	201	272	181
23	315	171	262	194	276	195	292	205	304	200	277	191
24	321	193	262	192	283	202	296	206	312	181	273	183
25	319	189	262	191	276	197	292	199	297	201	274	190
26	314	173	262	191	274	194	292	204	293	195	276	194
27	317	186	264	193	273	194	292	201	305	205	276	193
28	316	192	264	192	274	192	292	201	304	203	275	195
29	322	188	266	196	272	191	294	205	295	199	273	191
30	314	181	262	191	273	192	291	201	299	197	273	190
31	265	169	264	194	276	196	288	203	300	200	275	194
32	277	181	265	195	276	197	293	202	296	198	274	189
33	318	190	262	190	277	194	294	204	308	206	273	192
34	277	183	263	192	276	195	291	201	305	204	274	189
35	324	189	261	189	274	194	290	202	294	199	279	196
36	318	182	262	194	276	191	294	203	301	204	279	195
37	316	182	263	192	277	196	294	203	302	180	273	187
38	315	182	262	192	276	195	292	203	298	199	272	189
39	313	188	262	192	276	196	295	208	298	199	274	194
40	270	127	262	192	282	198	291	202	306	203	272	194
41	315	174	264	191	277	197	287	201	306	203	275	193
42	319	184	263	193	276	197	291	204	304	202	275	193
43	313	171	264	195	274	192	292	204	297	198	273	192
44	312	181	263	192	275	197	295	206	307	205	274	191
45	314	180	263	194	276	194	292	196	307	204	273	191
46	317	183	263	191	277	193	294	200	305	204	274	191
47	325	190	265	194	277	198	292	203	304	204	275	193
48	320	192	266	193	279	197	292	201	304	205	278	195
49	311	177	264	193	275	196	295	204	306	203	275	195

SUPPLEMENTARY NOTE 5: ELECTRONIC DENSITY OF STATES

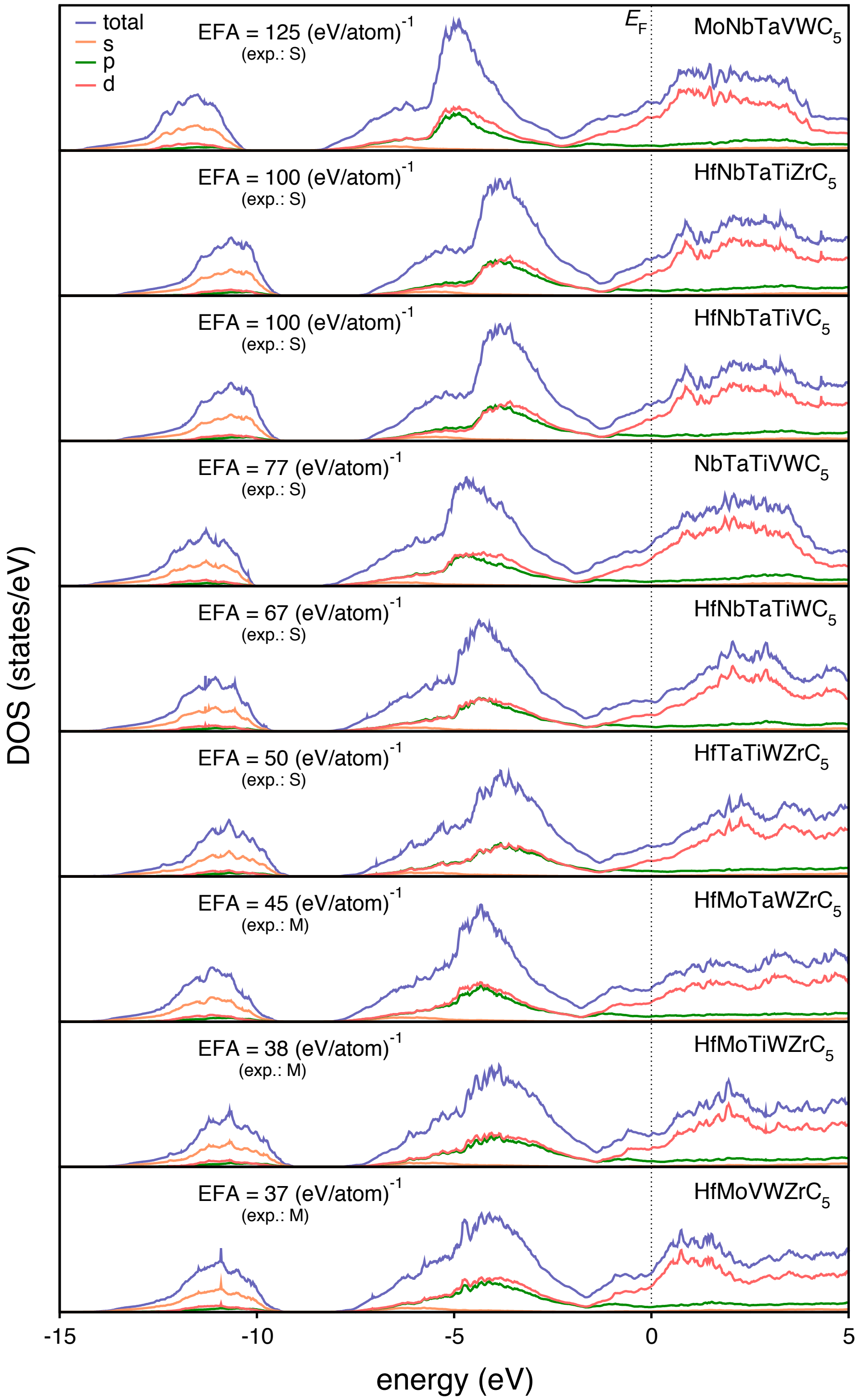

Supplementary Figure 39. **Electronic density of states (DOS).** The AFLOW-POCC ensemble averaged DOS [6] for the 9 synthesized 5-metal carbides, calculated at the experimental sintering temperature, 2200°C. "E_F": Fermi energy."S": single-phase formed; "M": multi-phase formed in experiment.

SUPPLEMENTARY NOTE 6: FORMATION ENTHALPIES

The formation enthalpies (H_{f}) for each of the 49 configurations of all 56 5-metal carbide compositions are listed in Supplementary Tables 4-10. Carbides are arranged from left to right in each table in descending order of EFA. Each enthalpy value also acts as a link to the AFLOW.org entry page for the corresponding composition and configuration, which contains crystallographic, thermodynamic, and electronic structure data for the system.

H_{f} are calculated using

$$H_{\mathrm{f}} = H_{\mathrm{POCC},i} - \sum_{\alpha}^{N} x_{\alpha} H_{\alpha} \tag{1}$$

where $H_{\mathrm{POCC},i}$ is the enthalpy per atom for each POCC configuration of the N-metal carbide, H_{α} are the enthalpies per atom for the corresponding constituent species α in their standard elemental phases, and x_{α} is the molar concentration of species α.

Supplementary Table 4. Formation enthalpies (H_f) with links to AFLOW.org entry pages for each calculated configuration. Units: H_f in eV/atom.

conf.	$MoNbTaVWC_5$	$HfNbTaTiZrC_5$	$HfNbTaTiVC_5$	$MoNbTaTiVC_5$	$HfMoNbTaTiC_5$	$NbTaTiVZrC_5$	$NbTaTiVWC_5$	$MoNbTaTiWC_5$
	H_f	H_f	H_f	H_f	H_f	H_f	H_f	H_f
1	-0.192	-0.754	-0.639	-0.42	-0.577	-0.601	-0.425	-0.307
2	-0.203	-0.734	-0.647	-0.43	-0.562	-0.612	-0.411	-0.329
3	-0.198	-0.757	-0.655	-0.427	-0.557	-0.62	-0.416	-0.311
4	-0.206	-0.735	-0.652	-0.432	-0.555	-0.617	-0.413	-0.331
5	-0.2	-0.736	-0.622	-0.423	-0.552	-0.578	-0.416	-0.316
6	-0.185	-0.756	-0.623	-0.423	-0.552	-0.578	-0.42	-0.31
7	-0.19	-0.736	-0.648	-0.428	-0.567	-0.613	-0.415	-0.312
8	-0.197	-0.755	-0.635	-0.424	-0.544	-0.597	-0.43	-0.326
9	-0.199	-0.734	-0.638	-0.429	-0.578	-0.595	-0.408	-0.315
10	-0.2	-0.726	-0.636	-0.428	-0.54	-0.594	-0.406	-0.317
11	-0.203	-0.737	-0.622	-0.423	-0.552	-0.577	-0.423	-0.328
12	-0.198	-0.727	-0.62	-0.422	-0.572	-0.576	-0.423	-0.315
13	-0.2	-0.753	-0.658	-0.442	-0.534	-0.623	-0.431	-0.322
14	-0.197	-0.733	-0.648	-0.438	-0.557	-0.611	-0.435	-0.306
15	-0.199	-0.756	-0.64	-0.432	-0.535	-0.6	-0.422	-0.323
16	-0.201	-0.734	-0.635	-0.437	-0.568	-0.593	-0.434	-0.321
17	-0.199	-0.746	-0.65	-0.432	-0.555	-0.602	-0.44	-0.319
18	-0.197	-0.716	-0.649	-0.44	-0.531	-0.611	-0.437	-0.308
19	-0.205	-0.744	-0.639	-0.441	-0.556	-0.609	-0.426	-0.324
20	-0.198	-0.714	-0.636	-0.436	-0.553	-0.598	-0.425	-0.319
21	-0.199	-0.735	-0.636	-0.433	-0.542	-0.594	-0.44	-0.328
22	-0.198	-0.726	-0.639	-0.432	-0.565	-0.599	-0.436	-0.326
23	-0.187	-0.737	-0.647	-0.437	-0.565	-0.61	-0.442	-0.335
24	-0.209	-0.744	-0.655	-0.436	-0.549	-0.62	-0.428	-0.31
25	-0.21	-0.743	-0.628	-0.457	-0.56	-0.582	-0.454	-0.336
26	-0.19	-0.729	-0.627	-0.449	-0.554	-0.604	-0.448	-0.334
27	-0.207	-0.746	-0.643	-0.435	-0.583	-0.611	-0.426	-0.312
28	-0.209	-0.743	-0.649	-0.447	-0.577	-0.581	-0.424	-0.331
29	-0.205	-0.757	-0.65	-0.449	-0.556	-0.612	-0.444	-0.332
30	-0.213	-0.755	-0.65	-0.457	-0.569	-0.612	-0.444	-0.341
31	-0.214	-0.749	-0.648	-0.433	-0.569	-0.609	-0.448	-0.345
32	-0.219	-0.755	-0.643	-0.449	-0.538	-0.604	-0.455	-0.352
33	-0.206	-0.733	-0.615	-0.445	-0.537	-0.567	-0.441	-0.333
34	-0.22	-0.756	-0.612	-0.43	-0.569	-0.564	-0.439	-0.352
35	-0.203	-0.732	-0.65	-0.456	-0.547	-0.612	-0.45	-0.332
36	-0.216	-0.749	-0.644	-0.455	-0.565	-0.605	-0.45	-0.344
37	-0.209	-0.754	-0.629	-0.455	-0.576	-0.607	-0.452	-0.342
38	-0.213	-0.751	-0.645	-0.451	-0.564	-0.607	-0.451	-0.342
39	-0.201	-0.754	-0.645	-0.445	-0.564	-0.584	-0.451	-0.331
40	-0.214	-0.751	-0.627	-0.444	-0.578	-0.583	-0.432	-0.343
41	-0.213	-0.755	-0.639	-0.453	-0.57	-0.596	-0.44	-0.342
42	-0.197	-0.746	-0.642	-0.454	-0.578	-0.601	-0.436	-0.328
43	-0.214	-0.749	-0.643	-0.435	-0.573	-0.604	-0.449	-0.343
44	-0.217	-0.755	-0.648	-0.437	-0.561	-0.609	-0.431	-0.345
45	-0.209	-0.749	-0.63	-0.442	-0.57	-0.586	-0.45	-0.342
46	-0.198	-0.745	-0.628	-0.437	-0.562	-0.585	-0.449	-0.33
47	-0.21	-0.747	-0.642	-0.447	-0.557	-0.597	-0.448	-0.339
48	-0.209	-0.746	-0.643	-0.442	-0.553	-0.602	-0.442	-0.338
49	-0.214	-0.748	-0.639	-0.446	-0.567	-0.597	-0.445	-0.347

Supplementary Table 5. Formation enthalpies (H_f) with links to AFLOW.org entry pages for each calculated configuration. Units: H_f in eV/atom.

conf.	$MoNbTaTiZrC_5$	$HfMoNbTaZrC_5$	$HfTaTiVZrC_5$	$HfNbTiVZrC_5$	$HfMoNbTiVC_5$	$HfMoNbTaVC_5$	$MoNbTiVWC_5$	$HfMoNbTaWC_5$
	H_f	H_f	H_f	H_f	H_f	H_f	H_f	H_f
1	-0.544	-0.563	-0.688	-0.654	-0.5	-0.448	-0.256	-0.323
2	-0.53	-0.564	-0.684	-0.666	-0.516	-0.44	-0.276	-0.33
3	-0.52	-0.527	-0.685	-0.655	-0.478	-0.439	-0.264	-0.309
4	-0.514	-0.55	-0.67	-0.665	-0.496	-0.424	-0.273	-0.335
5	-0.515	-0.526	-0.684	-0.667	-0.478	-0.434	-0.27	-0.305
6	-0.511	-0.534	-0.671	-0.664	-0.477	-0.422	-0.259	-0.338
7	-0.509	-0.548	-0.646	-0.63	-0.497	-0.443	-0.256	-0.314
8	-0.535	-0.52	-0.647	-0.632	-0.5	-0.438	-0.255	-0.315
9	-0.497	-0.519	-0.652	-0.632	-0.463	-0.448	-0.275	-0.331
10	-0.517	-0.534	-0.651	-0.634	-0.466	-0.412	-0.271	-0.325
11	-0.544	-0.518	-0.653	-0.626	-0.458	-0.442	-0.267	-0.342
12	-0.491	-0.572	-0.651	-0.627	-0.46	-0.454	-0.263	-0.327
13	-0.539	-0.518	-0.659	-0.656	-0.5	-0.407	-0.295	-0.311
14	-0.491	-0.559	-0.661	-0.653	-0.5	-0.426	-0.281	-0.309
15	-0.515	-0.541	-0.684	-0.649	-0.493	-0.405	-0.277	-0.331
16	-0.534	-0.542	-0.674	-0.646	-0.499	-0.449	-0.28	-0.309
17	-0.488	-0.573	-0.686	-0.649	-0.491	-0.424	-0.292	-0.307
18	-0.521	-0.524	-0.668	-0.649	-0.483	-0.402	-0.291	-0.329
19	-0.515	-0.546	-0.67	-0.657	-0.497	-0.443	-0.284	-0.331
20	-0.508	-0.542	-0.673	-0.664	-0.518	-0.434	-0.287	-0.323
21	-0.512	-0.522	-0.67	-0.639	-0.479	-0.42	-0.275	-0.332
22	-0.526	-0.56	-0.669	-0.641	-0.479	-0.439	-0.286	-0.35
23	-0.527	-0.545	-0.671	-0.656	-0.478	-0.441	-0.271	-0.325
24	-0.521	-0.541	-0.669	-0.667	-0.497	-0.416	-0.296	-0.327
25	-0.509	-0.524	-0.672	-0.646	-0.47	-0.432	-0.3	-0.326
26	-0.516	-0.559	-0.671	-0.651	-0.471	-0.427	-0.292	-0.352
27	-0.516	-0.56	-0.672	-0.647	-0.513	-0.423	-0.306	-0.356
28	-0.547	-0.554	-0.666	-0.65	-0.506	-0.455	-0.307	-0.35
29	-0.541	-0.545	-0.671	-0.619	-0.491	-0.457	-0.283	-0.303
30	-0.495	-0.521	-0.667	-0.619	-0.505	-0.443	-0.279	-0.304
31	-0.493	-0.547	-0.633	-0.651	-0.498	-0.407	-0.299	-0.33
32	-0.53	-0.553	-0.629	-0.65	-0.503	-0.405	-0.293	-0.33
33	-0.532	-0.544	-0.635	-0.609	-0.468	-0.448	-0.276	-0.33
34	-0.508	-0.547	-0.631	-0.612	-0.468	-0.416	-0.272	-0.345
35	-0.532	-0.539	-0.674	-0.612	-0.498	-0.445	-0.307	-0.353
36	-0.527	-0.535	-0.67	-0.615	-0.502	-0.441	-0.303	-0.351
37	-0.54	-0.563	-0.64	-0.646	-0.501	-0.457	-0.289	-0.345
38	-0.528	-0.558	-0.64	-0.645	-0.509	-0.449	-0.288	-0.353
39	-0.535	-0.563	-0.674	-0.649	-0.493	-0.435	-0.3	-0.325
40	-0.542	-0.559	-0.669	-0.653	-0.455	-0.451	-0.294	-0.344
41	-0.526	-0.537	-0.666	-0.649	-0.457	-0.448	-0.307	-0.349
42	-0.542	-0.534	-0.665	-0.653	-0.492	-0.457	-0.308	-0.313
43	-0.536	-0.514	-0.647	-0.635	-0.497	-0.45	-0.294	-0.337
44	-0.535	-0.554	-0.647	-0.637	-0.493	-0.431	-0.296	-0.353
45	-0.526	-0.553	-0.647	-0.626	-0.49	-0.448	-0.3	-0.35
46	-0.523	-0.512	-0.648	-0.626	-0.484	-0.442	-0.291	-0.326
47	-0.517	-0.544	-0.658	-0.637	-0.49	-0.422	-0.291	-0.31
48	-0.514	-0.538	-0.658	-0.628	-0.478	-0.42	-0.3	-0.327
49	-0.527	-0.537	-0.66	-0.627	-0.484	-0.433	-0.303	-0.329

Supplementary Table 6. Formation enthalpies (H_f) with links to AFLOW.org entry pages for each calculated configuration. Units: H_f in eV/atom.

conf.	$HfNbTaTiWC_5$	$HfNbTaVZrC_5$	$HfMoNbTiZrC_5$	$HfMoTaTiVC_5$	$HfNbTaVWC_5$	$MoTaTiVWC_5$	$HfMoTaTiZrC_5$	$MoNbTaWZrC_5$
	H_f	H_f	H_f	H_f	H_f	H_f	H_f	H_f
1	-0.558	-0.644	-0.599	-0.489	-0.431	-0.277	-0.615	-0.299
2	-0.578	-0.625	-0.598	-0.509	-0.445	-0.274	-0.585	-0.291
3	-0.535	-0.644	-0.573	-0.518	-0.427	-0.265	-0.619	-0.268
4	-0.554	-0.625	-0.597	-0.53	-0.431	-0.27	-0.586	-0.264
5	-0.547	-0.635	-0.574	-0.478	-0.437	-0.28	-0.617	-0.306
6	-0.546	-0.635	-0.601	-0.48	-0.423	-0.277	-0.616	-0.308
7	-0.546	-0.635	-0.574	-0.496	-0.414	-0.271	-0.582	-0.3
8	-0.554	-0.635	-0.571	-0.496	-0.422	-0.278	-0.57	-0.274
9	-0.568	-0.596	-0.574	-0.514	-0.449	-0.268	-0.586	-0.274
10	-0.565	-0.589	-0.555	-0.506	-0.436	-0.267	-0.57	-0.312
11	-0.562	-0.599	-0.602	-0.474	-0.443	-0.277	-0.588	-0.271
12	-0.52	-0.64	-0.576	-0.47	-0.39	-0.28	-0.587	-0.293
13	-0.579	-0.59	-0.556	-0.517	-0.445	-0.288	-0.594	-0.291
14	-0.522	-0.62	-0.562	-0.514	-0.392	-0.3	-0.59	-0.301
15	-0.537	-0.641	-0.604	-0.505	-0.406	-0.297	-0.619	-0.269
16	-0.53	-0.62	-0.566	-0.497	-0.4	-0.297	-0.586	-0.292
17	-0.533	-0.607	-0.576	-0.517	-0.423	-0.296	-0.622	-0.269
18	-0.553	-0.578	-0.575	-0.511	-0.441	-0.303	-0.588	-0.292
19	-0.544	-0.606	-0.549	-0.489	-0.412	-0.29	-0.557	-0.265
20	-0.539	-0.577	-0.553	-0.49	-0.407	-0.287	-0.554	-0.293
21	-0.552	-0.594	-0.557	-0.488	-0.419	-0.291	-0.601	-0.294
22	-0.555	-0.586	-0.56	-0.505	-0.423	-0.294	-0.602	-0.263
23	-0.565	-0.596	-0.59	-0.493	-0.438	-0.298	-0.603	-0.264
24	-0.568	-0.609	-0.591	-0.493	-0.442	-0.294	-0.578	-0.296
25	-0.523	-0.589	-0.586	-0.51	-0.39	-0.318	-0.601	-0.291
26	-0.55	-0.61	-0.543	-0.533	-0.415	-0.319	-0.564	-0.29
27	-0.531	-0.613	-0.541	-0.508	-0.399	-0.289	-0.58	-0.315
28	-0.546	-0.612	-0.586	-0.524	-0.412	-0.286	-0.611	-0.317
29	-0.561	-0.628	-0.582	-0.525	-0.431	-0.305	-0.609	-0.314
30	-0.574	-0.624	-0.597	-0.533	-0.449	-0.314	-0.569	-0.321
31	-0.586	-0.627	-0.597	-0.516	-0.453	-0.307	-0.615	-0.3
32	-0.556	-0.593	-0.584	-0.513	-0.426	-0.311	-0.602	-0.316
33	-0.562	-0.618	-0.584	-0.473	-0.431	-0.303	-0.615	-0.319
34	-0.563	-0.624	-0.578	-0.521	-0.442	-0.302	-0.603	-0.294
35	-0.57	-0.592	-0.585	-0.468	-0.426	-0.315	-0.614	-0.295
36	-0.559	-0.617	-0.597	-0.516	-0.444	-0.319	-0.614	-0.289
37	-0.568	-0.617	-0.597	-0.51	-0.442	-0.306	-0.584	-0.311
38	-0.567	-0.624	-0.579	-0.511	-0.44	-0.303	-0.582	-0.317
39	-0.57	-0.617	-0.572	-0.51	-0.442	-0.323	-0.607	-0.318
40	-0.579	-0.624	-0.585	-0.513	-0.454	-0.318	-0.607	-0.309
41	-0.573	-0.61	-0.585	-0.488	-0.447	-0.313	-0.617	-0.315
42	-0.574	-0.623	-0.598	-0.517	-0.449	-0.307	-0.602	-0.273
43	-0.581	-0.618	-0.598	-0.487	-0.45	-0.295	-0.616	-0.291
44	-0.57	-0.624	-0.574	-0.523	-0.443	-0.291	-0.593	-0.297
45	-0.561	-0.617	-0.588	-0.499	-0.436	-0.313	-0.602	-0.319
46	-0.57	-0.609	-0.588	-0.491	-0.444	-0.31	-0.595	-0.316
47	-0.551	-0.612	-0.567	-0.507	-0.414	-0.312	-0.593	-0.27
48	-0.552	-0.612	-0.568	-0.509	-0.417	-0.306	-0.596	-0.291
49	-0.568	-0.611	-0.59	-0.503	-0.432	-0.306	-0.609	-0.294

Supplementary Table 7. Formation enthalpies (H_f) with links to AFLOW.org entry pages for each calculated configuration. Units: H_f in eV/atom.

conf.	$NbTaTiWZrC_5$	$HfNbTaWZrC_5$	$HfNbTiVWC_5$	$MoTaTiVZrC_5$	$MoNbTiVZrC_5$	$MoNbTaVZrC_5$	$HfTaTiVWC_5$	$NbTaVWZrC_5$
	H_f	H_f	H_f	H_f	H_f	H_f	H_f	H_f
1	-0.525	-0.56	-0.516	-0.451	-0.465	-0.415	-0.482	-0.396
2	-0.545	-0.562	-0.498	-0.473	-0.479	-0.406	-0.505	-0.414
3	-0.499	-0.516	-0.492	-0.483	-0.439	-0.403	-0.515	-0.392
4	-0.517	-0.525	-0.497	-0.493	-0.46	-0.398	-0.531	-0.403
5	-0.512	-0.504	-0.473	-0.431	-0.435	-0.383	-0.508	-0.396
6	-0.509	-0.517	-0.494	-0.433	-0.434	-0.379	-0.5	-0.387
7	-0.505	-0.524	-0.479	-0.453	-0.458	-0.404	-0.47	-0.371
8	-0.513	-0.547	-0.476	-0.453	-0.464	-0.409	-0.474	-0.38
9	-0.536	-0.503	-0.453	-0.477	-0.417	-0.367	-0.494	-0.417
10	-0.476	-0.555	-0.453	-0.467	-0.419	-0.407	-0.496	-0.344
11	-0.532	-0.511	-0.458	-0.428	-0.412	-0.414	-0.466	-0.41
12	-0.529	-0.538	-0.461	-0.425	-0.413	-0.362	-0.463	-0.402
13	-0.479	-0.541	-0.5	-0.482	-0.459	-0.421	-0.518	-0.346
14	-0.546	-0.512	-0.504	-0.473	-0.464	-0.359	-0.512	-0.412
15	-0.493	-0.545	-0.495	-0.463	-0.453	-0.383	-0.498	-0.36
16	-0.487	-0.554	-0.52	-0.453	-0.46	-0.415	-0.506	-0.354
17	-0.498	-0.575	-0.482	-0.477	-0.448	-0.357	-0.521	-0.388
18	-0.519	-0.537	-0.48	-0.47	-0.439	-0.409	-0.516	-0.407
19	-0.503	-0.51	-0.498	-0.451	-0.459	-0.383	-0.482	-0.37
20	-0.499	-0.511	-0.502	-0.466	-0.481	-0.4	-0.499	-0.365
21	-0.511	-0.54	-0.485	-0.442	-0.437	-0.378	-0.493	-0.376
22	-0.515	-0.557	-0.493	-0.441	-0.436	-0.401	-0.493	-0.381
23	-0.527	-0.534	-0.493	-0.451	-0.44	-0.404	-0.508	-0.344
24	-0.479	-0.52	-0.473	-0.451	-0.457	-0.391	-0.535	-0.4
25	-0.53	-0.543	-0.522	-0.473	-0.475	-0.375	-0.511	-0.404
26	-0.511	-0.557	-0.514	-0.496	-0.468	-0.387	-0.525	-0.352
27	-0.487	-0.534	-0.503	-0.467	-0.423	-0.382	-0.492	-0.374
28	-0.507	-0.539	-0.51	-0.484	-0.424	-0.362	-0.532	-0.372
29	-0.522	-0.542	-0.496	-0.486	-0.45	-0.359	-0.492	-0.39
30	-0.518	-0.575	-0.508	-0.493	-0.466	-0.419	-0.539	-0.386
31	-0.538	-0.554	-0.468	-0.477	-0.459	-0.419	-0.522	-0.411
32	-0.55	-0.538	-0.471	-0.474	-0.466	-0.404	-0.517	-0.416
33	-0.527	-0.552	-0.475	-0.423	-0.422	-0.412	-0.465	-0.405
34	-0.535	-0.54	-0.474	-0.418	-0.422	-0.376	-0.465	-0.408
35	-0.531	-0.554	-0.508	-0.482	-0.458	-0.409	-0.522	-0.391
36	-0.523	-0.55	-0.519	-0.475	-0.463	-0.411	-0.518	-0.406
37	-0.531	-0.564	-0.454	-0.466	-0.462	-0.404	-0.513	-0.404
38	-0.521	-0.54	-0.451	-0.469	-0.472	-0.419	-0.517	-0.388
39	-0.544	-0.504	-0.502	-0.472	-0.406	-0.412	-0.487	-0.418
40	-0.538	-0.542	-0.505	-0.474	-0.407	-0.414	-0.489	-0.41
41	-0.532	-0.535	-0.502	-0.477	-0.458	-0.396	-0.521	-0.404
42	-0.538	-0.55	-0.498	-0.485	-0.455	-0.419	-0.531	-0.412
43	-0.545	-0.563	-0.491	-0.442	-0.451	-0.412	-0.518	-0.413
44	-0.525	-0.551	-0.496	-0.441	-0.449	-0.392	-0.52	-0.399
45	-0.535	-0.519	-0.502	-0.454	-0.448	-0.412	-0.499	-0.408
46	-0.533	-0.536	-0.501	-0.446	-0.441	-0.405	-0.494	-0.406
47	-0.512	-0.505	-0.497	-0.464	-0.447	-0.381	-0.513	-0.374
48	-0.513	-0.534	-0.482	-0.468	-0.433	-0.38	-0.509	-0.376
49	-0.528	-0.535	-0.486	-0.46	-0.439	-0.391	-0.514	-0.391

Supplementary Table 8. Formation enthalpies (H_f) with links to AFLOW.org entry pages for each calculated configuration. Units: H_f in eV/atom.

conf.	$HfMoNbVWC_5$	$HfMoTaVWC_5$	$HfNbTiWZrC_5$	$HfMoTaTiWC_5$	$HfMoNbTiWC_5$	$HfTaTiWZrC_5$	$TaTiVWZrC_5$	$NbTiVWZrC_5$
	H_f	H_f	H_f	H_f	H_f	H_f	H_f	H_f
1	-0.275	-0.29	-0.601	-0.424	-0.394	-0.618	-0.443	-0.481
2	-0.292	-0.296	-0.602	-0.43	-0.418	-0.579	-0.469	-0.464
3	-0.286	-0.272	-0.571	-0.389	-0.416	-0.623	-0.482	-0.454
4	-0.281	-0.282	-0.605	-0.401	-0.415	-0.58	-0.496	-0.461
5	-0.278	-0.27	-0.57	-0.4	-0.397	-0.583	-0.472	-0.434
6	-0.263	-0.276	-0.6	-0.406	-0.379	-0.582	-0.461	-0.458
7	-0.294	-0.29	-0.573	-0.413	-0.42	-0.62	-0.427	-0.436
8	-0.256	-0.298	-0.608	-0.426	-0.381	-0.619	-0.424	-0.432
9	-0.25	-0.278	-0.571	-0.398	-0.378	-0.582	-0.45	-0.407
10	-0.274	-0.243	-0.605	-0.371	-0.394	-0.567	-0.452	-0.407
11	-0.264	-0.277	-0.575	-0.401	-0.396	-0.585	-0.419	-0.415
12	-0.26	-0.249	-0.575	-0.373	-0.389	-0.623	-0.418	-0.412
13	-0.271	-0.289	-0.572	-0.415	-0.382	-0.568	-0.455	-0.459
14	-0.266	-0.266	-0.573	-0.395	-0.368	-0.58	-0.464	-0.464
15	-0.244	-0.301	-0.544	-0.433	-0.386	-0.626	-0.482	-0.44
16	-0.267	-0.267	-0.556	-0.393	-0.376	-0.583	-0.472	-0.437
17	-0.274	-0.252	-0.554	-0.384	-0.397	-0.548	-0.443	-0.458
18	-0.262	-0.25	-0.547	-0.378	-0.39	-0.547	-0.46	-0.483
19	-0.233	-0.272	-0.564	-0.385	-0.359	-0.594	-0.48	-0.458
20	-0.252	-0.28	-0.559	-0.393	-0.381	-0.592	-0.475	-0.464
21	-0.235	-0.294	-0.556	-0.417	-0.363	-0.574	-0.45	-0.443
22	-0.229	-0.268	-0.567	-0.422	-0.404	-0.558	-0.452	-0.451
23	-0.277	-0.296	-0.536	-0.399	-0.403	-0.576	-0.472	-0.453
24	-0.274	-0.274	-0.536	-0.404	-0.355	-0.561	-0.498	-0.435
25	-0.245	-0.272	-0.599	-0.404	-0.411	-0.607	-0.486	-0.483
26	-0.24	-0.273	-0.599	-0.402	-0.372	-0.607	-0.47	-0.476
27	-0.287	-0.239	-0.582	-0.448	-0.371	-0.608	-0.493	-0.464
28	-0.284	-0.307	-0.578	-0.456	-0.407	-0.606	-0.5	-0.472
29	-0.267	-0.286	-0.582	-0.418	-0.396	-0.584	-0.445	-0.469
30	-0.282	-0.329	-0.593	-0.415	-0.41	-0.583	-0.445	-0.457
31	-0.301	-0.286	-0.58	-0.369	-0.437	-0.624	-0.483	-0.423
32	-0.303	-0.247	-0.606	-0.429	-0.425	-0.607	-0.477	-0.422
33	-0.313	-0.296	-0.607	-0.431	-0.438	-0.624	-0.415	-0.428
34	-0.298	-0.303	-0.59	-0.378	-0.431	-0.608	-0.415	-0.427
35	-0.263	-0.28	-0.593	-0.412	-0.393	-0.618	-0.483	-0.404
36	-0.278	-0.276	-0.609	-0.405	-0.407	-0.617	-0.477	-0.402
37	-0.291	-0.318	-0.593	-0.441	-0.42	-0.613	-0.474	-0.481
38	-0.305	-0.308	-0.592	-0.436	-0.438	-0.613	-0.478	-0.469
39	-0.283	-0.299	-0.594	-0.428	-0.413	-0.622	-0.441	-0.462
40	-0.287	-0.304	-0.607	-0.431	-0.419	-0.622	-0.44	-0.466
41	-0.261	-0.302	-0.608	-0.436	-0.416	-0.596	-0.492	-0.464
42	-0.292	-0.292	-0.591	-0.422	-0.393	-0.597	-0.482	-0.46
43	-0.291	-0.312	-0.592	-0.439	-0.416	-0.625	-0.474	-0.448
44	-0.293	-0.293	-0.607	-0.43	-0.421	-0.608	-0.477	-0.454
45	-0.283	-0.303	-0.597	-0.422	-0.411	-0.625	-0.455	-0.461
46	-0.257	-0.302	-0.597	-0.431	-0.389	-0.609	-0.45	-0.457
47	-0.269	-0.267	-0.571	-0.402	-0.398	-0.597	-0.468	-0.454
48	-0.27	-0.272	-0.57	-0.406	-0.4	-0.597	-0.465	-0.437
49	-0.277	-0.287	-0.596	-0.422	-0.41	-0.612	-0.472	-0.441

Supplementary Table 9. Formation enthalpies (H_f) with links to AFLOW.org entry pages for each calculated configuration. Units: H_f in eV/atom.

conf.	$HfMoTaVZrC_5$	$HfMoNbVZrC_5$	$HfMoTiVZrC_5$	$MoTaTiWZrC_5$	$MoNbTiWZrC_5$	$HfMoNbWZrC_5$	$MoNbVWZrC_5$	$MoTaVWZrC_5$
	H_f	H_f	H_f	H_f	H_f	H_f	H_f	H_f
1	-0.496	-0.476	-0.511	-0.391	-0.383	-0.34	-0.259	-0.257
2	-0.468	-0.473	-0.533	-0.398	-0.363	-0.386	-0.243	-0.265
3	-0.498	-0.456	-0.514	-0.352	-0.386	-0.372	-0.253	-0.235
4	-0.469	-0.479	-0.533	-0.365	-0.381	-0.339	-0.247	-0.246
5	-0.489	-0.457	-0.479	-0.358	-0.362	-0.376	-0.243	-0.227
6	-0.487	-0.481	-0.484	-0.363	-0.342	-0.339	-0.226	-0.232
7	-0.448	-0.44	-0.482	-0.383	-0.34	-0.387	-0.261	-0.259
8	-0.438	-0.438	-0.487	-0.326	-0.388	-0.339	-0.214	-0.242
9	-0.452	-0.475	-0.54	-0.361	-0.336	-0.372	-0.207	-0.196
10	-0.439	-0.499	-0.537	-0.393	-0.352	-0.412	-0.24	-0.265
11	-0.491	-0.424	-0.47	-0.364	-0.363	-0.35	-0.221	-0.241
12	-0.492	-0.475	-0.471	-0.329	-0.348	-0.35	-0.217	-0.202
13	-0.458	-0.499	-0.512	-0.383	-0.348	-0.37	-0.238	-0.257
14	-0.455	-0.426	-0.511	-0.352	-0.323	-0.412	-0.231	-0.222
15	-0.513	-0.43	-0.517	-0.338	-0.342	-0.37	-0.197	-0.269
16	-0.484	-0.434	-0.505	-0.352	-0.361	-0.377	-0.238	-0.205
17	-0.514	-0.478	-0.518	-0.4	-0.349	-0.367	-0.23	-0.224
18	-0.484	-0.479	-0.508	-0.333	-0.315	-0.37	-0.186	-0.203
19	-0.424	-0.417	-0.514	-0.351	-0.348	-0.377	-0.219	-0.238
20	-0.423	-0.421	-0.534	-0.36	-0.339	-0.371	-0.208	-0.247
21	-0.472	-0.424	-0.494	-0.379	-0.318	-0.333	-0.188	-0.256
22	-0.472	-0.428	-0.494	-0.383	-0.311	-0.332	-0.182	-0.257
23	-0.441	-0.467	-0.514	-0.365	-0.327	-0.402	-0.198	-0.224
24	-0.476	-0.468	-0.538	-0.357	-0.326	-0.393	-0.193	-0.231
25	-0.473	-0.411	-0.473	-0.362	-0.366	-0.401	-0.24	-0.232
26	-0.429	-0.411	-0.473	-0.362	-0.365	-0.386	-0.235	-0.231
27	-0.444	-0.458	-0.513	-0.324	-0.372	-0.395	-0.248	-0.192
28	-0.433	-0.46	-0.526	-0.411	-0.369	-0.407	-0.246	-0.276
29	-0.486	-0.455	-0.515	-0.418	-0.355	-0.406	-0.226	-0.2
30	-0.484	-0.468	-0.526	-0.39	-0.354	-0.407	-0.222	-0.292
31	-0.487	-0.466	-0.524	-0.333	-0.402	-0.345	-0.267	-0.259
32	-0.478	-0.459	-0.524	-0.379	-0.39	-0.394	-0.268	-0.247
33	-0.486	-0.444	-0.511	-0.377	-0.402	-0.392	-0.277	-0.249
34	-0.476	-0.461	-0.51	-0.393	-0.394	-0.374	-0.244	-0.239
35	-0.484	-0.46	-0.461	-0.372	-0.372	-0.378	-0.262	-0.266
36	-0.483	-0.468	-0.464	-0.366	-0.383	-0.365	-0.256	-0.235
37	-0.445	-0.468	-0.51	-0.405	-0.374	-0.408	-0.246	-0.283
38	-0.443	-0.445	-0.512	-0.389	-0.369	-0.375	-0.239	-0.272
39	-0.489	-0.44	-0.466	-0.398	-0.404	-0.402	-0.27	-0.261
40	-0.488	-0.466	-0.467	-0.394	-0.383	-0.387	-0.25	-0.269
41	-0.495	-0.466	-0.517	-0.399	-0.353	-0.398	-0.22	-0.264
42	-0.481	-0.475	-0.527	-0.402	-0.381	-0.403	-0.256	-0.276
43	-0.494	-0.442	-0.518	-0.394	-0.38	-0.343	-0.254	-0.266
44	-0.458	-0.475	-0.527	-0.384	-0.384	-0.345	-0.216	-0.253
45	-0.481	-0.469	-0.508	-0.394	-0.373	-0.399	-0.257	-0.266
46	-0.457	-0.47	-0.496	-0.384	-0.35	-0.366	-0.245	-0.255
47	-0.459	-0.429	-0.489	-0.363	-0.358	-0.375	-0.228	-0.226
48	-0.459	-0.43	-0.499	-0.366	-0.36	-0.342	-0.228	-0.23
49	-0.467	-0.45	-0.491	-0.385	-0.372	-0.374	-0.239	-0.25

Supplementary Table 10. Formation enthalpies (H_f) with links to AFLOW.org entry pages for each calculated configuration. Units: H_f in eV/atom.

conf.	$HfTiVWZrC_5$	$HfNbVWZrC_5$	$HfMoTiVWC_5$	$HfMoTaWZrC_5$	$HfTaVWZrC_5$	$MoTiVWZrC_5$	$HfMoTiWZrC_5$	$HfMoVWZrC_5$
	H_f	H_f	H_f	H_f	H_f	H_f	H_f	H_f
1	-0.511	-0.47	-0.374	-0.403	-0.495	-0.339	-0.472	-0.336
2	-0.536	-0.474	-0.371	-0.379	-0.46	-0.336	-0.472	-0.337
3	-0.513	-0.471	-0.336	-0.405	-0.499	-0.298	-0.416	-0.312
4	-0.537	-0.501	-0.343	-0.358	-0.459	-0.307	-0.473	-0.354
5	-0.544	-0.451	-0.34	-0.344	-0.488	-0.295	-0.421	-0.316
6	-0.541	-0.478	-0.337	-0.376	-0.488	-0.292	-0.477	-0.356
7	-0.489	-0.472	-0.333	-0.357	-0.488	-0.294	-0.427	-0.292
8	-0.483	-0.501	-0.344	-0.418	-0.485	-0.307	-0.425	-0.291
9	-0.485	-0.451	-0.309	-0.388	-0.443	-0.261	-0.417	-0.31
10	-0.49	-0.483	-0.311	-0.417	-0.43	-0.262	-0.392	-0.261
11	-0.472	-0.439	-0.309	-0.355	-0.515	-0.265	-0.473	-0.349
12	-0.473	-0.44	-0.314	-0.343	-0.447	-0.261	-0.406	-0.277
13	-0.516	-0.476	-0.361	-0.383	-0.478	-0.323	-0.421	-0.312
14	-0.528	-0.477	-0.376	-0.354	-0.432	-0.34	-0.394	-0.264
15	-0.512	-0.412	-0.371	-0.424	-0.517	-0.33	-0.409	-0.352
16	-0.511	-0.422	-0.369	-0.352	-0.479	-0.328	-0.477	-0.281
17	-0.518	-0.418	-0.347	-0.361	-0.413	-0.304	-0.417	-0.319
18	-0.527	-0.415	-0.344	-0.351	-0.413	-0.3	-0.418	-0.321
19	-0.502	-0.427	-0.362	-0.389	-0.456	-0.323	-0.394	-0.26
20	-0.503	-0.426	-0.374	-0.386	-0.455	-0.336	-0.41	-0.277
21	-0.514	-0.42	-0.346	-0.391	-0.437	-0.302	-0.398	-0.264
22	-0.537	-0.431	-0.349	-0.425	-0.422	-0.306	-0.412	-0.281
23	-0.515	-0.403	-0.341	-0.388	-0.441	-0.3	-0.452	-0.331
24	-0.542	-0.402	-0.337	-0.376	-0.425	-0.295	-0.451	-0.329
25	-0.539	-0.474	-0.331	-0.345	-0.475	-0.286	-0.441	-0.249
26	-0.525	-0.473	-0.335	-0.385	-0.473	-0.282	-0.437	-0.307
27	-0.527	-0.445	-0.401	-0.375	-0.478	-0.364	-0.38	-0.248
28	-0.539	-0.444	-0.402	-0.391	-0.474	-0.364	-0.379	-0.308
29	-0.537	-0.446	-0.369	-0.361	-0.442	-0.33	-0.44	-0.308
30	-0.537	-0.447	-0.383	-0.414	-0.441	-0.344	-0.439	-0.313
31	-0.48	-0.461	-0.37	-0.409	-0.491	-0.338	-0.476	-0.351
32	-0.48	-0.471	-0.376	-0.344	-0.478	-0.33	-0.476	-0.35
33	-0.464	-0.473	-0.335	-0.415	-0.491	-0.288	-0.448	-0.333
34	-0.467	-0.46	-0.337	-0.388	-0.476	-0.289	-0.476	-0.346
35	-0.469	-0.471	-0.386	-0.42	-0.49	-0.352	-0.45	-0.325
36	-0.467	-0.463	-0.39	-0.406	-0.49	-0.345	-0.476	-0.349
37	-0.522	-0.482	-0.384	-0.382	-0.489	-0.344	-0.444	-0.31
38	-0.521	-0.471	-0.383	-0.419	-0.489	-0.345	-0.439	-0.309
39	-0.53	-0.464	-0.36	-0.388	-0.487	-0.26	-0.446	-0.326
40	-0.542	-0.482	-0.363	-0.41	-0.485	-0.255	-0.478	-0.349
41	-0.541	-0.474	-0.366	-0.379	-0.46	-0.316	-0.448	-0.328
42	-0.53	-0.463	-0.311	-0.424	-0.457	-0.32	-0.477	-0.351
43	-0.523	-0.462	-0.364	-0.408	-0.499	-0.327	-0.444	-0.312
44	-0.525	-0.475	-0.307	-0.38	-0.483	-0.322	-0.441	-0.312
45	-0.499	-0.474	-0.366	-0.411	-0.499	-0.324	-0.456	-0.339
46	-0.505	-0.473	-0.361	-0.378	-0.483	-0.317	-0.456	-0.34
47	-0.521	-0.431	-0.363	-0.424	-0.462	-0.321	-0.426	-0.287
48	-0.5	-0.43	-0.349	-0.41	-0.459	-0.304	-0.424	-0.287
49	-0.508	-0.454	-0.348	-0.392	-0.468	-0.303	-0.449	-0.311

SUPPLEMENTARY NOTE 7: GEOMETRY INPUT FILES

The PARTCAR (the geometry input file for the AFLOW partial occupation (AFLOW-POCC) algorithm [6]) and initial POSCARs (the VASP [7] input file for the atomic geometry) for all 49 configurations of HfNbTaTiZrC$_5$ are presented below. Starting with the rock-salt crystal structure (spacegroup: $Fm\overline{3}m$, #225; Pearson symbol: cF8; AFLOW Prototype: AB_cF8_225_a_b [8]) as the input parent lattice, the AFLOW-POCC algorithm generates a set of 49 distinct configurations, each containing one atom of each of the 5 metals, along with 5 carbon atoms. This is the minimum cell size necessary to accurately reproduce the required stoichiometry: C atom with full occupancy at the anionic lattice site and 5 different refractory metal elements with a 0.2 occupancy probability for each at the cationic lattice site. The degeneracy for each configuration g_i is given by DG in the header of each POSCAR. For HfNbTaTiZrC$_5$, all configurations have $g_i = 10$, except for the 49th where $g_i = 120$. Each of the other 5-metal carbides also have the same 49 distinct configurations, with the same number of anions and cations in the unit cell, but with a different set of 5 refractory metal elements at the cationic sites. Note that the numerical designation of the configurations can vary from system to system. The total degeneracy ($\sum_i g_i = 600$) is same for all 5-metal systems.

HfNbTaTiZrC$_5$: PARTCAR

```
CHf_pvNb_svTa_pvTi_svZr_sv:PAW_PBE.AB_cF8_225_a_b.POCC
1.224745   0.001
   0.000000000000    1.898165125850    1.898165125850
   1.898165125850    0.000000000000    1.898165125850
   1.898165125850    1.898165125850    0.000000000000
1*1.0  1*0.2  1*0.2  1*0.2  1*0.2  1*0.2
Direct(6)
   0.500000000000    0.500000000000    0.500000000000  C
   0.000000000000    0.000000000000    0.000000000000  Hf_pv
   0.000000000000    0.000000000000    0.000000000000  Nb_sv
   0.000000000000    0.000000000000    0.000000000000  Ta_pv
   0.000000000000    0.000000000000    0.000000000000  Ti_sv
   0.000000000000    0.000000000000    0.000000000000  Zr_sv
```

HfNbTaTiZrC$_5$:configuration 1 - POSCAR

```
CHf_pvNb_svTa_pvTi_svZr_sv.AB_cF8_225_a_b.POCC   [HNF(5)=1/5= 1
    ↪ 0 0; 0 1 0; 0 0 5] DG=10   [RHL,RHL1,hR10]  (STD_PRIM doi
    ↪ :10.1016/j.commatsci.2010.05.010) [Standard_Primitive
    ↪ Unit Cell Form]
1.000000
   13.455569032370   -1.643859392183    0.000000000000
   13.455569032370    1.643859392183    0.000000000000
   13.254739643828    0.000000000000    2.840156450004
5 1 1 1 1 1
Direct(10) [A5B1C1D1E1F1]
   0.300000000000    0.300000000000    0.300000000000   C
   0.900000000000    0.900000000000    0.900000000000   C
   0.500000000000    0.500000000000    0.500000000000   C
   0.100000000000    0.100000000000    0.100000000000   C
   0.700000000000    0.700000000000    0.700000000000   C
   0.400000000000    0.400000000000    0.400000000000   Hf_pv
   0.000000000000    0.000000000000    0.000000000000   Nb_sv
   0.800000000000    0.800000000000    0.800000000000   Ta_pv
   0.600000000000    0.600000000000    0.600000000000   Ti_sv
   0.200000000000    0.200000000000    0.200000000000   Zr_sv
```

HfNbTaTiZrC$_5$:configuration 2 - POSCAR

```
CHf_pvNb_svTa_pvTi_svZr_sv.AB_cF8_225_a_b.POCC   [HNF(5)=1/5= 1
    ↪ 0 0; 0 1 0; 0 0 5] DG=10   [RHL,RHL1,hR10]  (STD_PRIM doi
    ↪ :10.1016/j.commatsci.2010.05.010) [Standard_Primitive
    ↪ Unit Cell Form]
1.000000
   13.455569032370   -1.643859392183    0.000000000000
   13.455569032370    1.643859392183    0.000000000000
   13.254739643828    0.000000000000    2.840156450004
5 1 1 1 1 1
Direct(10) [A5B1C1D1E1F1]
   0.300000000000    0.300000000000    0.300000000000   C
   0.900000000000    0.900000000000    0.900000000000   C
   0.500000000000    0.500000000000    0.500000000000   C
   0.100000000000    0.100000000000    0.100000000000   C
   0.700000000000    0.700000000000    0.700000000000   C
   0.400000000000    0.400000000000    0.400000000000   Hf_pv
   0.600000000000    0.600000000000    0.600000000000   Nb_sv
   0.000000000000    0.000000000000    0.000000000000   Ta_pv
   0.200000000000    0.200000000000    0.200000000000   Ti_sv
   0.800000000000    0.800000000000    0.800000000000   Zr_sv
```

HfNbTaTiZrC$_5$:configuration 3 - POSCAR

```
CHf_pvNb_svTa_pvTi_svZr_sv.AB_cF8_225_a_b.POCC   [HNF(5)=1/5= 1
    ↪ 0 0; 0 1 0; 0 0 5] DG=10   [RHL,RHL1,hR10]  (STD_PRIM doi
    ↪ :10.1016/j.commatsci.2010.05.010) [Standard_Primitive
    ↪ Unit Cell Form]
1.000000
   13.455569032370   -1.643859392183    0.000000000000
   13.455569032370    1.643859392183    0.000000000000
   13.254739643827    0.000000000000    2.840156450004
5 1 1 1 1 1
Direct(10) [A5B1C1D1E1F1]
   0.300000000000    0.300000000000    0.300000000000   C
   0.900000000000    0.900000000000    0.900000000000   C
   0.500000000000    0.500000000000    0.500000000000   C
   0.100000000000    0.100000000000    0.100000000000   C
   0.700000000000    0.700000000000    0.700000000000   C
   0.400000000000    0.400000000000    0.400000000000   Hf_pv
   0.600000000000    0.600000000000    0.600000000000   Nb_sv
   0.800000000000    0.800000000000    0.800000000000   Ta_pv
   0.000000000000    0.000000000000    0.000000000000   Ti_sv
   0.200000000000    0.200000000000    0.200000000000   Zr_sv
```

HfNbTaTiZrC$_5$:configuration 4 - POSCAR

```
CHf_pvNb_svTa_pvTi_svZr_sv.AB_cF8_225_a_b.POCC   [HNF(5)=1/5= 1
    ↪ 0 0; 0 1 0; 0 0 5] DG=10   [RHL,RHL1,hR10]  (STD_PRIM doi
    ↪ :10.1016/j.commatsci.2010.05.010) [Standard_Primitive
    ↪ Unit Cell Form]
1.000000
   13.455569032370   -1.643859392183    0.000000000000
   13.455569032370    1.643859392183    0.000000000000
   13.254739643829    0.000000000000    2.840156450004
5 1 1 1 1 1
Direct(10) [A5B1C1D1E1F1]
   0.300000000000    0.300000000000    0.300000000000   C
   0.900000000000    0.900000000000    0.900000000000   C
   0.500000000000    0.500000000000    0.500000000000   C
   0.100000000000    0.100000000000    0.100000000000   C
   0.700000000000    0.700000000000    0.700000000000   C
   0.400000000000    0.400000000000    0.400000000000   Hf_pv
   0.600000000000    0.600000000000    0.600000000000   Nb_sv
   0.200000000000    0.200000000000    0.200000000000   Ta_pv
   0.000000000000    0.000000000000    0.000000000000   Ti_sv
   0.800000000000    0.800000000000    0.800000000000   Zr_sv
```

HfNbTaTiZrC$_5$:configuration 5 - POSCAR

```
CHf_pvNb_svTa_pvTi_svZr_sv.AB_cF8_225_a_b.POCC   [HNF(5)=1/5= 1
    ↪ 0 0; 0 1 0; 0 0 5] DG=10   [RHL,RHL1,hR10]  (STD_PRIM doi
    ↪ :10.1016/j.commatsci.2010.05.010) [Standard_Primitive
    ↪ Unit Cell Form]
1.000000
   13.455569032370   -1.643859392183    0.000000000000
   13.455569032370    1.643859392183    0.000000000000
   13.254739643828    0.000000000000    2.840156450004
5 1 1 1 1 1
Direct(10) [A5B1C1D1E1F1]
   0.300000000000    0.300000000000    0.300000000000   C
   0.900000000000    0.900000000000    0.900000000000   C
   0.500000000000    0.500000000000    0.500000000000   C
   0.100000000000    0.100000000000    0.100000000000   C
   0.700000000000    0.700000000000    0.700000000000   C
   0.400000000000    0.400000000000    0.400000000000   Hf_pv
   0.600000000000    0.600000000000    0.600000000000   Nb_sv
   0.000000000000    0.000000000000    0.000000000000   Ta_pv
   0.800000000000    0.800000000000    0.800000000000   Ti_sv
   0.200000000000    0.200000000000    0.200000000000   Zr_sv
```

HfNbTaTiZrC$_5$:configuration 6 - POSCAR

```
CHf_pvNb_svTa_pvTi_svZr_sv.AB_cF8_225_a_b.POCC   [HNF(5)=1/5= 1
    ↪ 0 0; 0 1 0; 0 0 5] DG=10   [RHL,RHL1,hR10]  (STD_PRIM doi
    ↪ :10.1016/j.commatsci.2010.05.010) [Standard_Primitive
    ↪ Unit Cell Form]
1.000000
   13.455569032370   -1.643859392183    0.000000000000
   13.455569032370    1.643859392183    0.000000000000
   13.254739643828    0.000000000000    2.840156450004
5 1 1 1 1 1
Direct(10) [A5B1C1D1E1F1]
   0.300000000000    0.300000000000    0.300000000000   C
   0.900000000000    0.900000000000    0.900000000000   C
   0.500000000000    0.500000000000    0.500000000000   C
   0.100000000000    0.100000000000    0.100000000000   C
   0.700000000000    0.700000000000    0.700000000000   C
   0.400000000000    0.400000000000    0.400000000000   Hf_pv
   0.600000000000    0.600000000000    0.600000000000   Nb_sv
   0.800000000000    0.800000000000    0.800000000000   Ta_pv
   0.200000000000    0.200000000000    0.200000000000   Ti_sv
   0.000000000000    0.000000000000    0.000000000000   Zr_sv
```

HfNbTaTiZrC$_5$:configuration 7 - POSCAR

```
CHf_pvNb_svTa_pvTi_svZr_sv.AB_cF8_225_a_b.POCC   [HNF(5)=1/5= 1
    ↪ 0 0; 0 1 0; 0 0 5] DG=10   [RHL,RHL1,hR10]  (STD_PRIM doi
    ↪ :10.1016/j.commatsci.2010.05.010) [Standard_Primitive
    ↪ Unit Cell Form]
1.000000
   13.455569032370   -1.643859392183    0.000000000000
   13.455569032370    1.643859392183    0.000000000000
```

```
   13.254739643828    0.000000000000    2.840156450004
5 1 1 1 1 1
Direct(10) [A5B1C1D1E1F1]
   0.300000000000    0.300000000000    0.300000000000   C
   0.900000000000    0.900000000000    0.900000000000   C
   0.500000000000    0.500000000000    0.500000000000   C
   0.100000000000    0.100000000000    0.100000000000   C
   0.700000000000    0.700000000000    0.700000000000   C
   0.400000000000    0.400000000000    0.400000000000   Hf_pv
   0.000000000000    0.000000000000    0.000000000000   Nb_sv
   0.600000000000    0.600000000000    0.600000000000   Ta_pv
   0.800000000000    0.800000000000    0.800000000000   Ti_sv
   0.200000000000    0.200000000000    0.200000000000   Zr_sv
```

HfNbTaTiZrC$_5$:configuration 8 - POSCAR

```
CHf_pvNb_svTa_pvTi_svZr_sv.AB_cF8_225_a_b.POCC   [HNF(5)=1/5= 1
     ↪ 0 0; 0 1 0; 0 0 5] DG=10   [RHL,RHL1,hR10]  (STD_PRIM doi
     ↪ :10.1016/j.commatsci.2010.05.010) [Standard_Primitive
     ↪ Unit Cell Form]
1.000000
   13.455569032370   -1.643859392183    0.000000000000
   13.455569032370    1.643859392183    0.000000000000
   13.254739643828    0.000000000000    2.840156450004
5 1 1 1 1 1
Direct(10) [A5B1C1D1E1F1]
   0.300000000000    0.300000000000    0.300000000000   C
   0.900000000000    0.900000000000    0.900000000000   C
   0.500000000000    0.500000000000    0.500000000000   C
   0.100000000000    0.100000000000    0.100000000000   C
   0.700000000000    0.700000000000    0.700000000000   C
   0.400000000000    0.400000000000    0.400000000000   Hf_pv
   0.000000000000    0.000000000000    0.000000000000   Nb_sv
   0.200000000000    0.200000000000    0.200000000000   Ta_pv
   0.600000000000    0.600000000000    0.600000000000   Ti_sv
   0.800000000000    0.800000000000    0.800000000000   Zr_sv
```

HfNbTaTiZrC$_5$:configuration 9 - POSCAR

```
CHf_pvNb_svTa_pvTi_svZr_sv.AB_cF8_225_a_b.POCC   [HNF(5)=4/5= 1
     ↪ 0 0; 0 1 0; 0 4 5] DG=10   [BCT,BCT2,tI20]  (STD_PRIM doi
     ↪ :10.1016/j.commatsci.2010.05.010) [Standard_Primitive
     ↪ Unit Cell Form]
1.000000
  -1.643859392183    1.643859392183    11.623841235295
   1.643859392183   -1.643859392183    11.623841235295
   1.643859392183    1.643859392183   -11.623841235295
5 1 1 1 1 1
Direct(10) [A5B1C1D1E1F1]
   0.300000000000    0.300000000000   -0.000000000000   C
   0.900000000000    0.900000000000   -0.000000000000   C
   0.500000000000    0.500000000000   -0.000000000000   C
   0.100000000000    0.100000000000   -0.000000000000   C
   0.700000000000    0.700000000000   -0.000000000000   C
   0.400000000000    0.400000000000    0.000000000000   Hf_pv
   0.000000000000    0.000000000000    0.000000000000   Nb_sv
   0.800000000000    0.800000000000    0.000000000000   Ta_pv
   0.600000000000    0.600000000000    0.000000000000   Ti_sv
   0.200000000000    0.200000000000    0.000000000000   Zr_sv
```

HfNbTaTiZrC$_5$:configuration 10 - POSCAR

```
CHf_pvNb_svTa_pvTi_svZr_sv.AB_cF8_225_a_b.POCC   [HNF(5)=4/5= 1
     ↪ 0 0; 0 1 0; 0 4 5] DG=10   [BCT,BCT2,tI20]  (STD_PRIM doi
     ↪ :10.1016/j.commatsci.2010.05.010) [Standard_Primitive
     ↪ Unit Cell Form]
1.000000
  -1.643859392183    1.643859392183    11.623841235295
   1.643859392183   -1.643859392183    11.623841235295
   1.643859392183    1.643859392183   -11.623841235295
5 1 1 1 1 1
Direct(10) [A5B1C1D1E1F1]
   0.300000000000    0.300000000000   -0.000000000000   C
   0.900000000000    0.900000000000   -0.000000000000   C
   0.500000000000    0.500000000000   -0.000000000000   C
   0.100000000000    0.100000000000   -0.000000000000   C
   0.700000000000    0.700000000000   -0.000000000000   C
   0.400000000000    0.400000000000    0.000000000000   Hf_pv
   0.600000000000    0.600000000000    0.000000000000   Nb_sv
   0.000000000000    0.000000000000    0.000000000000   Ta_pv
   0.200000000000    0.200000000000    0.000000000000   Ti_sv
   0.800000000000    0.800000000000    0.000000000000   Zr_sv
```

HfNbTaTiZrC$_5$:configuration 11 - POSCAR

```
CHf_pvNb_svTa_pvTi_svZr_sv.AB_cF8_225_a_b.POCC   [HNF(5)=4/5= 1
     ↪ 0 0; 0 1 0; 0 4 5] DG=10   [BCT,BCT2,tI20]  (STD_PRIM doi
     ↪ :10.1016/j.commatsci.2010.05.010) [Standard_Primitive
     ↪ Unit Cell Form]
1.000000
  -1.643859392183    1.643859392183    11.623841235295
   1.643859392183   -1.643859392183    11.623841235295
   1.643859392183    1.643859392183   -11.623841235295
5 1 1 1 1 1
Direct(10) [A5B1C1D1E1F1]
   0.300000000000    0.300000000000   -0.000000000000   C
   0.900000000000    0.900000000000   -0.000000000000   C
   0.500000000000    0.500000000000   -0.000000000000   C
   0.100000000000    0.100000000000   -0.000000000000   C
   0.700000000000    0.700000000000   -0.000000000000   C
   0.400000000000    0.400000000000    0.000000000000   Hf_pv
   0.600000000000    0.600000000000    0.000000000000   Nb_sv
   0.800000000000    0.800000000000    0.000000000000   Ta_pv
   0.000000000000    0.000000000000    0.000000000000   Ti_sv
   0.200000000000    0.200000000000    0.000000000000   Zr_sv
```

HfNbTaTiZrC$_5$:configuration 12 - POSCAR

```
CHf_pvNb_svTa_pvTi_svZr_sv.AB_cF8_225_a_b.POCC   [HNF(5)=4/5= 1
     ↪ 0 0; 0 1 0; 0 4 5] DG=10   [BCT,BCT2,tI20]  (STD_PRIM doi
     ↪ :10.1016/j.commatsci.2010.05.010) [Standard_Primitive
     ↪ Unit Cell Form]
1.000000
  -1.643859392183    1.643859392183    11.623841235295
   1.643859392183   -1.643859392183    11.623841235295
   1.643859392183    1.643859392183   -11.623841235295
5 1 1 1 1 1
Direct(10) [A5B1C1D1E1F1]
   0.300000000000    0.300000000000   -0.000000000000   C
   0.900000000000    0.900000000000   -0.000000000000   C
   0.500000000000    0.500000000000   -0.000000000000   C
   0.100000000000    0.100000000000   -0.000000000000   C
   0.700000000000    0.700000000000   -0.000000000000   C
   0.400000000000    0.400000000000    0.000000000000   Hf_pv
   0.600000000000    0.600000000000    0.000000000000   Nb_sv
   0.200000000000    0.200000000000    0.000000000000   Ta_pv
   0.000000000000    0.000000000000    0.000000000000   Ti_sv
   0.800000000000    0.800000000000    0.000000000000   Zr_sv
```

HfNbTaTiZrC$_5$:configuration 13 - POSCAR

```
CHf_pvNb_svTa_pvTi_svZr_sv.AB_cF8_225_a_b.POCC   [HNF(5)=1/5= 1
     ↪ 0 0; 0 1 0; 0 0 5] DG=10   [RHL,RHL1,hR10]  (STD_PRIM doi
     ↪ :10.1016/j.commatsci.2010.05.010) [Standard_Primitive
     ↪ Unit Cell Form]
1.000000
   13.455569032370   -1.643859392183    0.000000000000
   13.455569032370    1.643859392183    0.000000000000
   13.254739643828    0.000000000000    2.840156450004
5 1 1 1 1 1
Direct(10) [A5B1C1D1E1F1]
   0.300000000000    0.300000000000    0.300000000000   C
   0.900000000000    0.900000000000    0.900000000000   C
   0.500000000000    0.500000000000    0.500000000000   C
   0.100000000000    0.100000000000    0.100000000000   C
   0.700000000000    0.700000000000    0.700000000000   C
   0.400000000000    0.400000000000    0.400000000000   Hf_pv
   0.000000000000    0.000000000000    0.000000000000   Nb_sv
   0.800000000000    0.800000000000    0.800000000000   Ta_pv
   0.200000000000    0.200000000000    0.200000000000   Ti_sv
   0.600000000000    0.600000000000    0.600000000000   Zr_sv
```

HfNbTaTiZrC$_5$:configuration 14 - POSCAR

```
CHf_pvNb_svTa_pvTi_svZr_sv.AB_cF8_225_a_b.POCC   [HNF(5)=1/5= 1
     ↪ 0 0; 0 1 0; 0 0 5] DG=10   [RHL,RHL1,hR10]  (STD_PRIM doi
     ↪ :10.1016/j.commatsci.2010.05.010) [Standard_Primitive
     ↪ Unit Cell Form]
1.000000
   13.455569032370   -1.643859392183    0.000000000000
   13.455569032370    1.643859392183    0.000000000000
   13.254739643828    0.000000000000    2.840156450004
5 1 1 1 1 1
Direct(10) [A5B1C1D1E1F1]
   0.300000000000    0.300000000000    0.300000000000   C
   0.900000000000    0.900000000000    0.900000000000   C
   0.500000000000    0.500000000000    0.500000000000   C
   0.100000000000    0.100000000000    0.100000000000   C
   0.700000000000    0.700000000000    0.700000000000   C
   0.400000000000    0.400000000000    0.400000000000   Hf_pv
   0.000000000000    0.000000000000    0.000000000000   Nb_sv
   0.600000000000    0.600000000000    0.600000000000   Ta_pv
   0.200000000000    0.200000000000    0.200000000000   Ti_sv
   0.800000000000    0.800000000000    0.800000000000   Zr_sv
```

HfNbTaTiZrC$_5$:configuration 15 - POSCAR

```
CHf_pvNb_svTa_pvTi_svZr_sv.AB_cF8_225_a_b.POCC   [HNF(5)=1/5= 1
     ↪ 0 0; 0 1 0; 0 0 5] DG=10   [RHL,RHL1,hR10]  (STD_PRIM doi
     ↪ :10.1016/j.commatsci.2010.05.010) [Standard_Primitive
     ↪ Unit Cell Form]
1.000000
   13.455569032370   -1.643859392183    0.000000000000
   13.455569032370    1.643859392183    0.000000000000
   13.254739643828    0.000000000000    2.840156450004
5 1 1 1 1 1
Direct(10) [A5B1C1D1E1F1]
   0.300000000000    0.300000000000    0.300000000000   C
   0.900000000000    0.900000000000    0.900000000000   C
   0.500000000000    0.500000000000    0.500000000000   C
   0.100000000000    0.100000000000    0.100000000000   C
   0.700000000000    0.700000000000    0.700000000000   C
   0.400000000000    0.400000000000    0.400000000000   Hf_pv
   0.000000000000    0.000000000000    0.000000000000   Nb_sv
   0.200000000000    0.200000000000    0.200000000000   Ta_pv
   0.800000000000    0.800000000000    0.800000000000   Ti_sv
   0.600000000000    0.600000000000    0.600000000000   Zr_sv
```

HfNbTaTiZrC$_5$:configuration 16 - POSCAR

```
CHf_pvNb_svTa_pvTi_svZr_sv.AB_cF8_225_a_b.POCC   [HNF(5)=1/5= 1
     ↪ 0 0; 0 1 0; 0 0 5] DG=10   [RHL,RHL1,hR10]  (STD_PRIM doi
     ↪ :10.1016/j.commatsci.2010.05.010) [Standard_Primitive
     ↪ Unit Cell Form]
1.000000
   13.455569032370   -1.643859392183    0.000000000000
   13.455569032370    1.643859392183    0.000000000000
   13.254739643828    0.000000000000    2.840156450004
5 1 1 1 1 1
Direct(10) [A5B1C1D1E1F1]
   0.300000000000    0.300000000000    0.300000000000   C
   0.900000000000    0.900000000000    0.900000000000   C
   0.500000000000    0.500000000000    0.500000000000   C
   0.100000000000    0.100000000000    0.100000000000   C
   0.700000000000    0.700000000000    0.700000000000   C
   0.400000000000    0.400000000000    0.400000000000   Hf_pv
   0.600000000000    0.600000000000    0.600000000000   Nb_sv
```

```
   0.200000000000    0.200000000000    0.200000000000   Ta_pv
   0.800000000000    0.800000000000    0.800000000000   Ti_sv
   0.000000000000    0.000000000000    0.000000000000   Zr_sv
```

HfNbTaTiZrC$_5$:configuration 17 - POSCAR

```
CHf_pvNb_svTa_pvTi_svZr_sv.AB_cF8_225_a_b.POCC   [HNF(5)=4/5= 1
     ↪ 0 0; 0 1 0; 0 4 5] DG=10   [BCT,BCT2,tI20] (STD_PRIM doi
     ↪ :10.1016/j.commatsci.2010.05.010) [Standard_Primitive
     ↪ Unit Cell Form]
1.000000
  -1.643859392183    1.643859392183    11.623841235295
   1.643859392183   -1.643859392183    11.623841235295
   1.643859392183    1.643859392183   -11.623841235295
5 1 1 1 1 1
Direct(10) [A5B1C1D1E1F1]
   0.300000000000    0.300000000000   -0.000000000000   C
   0.900000000000    0.900000000000   -0.000000000000   C
   0.500000000000    0.500000000000   -0.000000000000   C
   0.100000000000    0.100000000000   -0.000000000000   C
   0.700000000000    0.700000000000   -0.000000000000   C
   0.400000000000    0.400000000000    0.000000000000   Hf_pv
   0.600000000000    0.600000000000    0.000000000000   Nb_sv
   0.800000000000    0.800000000000    0.000000000000   Ta_pv
   0.200000000000    0.200000000000    0.000000000000   Ti_sv
   0.000000000000    0.000000000000    0.000000000000   Zr_sv
```

HfNbTaTiZrC$_5$:configuration 18 - POSCAR

```
CHf_pvNb_svTa_pvTi_svZr_sv.AB_cF8_225_a_b.POCC   [HNF(5)=4/5= 1
     ↪ 0 0; 0 1 0; 0 4 5] DG=10   [BCT,BCT2,tI20] (STD_PRIM doi
     ↪ :10.1016/j.commatsci.2010.05.010) [Standard_Primitive
     ↪ Unit Cell Form]
1.000000
  -1.643859392183    1.643859392183    11.623841235295
   1.643859392183   -1.643859392183    11.623841235295
   1.643859392183    1.643859392183   -11.623841235295
5 1 1 1 1 1
Direct(10) [A5B1C1D1E1F1]
   0.300000000000    0.300000000000   -0.000000000000   C
   0.900000000000    0.900000000000   -0.000000000000   C
   0.500000000000    0.500000000000   -0.000000000000   C
   0.100000000000    0.100000000000   -0.000000000000   C
   0.700000000000    0.700000000000   -0.000000000000   C
   0.400000000000    0.400000000000    0.000000000000   Hf_pv
   0.600000000000    0.600000000000    0.000000000000   Nb_sv
   0.000000000000    0.000000000000    0.000000000000   Ta_pv
   0.800000000000    0.800000000000    0.000000000000   Ti_sv
   0.200000000000    0.200000000000    0.000000000000   Zr_sv
```

HfNbTaTiZrC$_5$:configuration 19 - POSCAR

```
CHf_pvNb_svTa_pvTi_svZr_sv.AB_cF8_225_a_b.POCC   [HNF(5)=4/5= 1
     ↪ 0 0; 0 1 0; 0 4 5] DG=10   [BCT,BCT2,tI20] (STD_PRIM doi
     ↪ :10.1016/j.commatsci.2010.05.010) [Standard_Primitive
     ↪ Unit Cell Form]
1.000000
  -1.643859392183    1.643859392183    11.623841235295
   1.643859392183   -1.643859392183    11.623841235295
   1.643859392183    1.643859392183   -11.623841235295
5 1 1 1 1 1
Direct(10) [A5B1C1D1E1F1]
   0.300000000000    0.300000000000   -0.000000000000   C
   0.900000000000    0.900000000000   -0.000000000000   C
   0.500000000000    0.500000000000   -0.000000000000   C
   0.100000000000    0.100000000000   -0.000000000000   C
   0.700000000000    0.700000000000   -0.000000000000   C
   0.400000000000    0.400000000000    0.000000000000   Hf_pv
   0.000000000000    0.000000000000    0.000000000000   Nb_sv
   0.200000000000    0.200000000000    0.000000000000   Ta_pv
   0.600000000000    0.600000000000    0.000000000000   Ti_sv
   0.800000000000    0.800000000000    0.000000000000   Zr_sv
```

HfNbTaTiZrC$_5$:configuration 20 - POSCAR

```
CHf_pvNb_svTa_pvTi_svZr_sv.AB_cF8_225_a_b.POCC   [HNF(5)=4/5= 1
     ↪ 0 0; 0 1 0; 0 4 5] DG=10   [BCT,BCT2,tI20] (STD_PRIM doi
     ↪ :10.1016/j.commatsci.2010.05.010) [Standard_Primitive
     ↪ Unit Cell Form]
1.000000
  -1.643859392183    1.643859392183    11.623841235295
   1.643859392183   -1.643859392183    11.623841235295
   1.643859392183    1.643859392183   -11.623841235295
5 1 1 1 1 1
Direct(10) [A5B1C1D1E1F1]
   0.300000000000    0.300000000000   -0.000000000000   C
   0.900000000000    0.900000000000   -0.000000000000   C
   0.500000000000    0.500000000000   -0.000000000000   C
   0.100000000000    0.100000000000   -0.000000000000   C
   0.700000000000    0.700000000000   -0.000000000000   C
   0.400000000000    0.400000000000    0.000000000000   Hf_pv
   0.000000000000    0.000000000000    0.000000000000   Nb_sv
   0.600000000000    0.600000000000    0.000000000000   Ta_pv
   0.800000000000    0.800000000000    0.000000000000   Ti_sv
   0.200000000000    0.200000000000    0.000000000000   Zr_sv
```

HfNbTaTiZrC$_5$:configuration 21 - POSCAR

```
CHf_pvNb_svTa_pvTi_svZr_sv.AB_cF8_225_a_b.POCC   [HNF(5)=4/5= 1
     ↪ 0 0; 0 1 0; 0 4 5] DG=10   [BCT,BCT2,tI20] (STD_PRIM doi
     ↪ :10.1016/j.commatsci.2010.05.010) [Standard_Primitive
     ↪ Unit Cell Form]
1.000000
  -1.643859392183    1.643859392183    11.623841235295
   1.643859392183   -1.643859392183    11.623841235295
   1.643859392183    1.643859392183   -11.623841235295
5 1 1 1 1 1
Direct(10) [A5B1C1D1E1F1]
   0.300000000000    0.300000000000   -0.000000000000   C
   0.900000000000    0.900000000000   -0.000000000000   C
   0.500000000000    0.500000000000   -0.000000000000   C
   0.100000000000    0.100000000000   -0.000000000000   C
   0.700000000000    0.700000000000   -0.000000000000   C
   0.400000000000    0.400000000000    0.000000000000   Hf_pv
   0.000000000000    0.000000000000    0.000000000000   Nb_sv
   0.800000000000    0.800000000000    0.000000000000   Ta_pv
   0.200000000000    0.200000000000    0.000000000000   Ti_sv
   0.600000000000    0.600000000000    0.000000000000   Zr_sv
```

HfNbTaTiZrC$_5$:configuration 22 - POSCAR

```
CHf_pvNb_svTa_pvTi_svZr_sv.AB_cF8_225_a_b.POCC   [HNF(5)=4/5= 1
     ↪ 0 0; 0 1 0; 0 4 5] DG=10   [BCT,BCT2,tI20] (STD_PRIM doi
     ↪ :10.1016/j.commatsci.2010.05.010) [Standard_Primitive
     ↪ Unit Cell Form]
1.000000
  -1.605944110189    1.605944110189    11.324008725569
   1.605944110189   -1.605944110189    11.324008725569
   1.605944110189    1.605944110189   -11.324008725569
5 1 1 1 1 1
Direct(10) [A5B1C1D1E1F1]
   0.703511099323    0.703511099323    0.000000000000   C
   0.096497049699    0.096497049699    0.000000000000   C
   0.497474042178    0.497474042178    0.000000000000   C
   0.898551665357    0.898551665357   -0.000000000000   C
   0.303558838483    0.303558838483    0.000000000000   C
   0.599351988059    0.599351988059    0.000000000000   Hf_pv
   0.999098470681    0.999098470681    0.000000000000   Nb_sv
   0.397623071705    0.397623071705    0.000000000000   Ta_pv
   0.801433043455    0.801433043455    0.000000000000   Ti_sv
   0.202900731060    0.202900731060    0.000000000000   Zr_sv
```

HfNbTaTiZrC$_5$:configuration 23 - POSCAR

```
CHf_pvNb_svTa_pvTi_svZr_sv.AB_cF8_225_a_b.POCC   [HNF(5)=4/5= 1
     ↪ 0 0; 0 1 0; 0 4 5] DG=10   [BCT,BCT2,tI20] (STD_PRIM doi
     ↪ :10.1016/j.commatsci.2010.05.010) [Standard_Primitive
     ↪ Unit Cell Form]
1.000000
  -1.643859392183    1.643859392183    11.623841235295
   1.643859392183   -1.643859392183    11.623841235295
   1.643859392183    1.643859392183   -11.623841235295
5 1 1 1 1 1
Direct(10) [A5B1C1D1E1F1]
   0.300000000000    0.300000000000   -0.000000000000   C
   0.900000000000    0.900000000000   -0.000000000000   C
   0.500000000000    0.500000000000   -0.000000000000   C
   0.100000000000    0.100000000000   -0.000000000000   C
   0.700000000000    0.700000000000   -0.000000000000   C
   0.400000000000    0.400000000000    0.000000000000   Hf_pv
   0.000000000000    0.000000000000    0.000000000000   Nb_sv
   0.200000000000    0.200000000000    0.000000000000   Ta_pv
   0.800000000000    0.800000000000    0.000000000000   Ti_sv
   0.600000000000    0.600000000000    0.000000000000   Zr_sv
```

HfNbTaTiZrC$_5$:configuration 24 - POSCAR

```
CHf_pvNb_svTa_pvTi_svZr_sv.AB_cF8_225_a_b.POCC   [HNF(5)=2/5= 1
     ↪ 0 0; 0 1 0; 0 1 5] DG=10   [ORCI,ORCI,oI20] (STD_PRIM
     ↪ doi:10.1016/j.commatsci.2010.05.010) [
     ↪ Standard_Primitive Unit Cell Form]
1.000000
  -1.643859392183    2.324768247059    8.219296960913
   1.643859392183   -2.324768247059    8.219296960913
   1.643859392183    2.324768247059   -8.219296960913
5 1 1 1 1 1
Direct(10) [A5B1C1D1E1F1]
   0.500000000000   -0.000000000000    0.500000000000   C
   0.900000000000    0.400000000000    0.500000000000   C
   0.300000000000    0.800000000000    0.500000000000   C
   0.700000000000    0.200000000000    0.500000000000   C
   0.100000000000    0.600000000000    0.500000000000   C
   0.600000000000    0.600000000000    0.000000000000   Hf_pv
   0.000000000000    0.000000000000    0.000000000000   Nb_sv
   0.200000000000    0.200000000000    0.000000000000   Ta_pv
   0.400000000000    0.400000000000    0.000000000000   Ti_sv
   0.800000000000    0.800000000000    0.000000000000   Zr_sv
```

HfNbTaTiZrC$_5$:configuration 25 - POSCAR

```
CHf_pvNb_svTa_pvTi_svZr_sv.AB_cF8_225_a_b.POCC   [HNF(5)=2/5= 1
     ↪ 0 0; 0 1 0; 0 1 5] DG=10   [ORCI,ORCI,oI20] (STD_PRIM
     ↪ doi:10.1016/j.commatsci.2010.05.010) [
     ↪ Standard_Primitive Unit Cell Form]
1.000000
  -1.643859392183    2.324768247059    8.219296960913
   1.643859392183   -2.324768247059    8.219296960913
   1.643859392183    2.324768247059   -8.219296960913
5 1 1 1 1 1
Direct(10) [A5B1C1D1E1F1]
   0.500000000000   -0.000000000000    0.500000000000   C
   0.900000000000    0.400000000000    0.500000000000   C
   0.300000000000    0.800000000000    0.500000000000   C
   0.700000000000    0.200000000000    0.500000000000   C
   0.100000000000    0.600000000000    0.500000000000   C
   0.600000000000    0.600000000000    0.000000000000   Hf_pv
   0.400000000000    0.400000000000    0.000000000000   Nb_sv
   0.000000000000    0.000000000000    0.000000000000   Ta_pv
   0.800000000000    0.800000000000    0.000000000000   Ti_sv
   0.200000000000    0.200000000000    0.000000000000   Zr_sv
```

HfNbTaTiZrC$_5$:configuration 26 - POSCAR

```
CHf_pvNb_svTa_pvTi_svZr_sv.AB_cF8_225_a_b.POCC   [HNF(5)=4/5= 1
     ↪ 0 0; 0 1 0; 0 4 5] DG=10   [BCT,BCT2,tI20] (STD_PRIM doi
     ↪ :10.1016/j.commatsci.2010.05.010) [Standard_Primitive
     ↪ Unit Cell Form]
```

```
1.000000
  -1.643859392183    1.643859392183    11.623841235295
   1.643859392183   -1.643859392183    11.623841235295
   1.643859392183    1.643859392183   -11.623841235295
5 1 1 1 1 1
Direct(10) [A5B1C1D1E1F1]
   0.300000000000    0.300000000000   -0.000000000000   C
   0.900000000000    0.900000000000   -0.000000000000   C
   0.500000000000    0.500000000000   -0.000000000000   C
   0.100000000000    0.100000000000   -0.000000000000   C
   0.700000000000    0.700000000000   -0.000000000000   C
   0.400000000000    0.400000000000    0.000000000000   Hf_pv
   0.600000000000    0.600000000000    0.000000000000   Nb_sv
   0.200000000000    0.200000000000    0.000000000000   Ta_pv
   0.800000000000    0.800000000000    0.000000000000   Ti_sv
   0.000000000000    0.000000000000    0.000000000000   Zr_sv
```

HfNbTaTiZrC$_5$:configuration 27 - POSCAR

```
CHf_pvNb_svTa_pvTi_svZr_sv.AB_cF8_225_a_b.POCC   [HNF(5)=2/5= 1
     ↪  0 0; 0 1 0; 0 1 5] DG=10   [ORCI,ORCI,oI20]  (STD_PRIM
     ↪  doi:10.1016/j.commatsci.2010.05.010) [
     ↪  Standard_Primitive Unit Cell Form]
1.000000
  -1.643859392183    2.324768247059    8.219296960913
   1.643859392183   -2.324768247059    8.219296960913
   1.643859392183    2.324768247059   -8.219296960913
5 1 1 1 1 1
Direct(10) [A5B1C1D1E1F1]
   0.500000000000   -0.000000000000    0.500000000000   C
   0.900000000000    0.400000000000    0.500000000000   C
   0.300000000000    0.800000000000    0.500000000000   C
   0.700000000000    0.200000000000    0.500000000000   C
   0.100000000000    0.600000000000    0.500000000000   C
   0.600000000000    0.600000000000    0.000000000000   Hf_pv
   0.400000000000    0.400000000000    0.000000000000   Nb_sv
   0.200000000000    0.200000000000    0.000000000000   Ta_pv
   0.000000000000    0.000000000000    0.000000000000   Ti_sv
   0.800000000000    0.800000000000    0.000000000000   Zr_sv
```

HfNbTaTiZrC$_5$:configuration 28 - POSCAR

```
CHf_pvNb_svTa_pvTi_svZr_sv.AB_cF8_225_a_b.POCC   [HNF(5)=2/5= 1
     ↪  0 0; 0 1 0; 0 1 5] DG=10   [ORCI,ORCI,oI20]  (STD_PRIM
     ↪  doi:10.1016/j.commatsci.2010.05.010) [
     ↪  Standard_Primitive Unit Cell Form]
1.000000
  -1.643859392183    2.324768247059    8.219296960913
   1.643859392183   -2.324768247059    8.219296960913
   1.643859392183    2.324768247059   -8.219296960913
5 1 1 1 1 1
Direct(10) [A5B1C1D1E1F1]
   0.500000000000   -0.000000000000    0.500000000000   C
   0.900000000000    0.400000000000    0.500000000000   C
   0.300000000000    0.800000000000    0.500000000000   C
   0.700000000000    0.200000000000    0.500000000000   C
   0.100000000000    0.600000000000    0.500000000000   C
   0.600000000000    0.600000000000    0.000000000000   Hf_pv
   0.400000000000    0.400000000000    0.000000000000   Nb_sv
   0.800000000000    0.800000000000    0.000000000000   Ta_pv
   0.000000000000    0.000000000000    0.000000000000   Ti_sv
   0.200000000000    0.200000000000    0.000000000000   Zr_sv
```

HfNbTaTiZrC$_5$:configuration 29 - POSCAR

```
CHf_pvNb_svTa_pvTi_svZr_sv.AB_cF8_225_a_b.POCC   [HNF(5)=2/5= 1
     ↪  0 0; 0 1 0; 0 1 5] DG=10   [ORCI,ORCI,oI20]  (STD_PRIM
     ↪  doi:10.1016/j.commatsci.2010.05.010) [
     ↪  Standard_Primitive Unit Cell Form]
1.000000
  -1.643859392183    2.324768247059    8.219296960913
   1.643859392183   -2.324768247059    8.219296960913
   1.643859392183    2.324768247059   -8.219296960913
5 1 1 1 1 1
Direct(10) [A5B1C1D1E1F1]
   0.500000000000   -0.000000000000    0.500000000000   C
   0.900000000000    0.400000000000    0.500000000000   C
   0.300000000000    0.800000000000    0.500000000000   C
   0.700000000000    0.200000000000    0.500000000000   C
   0.100000000000    0.600000000000    0.500000000000   C
   0.600000000000    0.600000000000    0.000000000000   Hf_pv
   0.400000000000    0.400000000000    0.000000000000   Nb_sv
   0.200000000000    0.200000000000    0.000000000000   Ta_pv
   0.800000000000    0.800000000000    0.000000000000   Ti_sv
   0.000000000000    0.000000000000    0.000000000000   Zr_sv
```

HfNbTaTiZrC$_5$:configuration 30 - POSCAR

```
CHf_pvNb_svTa_pvTi_svZr_sv.AB_cF8_225_a_b.POCC   [HNF(5)=3/5= 1
     ↪  0 0; 0 1 0; 0 2 5] DG=10   [MCLC,MCLC1,mS20]  (STD_PRIM
     ↪  doi:10.1016/j.commatsci.2010.05.010) [
     ↪  Standard_Primitive Unit Cell Form]
1.000000
   1.643859392183    5.452064811971    0.000000000000
  -1.643859392183    5.452064811971    0.000000000000
   0.000000000000    3.965138045070    7.009440000025
5 1 1 1 1 1
Direct(10) [A5B1C1D1E1F1]
   0.200000000000    0.200000000000    0.700000000000   C
   0.600000000000    0.600000000000    0.100000000000   C
  -0.000000000000    0.000000000000    0.500000000000   C
   0.400000000000    0.400000000000    0.900000000000   C
   0.800000000000    0.800000000000    0.300000000000   C
   0.600000000000    0.600000000000    0.600000000000   Hf_pv
   0.400000000000    0.400000000000    0.400000000000   Nb_sv
   0.800000000000    0.800000000000    0.800000000000   Ta_pv
   0.200000000000    0.200000000000    0.200000000000   Ti_sv
   0.000000000000    0.000000000000    0.000000000000   Zr_sv
```

HfNbTaTiZrC$_5$:configuration 31 - POSCAR

```
CHf_pvNb_svTa_pvTi_svZr_sv.AB_cF8_225_a_b.POCC   [HNF(5)=3/5= 1
     ↪  0 0; 0 1 0; 0 2 5] DG=10   [MCLC,MCLC1,mS20]  (STD_PRIM
     ↪  doi:10.1016/j.commatsci.2010.05.010) [
     ↪  Standard_Primitive Unit Cell Form]
1.000000
   1.643859392183    5.452064811971    0.000000000000
  -1.643859392183    5.452064811971    0.000000000000
   0.000000000000    3.965138045070    7.009440000025
5 1 1 1 1 1
Direct(10) [A5B1C1D1E1F1]
   0.200000000000    0.200000000000    0.700000000000   C
   0.600000000000    0.600000000000    0.100000000000   C
  -0.000000000000    0.000000000000    0.500000000000   C
   0.400000000000    0.400000000000    0.900000000000   C
   0.800000000000    0.800000000000    0.300000000000   C
   0.600000000000    0.600000000000    0.600000000000   Hf_pv
   0.000000000000    0.000000000000    0.000000000000   Nb_sv
   0.800000000000    0.800000000000    0.800000000000   Ta_pv
   0.200000000000    0.200000000000    0.200000000000   Ti_sv
   0.400000000000    0.400000000000    0.400000000000   Zr_sv
```

HfNbTaTiZrC$_5$:configuration 32 - POSCAR

```
CHf_pvNb_svTa_pvTi_svZr_sv.AB_cF8_225_a_b.POCC   [HNF(5)=2/5= 1
     ↪  0 0; 0 1 0; 0 1 5] DG=10   [ORCI,ORCI,oI20]  (STD_PRIM
     ↪  doi:10.1016/j.commatsci.2010.05.010) [
     ↪  Standard_Primitive Unit Cell Form]
1.000000
  -1.643859392183    2.324768247059    8.219296960913
   1.643859392183   -2.324768247059    8.219296960913
   1.643859392183    2.324768247059   -8.219296960913
5 1 1 1 1 1
Direct(10) [A5B1C1D1E1F1]
   0.500000000000   -0.000000000000    0.500000000000   C
   0.900000000000    0.400000000000    0.500000000000   C
   0.300000000000    0.800000000000    0.500000000000   C
   0.700000000000    0.200000000000    0.500000000000   C
   0.100000000000    0.600000000000    0.500000000000   C
   0.600000000000    0.600000000000    0.000000000000   Hf_pv
   0.000000000000    0.000000000000    0.000000000000   Nb_sv
   0.800000000000    0.800000000000    0.000000000000   Ta_pv
   0.400000000000    0.400000000000    0.000000000000   Ti_sv
   0.200000000000    0.200000000000    0.000000000000   Zr_sv
```

HfNbTaTiZrC$_5$:configuration 33 - POSCAR

```
CHf_pvNb_svTa_pvTi_svZr_sv.AB_cF8_225_a_b.POCC   [HNF(5)=2/5= 1
     ↪  0 0; 0 1 0; 0 1 5] DG=10   [ORCI,ORCI,oI20]  (STD_PRIM
     ↪  doi:10.1016/j.commatsci.2010.05.010) [
     ↪  Standard_Primitive Unit Cell Form]
1.000000
  -1.643859392183    2.324768247059    8.219296960913
   1.643859392183   -2.324768247059    8.219296960913
   1.643859392183    2.324768247059   -8.219296960913
5 1 1 1 1 1
Direct(10) [A5B1C1D1E1F1]
   0.500000000000   -0.000000000000    0.500000000000   C
   0.900000000000    0.400000000000    0.500000000000   C
   0.300000000000    0.800000000000    0.500000000000   C
   0.700000000000    0.200000000000    0.500000000000   C
   0.100000000000    0.600000000000    0.500000000000   C
   0.600000000000    0.600000000000    0.000000000000   Hf_pv
   0.400000000000    0.400000000000    0.000000000000   Nb_sv
   0.000000000000    0.000000000000    0.000000000000   Ta_pv
   0.200000000000    0.200000000000    0.000000000000   Ti_sv
   0.800000000000    0.800000000000    0.000000000000   Zr_sv
```

HfNbTaTiZrC$_5$:configuration 34 - POSCAR

```
CHf_pvNb_svTa_pvTi_svZr_sv.AB_cF8_225_a_b.POCC   [HNF(5)=3/5= 1
     ↪  0 0; 0 1 0; 0 2 5] DG=10   [MCLC,MCLC1,mS20]  (STD_PRIM
     ↪  doi:10.1016/j.commatsci.2010.05.010) [
     ↪  Standard_Primitive Unit Cell Form]
1.000000
   1.643859392183    5.452064811971    0.000000000000
  -1.643859392183    5.452064811971    0.000000000000
   0.000000000000    3.965138045070    7.009440000025
5 1 1 1 1 1
Direct(10) [A5B1C1D1E1F1]
   0.200000000000    0.200000000000    0.700000000000   C
   0.600000000000    0.600000000000    0.100000000000   C
  -0.000000000000    0.000000000000    0.500000000000   C
   0.400000000000    0.400000000000    0.900000000000   C
   0.800000000000    0.800000000000    0.300000000000   C
   0.600000000000    0.600000000000    0.600000000000   Hf_pv
   0.000000000000    0.000000000000    0.000000000000   Nb_sv
   0.400000000000    0.400000000000    0.400000000000   Ta_pv
   0.800000000000    0.800000000000    0.800000000000   Ti_sv
   0.200000000000    0.200000000000    0.200000000000   Zr_sv
```

HfNbTaTiZrC$_5$:configuration 35 - POSCAR

```
CHf_pvNb_svTa_pvTi_svZr_sv.AB_cF8_225_a_b.POCC   [HNF(5)=2/5= 1
     ↪  0 0; 0 1 0; 0 1 5] DG=10   [ORCI,ORCI,oI20]  (STD_PRIM
     ↪  doi:10.1016/j.commatsci.2010.05.010) [
     ↪  Standard_Primitive Unit Cell Form]
1.000000
  -1.643859392183    2.324768247059    8.219296960913
   1.643859392183   -2.324768247059    8.219296960913
   1.643859392183    2.324768247059   -8.219296960913
5 1 1 1 1 1
Direct(10) [A5B1C1D1E1F1]
   0.500000000000   -0.000000000000    0.500000000000   C
   0.900000000000    0.400000000000    0.500000000000   C
   0.300000000000    0.800000000000    0.500000000000   C
   0.700000000000    0.200000000000    0.500000000000   C
   0.100000000000    0.600000000000    0.500000000000   C
```

```
    0.600000000000    0.600000000000    0.000000000000   Hf_pv
    0.000000000000    0.000000000000    0.000000000000   Nb_sv
    0.400000000000    0.400000000000    0.000000000000   Ta_pv
    0.200000000000    0.200000000000    0.000000000000   Ti_sv
    0.800000000000    0.800000000000    0.000000000000   Zr_sv
```

HfNbTaTiZrC_5:configuration 36 - POSCAR

```
CHf_pvNb_svTa_pvTi_svZr_sv.AB_cF8_225_a_b.POCC   [HNF(5)=3/5= 1
     ↪ 0 0; 0 1 0; 0 2 5] DG=10   [MCLC,MCLC1,mS20]  (STD_PRIM
     ↪ doi:10.1016/j.commatsci.2010.05.010) [
     ↪ Standard_Primitive Unit Cell Form]
1.000000
    1.643859392183    5.452064811971    0.000000000000
   -1.643859392183    5.452064811971    0.000000000000
    0.000000000000    3.965138045070    7.009440000025
5 1 1 1 1 1
Direct(10) [A5B1C1D1E1F1]
    0.200000000000    0.200000000000    0.700000000000   C
    0.600000000000    0.600000000000    0.100000000000   C
   -0.000000000000    0.000000000000    0.500000000000   C
    0.400000000000    0.400000000000    0.900000000000   C
    0.800000000000    0.800000000000    0.300000000000   C
    0.600000000000    0.600000000000    0.600000000000   Hf_pv
    0.000000000000    0.000000000000    0.000000000000   Nb_sv
    0.200000000000    0.200000000000    0.200000000000   Ta_pv
    0.800000000000    0.800000000000    0.800000000000   Ti_sv
    0.400000000000    0.400000000000    0.400000000000   Zr_sv
```

HfNbTaTiZrC_5:configuration 37 - POSCAR

```
CHf_pvNb_svTa_pvTi_svZr_sv.AB_cF8_225_a_b.POCC   [HNF(5)=3/5= 1
     ↪ 0 0; 0 1 0; 0 2 5] DG=10   [MCLC,MCLC1,mS20]  (STD_PRIM
     ↪ doi:10.1016/j.commatsci.2010.05.010) [
     ↪ Standard_Primitive Unit Cell Form]
1.000000
    1.643859392183    5.452064811971    0.000000000000
   -1.643859392183    5.452064811971    0.000000000000
    0.000000000000    3.965138045070    7.009440000025
5 1 1 1 1 1
Direct(10) [A5B1C1D1E1F1]
    0.200000000000    0.200000000000    0.700000000000   C
    0.600000000000    0.600000000000    0.100000000000   C
   -0.000000000000    0.000000000000    0.500000000000   C
    0.400000000000    0.400000000000    0.900000000000   C
    0.800000000000    0.800000000000    0.300000000000   C
    0.600000000000    0.600000000000    0.600000000000   Hf_pv
    0.000000000000    0.000000000000    0.000000000000   Nb_sv
    0.400000000000    0.400000000000    0.400000000000   Ta_pv
    0.200000000000    0.200000000000    0.200000000000   Ti_sv
    0.800000000000    0.800000000000    0.800000000000   Zr_sv
```

HfNbTaTiZrC_5:configuration 38 - POSCAR

```
CHf_pvNb_svTa_pvTi_svZr_sv.AB_cF8_225_a_b.POCC   [HNF(5)=3/5= 1
     ↪ 0 0; 0 1 0; 0 2 5] DG=10   [MCLC,MCLC1,mS20]  (STD_PRIM
     ↪ doi:10.1016/j.commatsci.2010.05.010) [
     ↪ Standard_Primitive Unit Cell Form]
1.000000
    1.643859392183    5.452064811971    0.000000000000
   -1.643859392183    5.452064811971    0.000000000000
    0.000000000000    3.965138045070    7.009440000025
5 1 1 1 1 1
Direct(10) [A5B1C1D1E1F1]
    0.200000000000    0.200000000000    0.700000000000   C
    0.600000000000    0.600000000000    0.100000000000   C
   -0.000000000000    0.000000000000    0.500000000000   C
    0.400000000000    0.400000000000    0.900000000000   C
    0.800000000000    0.800000000000    0.300000000000   C
    0.600000000000    0.600000000000    0.600000000000   Hf_pv
    0.000000000000    0.000000000000    0.000000000000   Nb_sv
    0.800000000000    0.800000000000    0.800000000000   Ta_pv
    0.400000000000    0.400000000000    0.400000000000   Ti_sv
    0.200000000000    0.200000000000    0.200000000000   Zr_sv
```

HfNbTaTiZrC_5:configuration 39 - POSCAR

```
CHf_pvNb_svTa_pvTi_svZr_sv.AB_cF8_225_a_b.POCC   [HNF(5)=3/5= 1
     ↪ 0 0; 0 1 0; 0 2 5] DG=10   [MCLC,MCLC1,mS20]  (STD_PRIM
     ↪ doi:10.1016/j.commatsci.2010.05.010) [
     ↪ Standard_Primitive Unit Cell Form]
1.000000
    1.643859392183    5.452064811971    0.000000000000
   -1.643859392183    5.452064811971    0.000000000000
    0.000000000000    3.965138045070    7.009440000025
5 1 1 1 1 1
Direct(10) [A5B1C1D1E1F1]
    0.200000000000    0.200000000000    0.700000000000   C
    0.600000000000    0.600000000000    0.100000000000   C
   -0.000000000000    0.000000000000    0.500000000000   C
    0.400000000000    0.400000000000    0.900000000000   C
    0.800000000000    0.800000000000    0.300000000000   C
    0.600000000000    0.600000000000    0.600000000000   Hf_pv
    0.400000000000    0.400000000000    0.400000000000   Nb_sv
    0.000000000000    0.000000000000    0.000000000000   Ta_pv
    0.200000000000    0.200000000000    0.200000000000   Ti_sv
    0.800000000000    0.800000000000    0.800000000000   Zr_sv
```

HfNbTaTiZrC_5:configuration 40 - POSCAR

```
CHf_pvNb_svTa_pvTi_svZr_sv.AB_cF8_225_a_b.POCC   [HNF(5)=3/5= 1
     ↪ 0 0; 0 1 0; 0 2 5] DG=10   [MCLC,MCLC1,mS20]  (STD_PRIM
     ↪ doi:10.1016/j.commatsci.2010.05.010) [
     ↪ Standard_Primitive Unit Cell Form]
1.000000
    1.643859392183    5.452064811971    0.000000000000
   -1.643859392183    5.452064811971    0.000000000000
    0.000000000000    3.965138045070    7.009440000025
5 1 1 1 1 1
Direct(10) [A5B1C1D1E1F1]
    0.200000000000    0.200000000000    0.700000000000   C
    0.600000000000    0.600000000000    0.100000000000   C
   -0.000000000000    0.000000000000    0.500000000000   C
    0.400000000000    0.400000000000    0.900000000000   C
    0.800000000000    0.800000000000    0.300000000000   C
    0.600000000000    0.600000000000    0.600000000000   Hf_pv
    0.400000000000    0.400000000000    0.400000000000   Nb_sv
    0.200000000000    0.200000000000    0.200000000000   Ta_pv
    0.800000000000    0.800000000000    0.800000000000   Ti_sv
    0.000000000000    0.000000000000    0.000000000000   Zr_sv
```

HfNbTaTiZrC_5:configuration 41 - POSCAR

```
CHf_pvNb_svTa_pvTi_svZr_sv.AB_cF8_225_a_b.POCC   [HNF(5)=3/5= 1
     ↪ 0 0; 0 1 0; 0 2 5] DG=10   [MCLC,MCLC1,mS20]  (STD_PRIM
     ↪ doi:10.1016/j.commatsci.2010.05.010) [
     ↪ Standard_Primitive Unit Cell Form]
1.000000
    1.643859392183    5.452064811971    0.000000000000
   -1.643859392183    5.452064811971    0.000000000000
    0.000000000000    3.965138045070    7.009440000025
5 1 1 1 1 1
Direct(10) [A5B1C1D1E1F1]
    0.200000000000    0.200000000000    0.700000000000   C
    0.600000000000    0.600000000000    0.100000000000   C
   -0.000000000000    0.000000000000    0.500000000000   C
    0.400000000000    0.400000000000    0.900000000000   C
    0.800000000000    0.800000000000    0.300000000000   C
    0.600000000000    0.600000000000    0.600000000000   Hf_pv
    0.400000000000    0.400000000000    0.400000000000   Nb_sv
    0.800000000000    0.800000000000    0.800000000000   Ta_pv
    0.000000000000    0.000000000000    0.000000000000   Ti_sv
    0.200000000000    0.200000000000    0.200000000000   Zr_sv
```

HfNbTaTiZrC_5:configuration 42 - POSCAR

```
CHf_pvNb_svTa_pvTi_svZr_sv.AB_cF8_225_a_b.POCC   [HNF(5)=2/5= 1
     ↪ 0 0; 0 1 0; 0 1 5] DG=10   [ORCI,ORCI,oI20]  (STD_PRIM
     ↪ doi:10.1016/j.commatsci.2010.05.010) [
     ↪ Standard_Primitive Unit Cell Form]
1.000000
   -1.643859392183    2.324768247059    8.219296960913
    1.643859392183   -2.324768247059    8.219296960913
    1.643859392183    2.324768247059   -8.219296960913
5 1 1 1 1 1
Direct(10) [A5B1C1D1E1F1]
    0.500000000000   -0.000000000000    0.500000000000   C
    0.900000000000    0.400000000000    0.500000000000   C
    0.300000000000    0.800000000000    0.500000000000   C
    0.700000000000    0.200000000000    0.500000000000   C
    0.100000000000    0.600000000000    0.500000000000   C
    0.600000000000    0.600000000000    0.000000000000   Hf_pv
    0.000000000000    0.000000000000    0.000000000000   Nb_sv
    0.200000000000    0.200000000000    0.000000000000   Ta_pv
    0.800000000000    0.800000000000    0.000000000000   Ti_sv
    0.400000000000    0.400000000000    0.000000000000   Zr_sv
```

HfNbTaTiZrC_5:configuration 43 - POSCAR

```
CHf_pvNb_svTa_pvTi_svZr_sv.AB_cF8_225_a_b.POCC   [HNF(5)=3/5= 1
     ↪ 0 0; 0 1 0; 0 2 5] DG=10   [MCLC,MCLC1,mS20]  (STD_PRIM
     ↪ doi:10.1016/j.commatsci.2010.05.010) [
     ↪ Standard_Primitive Unit Cell Form]
1.000000
    1.643859392183    5.452064811971    0.000000000000
   -1.643859392183    5.452064811971    0.000000000000
    0.000000000000    3.965138045070    7.009440000025
5 1 1 1 1 1
Direct(10) [A5B1C1D1E1F1]
    0.200000000000    0.200000000000    0.700000000000   C
    0.600000000000    0.600000000000    0.100000000000   C
   -0.000000000000    0.000000000000    0.500000000000   C
    0.400000000000    0.400000000000    0.900000000000   C
    0.800000000000    0.800000000000    0.300000000000   C
    0.600000000000    0.600000000000    0.600000000000   Hf_pv
    0.400000000000    0.400000000000    0.400000000000   Nb_sv
    0.200000000000    0.200000000000    0.200000000000   Ta_pv
    0.000000000000    0.000000000000    0.000000000000   Ti_sv
    0.800000000000    0.800000000000    0.800000000000   Zr_sv
```

HfNbTaTiZrC_5:configuration 44 - POSCAR

```
CHf_pvNb_svTa_pvTi_svZr_sv.AB_cF8_225_a_b.POCC   [HNF(5)=3/5= 1
     ↪ 0 0; 0 1 0; 0 2 5] DG=10   [MCLC,MCLC1,mS20]  (STD_PRIM
     ↪ doi:10.1016/j.commatsci.2010.05.010) [
     ↪ Standard_Primitive Unit Cell Form]
1.000000
    1.643859392183    5.452064811971    0.000000000000
   -1.643859392183    5.452064811971    0.000000000000
    0.000000000000    3.965138045070    7.009440000025
5 1 1 1 1 1
Direct(10) [A5B1C1D1E1F1]
    0.200000000000    0.200000000000    0.700000000000   C
    0.600000000000    0.600000000000    0.100000000000   C
   -0.000000000000    0.000000000000    0.500000000000   C
    0.400000000000    0.400000000000    0.900000000000   C
    0.800000000000    0.800000000000    0.300000000000   C
    0.600000000000    0.600000000000    0.600000000000   Hf_pv
    0.400000000000    0.400000000000    0.400000000000   Nb_sv
    0.000000000000    0.000000000000    0.000000000000   Ta_pv
    0.800000000000    0.800000000000    0.800000000000   Ti_sv
    0.200000000000    0.200000000000    0.200000000000   Zr_sv
```

HfNbTaTiZrC_5:configuration 45 - POSCAR

```
CHf_pvNb_svTa_pvTi_svZr_sv.AB_cF8_225_a_b.POCC   [HNF(5)=3/5= 1
     ↪ 0 0; 0 1 0; 0 2 5] DG=10   [MCLC,MCLC1,mS20]  (STD_PRIM
     ↪ doi:10.1016/j.commatsci.2010.05.010) [
     ↪ Standard_Primitive Unit Cell Form]
1.000000
   1.643859392183    5.452064811971    0.000000000000
  -1.643859392183    5.452064811971    0.000000000000
   0.000000000000    3.965138045070    7.009440000025
5 1 1 1 1 1
Direct(10) [A5B1C1D1E1F1]
   0.200000000000    0.200000000000    0.700000000000  C
   0.600000000000    0.600000000000    0.100000000000  C
  -0.000000000000    0.000000000000    0.500000000000  C
   0.400000000000    0.400000000000    0.900000000000  C
   0.800000000000    0.800000000000    0.300000000000  C
   0.600000000000    0.600000000000    0.600000000000  Hf_pv
   0.000000000000    0.000000000000    0.000000000000  Nb_sv
   0.200000000000    0.200000000000    0.200000000000  Ta_pv
   0.400000000000    0.400000000000    0.400000000000  Ti_sv
   0.800000000000    0.800000000000    0.800000000000  Zr_sv
```

HfNbTaTiZrC_5:configuration 46 - POSCAR

```
CHf_pvNb_svTa_pvTi_svZr_sv.AB_cF8_225_a_b.POCC   [HNF(5)=2/5= 1
     ↪ 0 0; 0 1 0; 0 1 5] DG=10   [ORCI,ORCI,oI20]  (STD_PRIM
     ↪ doi:10.1016/j.commatsci.2010.05.010) [
     ↪ Standard_Primitive Unit Cell Form]
1.000000
  -1.643859392183    2.324768247059    8.219296960913
   1.643859392183   -2.324768247059    8.219296960913
   1.643859392183    2.324768247059   -8.219296960913
5 1 1 1 1 1
Direct(10) [A5B1C1D1E1F1]
   0.500000000000   -0.000000000000    0.500000000000  C
   0.900000000000    0.400000000000    0.500000000000  C
   0.300000000000    0.800000000000    0.500000000000  C
   0.700000000000    0.200000000000    0.500000000000  C
   0.100000000000    0.600000000000    0.500000000000  C
   0.600000000000    0.600000000000    0.000000000000  Hf_pv
   0.000000000000    0.000000000000    0.000000000000  Nb_sv
   0.400000000000    0.400000000000    0.000000000000  Ta_pv
   0.800000000000    0.800000000000    0.000000000000  Ti_sv
   0.200000000000    0.200000000000    0.000000000000  Zr_sv
```

HfNbTaTiZrC_5:configuration 47 - POSCAR

```
CHf_pvNb_svTa_pvTi_svZr_sv.AB_cF8_225_a_b.POCC   [HNF(5)=2/5= 1
     ↪ 0 0; 0 1 0; 0 1 5] DG=10   [ORCI,ORCI,oI20]  (STD_PRIM
     ↪ doi:10.1016/j.commatsci.2010.05.010) [
     ↪ Standard_Primitive Unit Cell Form]
1.000000
  -1.643859392183    2.324768247059    8.219296960913
   1.643859392183   -2.324768247059    8.219296960913
   1.643859392183    2.324768247059   -8.219296960913
5 1 1 1 1 1
Direct(10) [A5B1C1D1E1F1]
   0.500000000000   -0.000000000000    0.500000000000  C
   0.900000000000    0.400000000000    0.500000000000  C
   0.300000000000    0.800000000000    0.500000000000  C
   0.700000000000    0.200000000000    0.500000000000  C
   0.100000000000    0.600000000000    0.500000000000  C
   0.600000000000    0.600000000000    0.000000000000  Hf_pv
   0.000000000000    0.000000000000    0.000000000000  Nb_sv
   0.800000000000    0.800000000000    0.000000000000  Ta_pv
   0.200000000000    0.200000000000    0.000000000000  Ti_sv
   0.400000000000    0.400000000000    0.000000000000  Zr_sv
```

HfNbTaTiZrC_5:configuration 48 - POSCAR

```
CHf_pvNb_svTa_pvTi_svZr_sv.AB_cF8_225_a_b.POCC   [HNF(5)=2/5= 1
     ↪ 0 0; 0 1 0; 0 1 5] DG=10   [ORCI,ORCI,oI20]  (STD_PRIM
     ↪ doi:10.1016/j.commatsci.2010.05.010) [
     ↪ Standard_Primitive Unit Cell Form]
1.000000
  -1.643859392183    2.324768247059    8.219296960913
   1.643859392183   -2.324768247059    8.219296960913
   1.643859392183    2.324768247059   -8.219296960913
5 1 1 1 1 1
Direct(10) [A5B1C1D1E1F1]
   0.500000000000   -0.000000000000    0.500000000000  C
   0.900000000000    0.400000000000    0.500000000000  C
   0.300000000000    0.800000000000    0.500000000000  C
   0.700000000000    0.200000000000    0.500000000000  C
   0.100000000000    0.600000000000    0.500000000000  C
   0.600000000000    0.600000000000    0.000000000000  Hf_pv
   0.400000000000    0.400000000000    0.000000000000  Nb_sv
   0.800000000000    0.800000000000    0.000000000000  Ta_pv
   0.200000000000    0.200000000000    0.000000000000  Ti_sv
   0.000000000000    0.000000000000    0.000000000000  Zr_sv
```

HfNbTaTiZrC_5:configuration 49 - POSCAR

```
CHf_pvNb_svTa_pvTi_svZr_sv.AB_cF8_225_a_b.POCC   [HNF(5)=5/5= 1
     ↪ 0 0; 0 1 0; 1 2 5] DG=120   [MCLC,MCLC3,mS20]  (STD_PRIM
     ↪ doi:10.1016/j.commatsci.2010.05.010) [
     ↪ Standard_Primitive Unit Cell Form]
1.000000
   5.198339832357    2.324768247059    0.000000000000
  -5.198339832357    2.324768247059    0.000000000000
   0.000000000000    2.324768247059    5.198339832357
5 1 1 1 1 1
Direct(10) [A5B1C1D1E1F1]
   0.500000000000    0.500000000000    0.000000000000  C
   0.300000000000    0.100000000000    0.600000000000  C
   0.100000000000    0.700000000000    0.200000000000  C
   0.900000000000    0.300000000000    0.800000000000  C
   0.700000000000    0.900000000000    0.400000000000  C
   0.200000000000    0.400000000000    0.400000000000  Hf_pv
   0.000000000000    0.000000000000    0.000000000000  Nb_sv

   0.400000000000    0.800000000000    0.800000000000  Ta_pv
   0.800000000000    0.600000000000    0.600000000000  Ti_sv
   0.600000000000    0.200000000000    0.200000000000  Zr_sv
```

SUPPLEMENTARY REFERENCES

* These authors contributed equally to the work
† kvecchio@eng.ucsd.edu
‡ stefano@duke.edu

[1] Oses, C. *et al.* AFLOW-CHULL: Cloud-oriented platform for autonomous phase stability analysis. *J. Chem. Inf. Model.* **in press**, doi:10.1021/acs.jcim.8b00393 (2018).

[2] Oliver, W. C. & Pharr, G. M. An improved technique for determining hardness and elastic modulus using load and displacement sensing indentation experiments. *J. Mater. Res.* **7**, 1564–1583 (1992).

[3] Oliver, W. C. & Pharr, G. M. Measurement of hardness and elastic modulus by instrumented indentation: Advances in understanding and refinements to methodology. *J. Mater. Res.* **19**, 3–20 (2004).

[4] Chen, X.-Q., Niu, H., Li, D. & Li, Y. Modeling hardness of polycrystalline materials and bulk metallic glasses. *Intermetallics* **19**, 1275–1281 (2011).

[5] Toher, C. *et al.* Combining the AFLOW GIBBS and elastic libraries to efficiently and robustly screen thermomechanical properties of solids. *Phys. Rev. Materials* **1**, 015401 (2017).

[6] Yang, K., Oses, C. & Curtarolo, S. Modeling off-stoichiometry materials with a high-throughput *ab-initio* approach. *Chem. Mater.* **28**, 6484–6492 (2016).

[7] Kresse, G. & Furthmüller, J. Efficient iterative schemes for *ab initio* total-energy calculations using a plane-wave basis set. *Phys. Rev. B* **54**, 11169–11186 (1996).

[8] Mehl, M. J. *et al.* The AFLOW library of crystallographic prototypes: Part 1. *Comput. Mater. Sci.* **136**, S1–S828 (2017).
